# Synthesis and Anti-TMV Activity of Dialkyl/dibenzyl 2-((6-Substituted-benzo[d]thiazol-2-ylamino)(benzofuran-2-yl)methyl) Malonates

**DOI:** 10.3390/molecules181113623

**Published:** 2013-11-04

**Authors:** Han Xiao, Pengcheng Zheng, Chenghao Tang, Ying Xie, Fang Wu, Jie Song, Jun Zhao, Zhining Li, Meichuan Li

**Affiliations:** 1 Key Laboratory of Green Pesticide and Agricultural Bioengineering, Ministry of Education, Guizhou University, Guiyang 550025, China; E-Mails: zhengpc1986@163.com (P.Z.); chtang1122@163.com (C.T.); xieying0020@126.com (Y.X.); fangwu90@126.com (F.W.); songjie277@yeah.net (J.S.); zhaojun0020@126.com (J.Z.); aning072@126.com (Z.L.); lmcuvhn1987@163.com (M.L.); 2 School of Chemistry and Environmental Science, Guizhou Minzu University, Guiyang 550025, China

**Keywords:** benzofuran, benzothiazole, β-amino acid ester, anti-TMV activity

## Abstract

Starting from benzofuran-2-methanal, 6-substituted benzothiazole-2-amines and malonic esters, sixteen title compounds were designed and synthesized seeking to introduce anti-TMV activity. The structures of the newly synthesized compounds were confirmed by ^1^H-NMR, ^13^C-NMR, IR spectra, and MS (HREI) analysis. The bioassays identified some of these new compounds as having moderate to good anti-TMV activity. The compounds **5i** and **5m** have good antiviral activity against TMV with a curative rate of 52.23% and 54.41%, respectively, at a concentration of 0.5 mg/mL.

## 1. Introduction

Benzothiazoles have varied biological activities [1,2,3]. They are widely found in bioorganic and medicinal chemistry with applications in drug discovery and are still of great scientific interest nowadays [4]. Benzothiazole moieties are part of compounds showing numerous biological activities such as anti-bacterial, anti-microbial, anthelmintic, antitumor, anti-inflammatory properties [5,6,7,8]. Compounds containing benzofuran moieties are also widespread in Nature, with a broad spectrum of physiological bioactivities, used as pesticidal, anti-bacterial, insecticide, anti-tumor, anti-inflammatory [9,10,11], and so on.

β-Amino ester derivatives are key intermediate in the synthesis of β-lactam antibiotics, which are important components of many natural products and therapeutic agents [12,13,14]. Therefore, the asymmetric synthesis of β-amino ester derivatives has become a field of increasing interest in organic synthetic chemistry over the past few years [15,16,17,18,19,20].

As an extension study of our group’s research [19,21], we have now synthesized a series of novel β-amino ester derivatives containing benzofuran and benzothiazole units. The structures of these newly synthesized compounds were confirmed by ^1^H-NMR, ^13^C-NMR, IR spectra, and MS (HREI) analysis. Bioassays identified these new compounds as possessing weak to good antiviral activities.

## 2. Results and Discussion

### 2.1. Experimental Condition Optimization

**Scheme 1:**
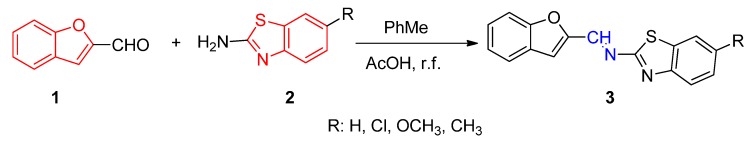
Synthesis of imines.

Our strategy is outlined in Scheme 1. Benzofuran-2-methanal (**1**) reacted with 6-substituted benzothiazoles **2a**–**d** under reflux conditions in toluene, with some acetic acid as catalyst affording the imines **3a**–**d**. After recrystallization by ethanol, the final compounds were isolated in good yields. All products **3a**–**d** were characterized by spectroscopic methods.

**Scheme 2:**
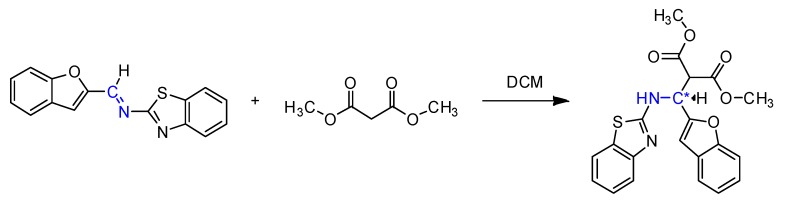
Screening of the reaction conditions.

According to the Scheme 2, we tested the influence of the reaction temperature, solvent, and reaction times. The reaction temperature had a pronounced effect on the yield, solvents such as THF, acetone, and toluene showed lower yields compared to DCM (Table 1). According to an optimized procedure, the best result was achieved at 35 °C in DCM.

**Table 1:** The optimization of reaction conditions.

Entry	Solvent	Temp. (°C)	Time (h)	Yield (%)
1	THF	r.t.	24	34
2	THF	r.f.	12	38
3	PhMe	r.t.	24	46
4	PhMe	r.f.	12	72
5	DCM	r.t.	24	62
6	DCM	35	10	78

**Scheme 3:**
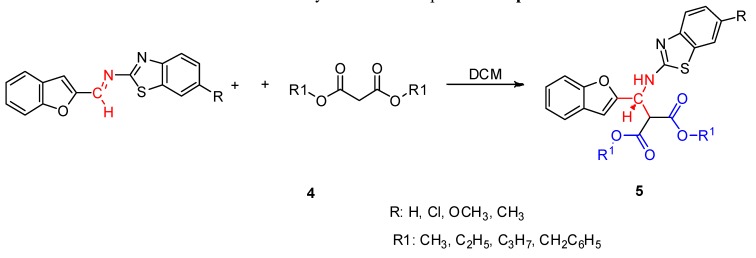
Synthesis of compounds **5a−p**.

Having established the ideal reaction conditions, the synthetic scope of the reaction was evaluated with different imines and malonic esters (Scheme 3). The results of our studies are summarized in Table 2. It can be seen that the reactions afforded good yields.

**Table 2:** The yields of the Mannich reactions of imines and malonic esters.

Entry	5	R	R^1^	Time(h)	Yield(% ^a^)
1	**5a**	6-H	-CH_3_	12	82
2	**5b**	6-H	-C_2_H_5_	12	80
3	**5c**	6-H	-C_3_H_7_	12	78
4	**5d**	6-H	-CH_2_C_6_H_5_	24	62
5	**5e**	6-Cl	-CH_3_	12	85
6	**5f**	6-Cl	-C_2_H_5_	12	86
7	**5g**	6-Cl	-C_3_H_7_	12	82
8	**5h**	6-Cl	-CH_2_C_6_H_5_	24	67
9	**5i**	6-OCH_3_	-CH_3_	12	81
10	**5j**	6-OCH_3_	-C_2_H_5_	12	80
11	**5k**	6-OCH_3_	-C_3_H_7_	12	79
12	**5l**	6-OCH_3_	-CH_2_C_6_H_5_	24	60
13	**5m**	6-CH_3_	-CH_3_	12	81
14	**5n**	6-CH_3_	-C_2_H_5_	12	76
15	**5o**	6-CH_3_	-C_3_H_7_	12	76
16	**5p**	6-CH_3_	-CH_2_C_6_H_5_	24	64

^a^ Isolated yield after chromatographic purification.

### 2.2. Anti-TMV Activity

The antiviral activity of compound **5** against TMV was assayed by the reported method [9]. As it can be seen from the results presented in Table 3, some compounds possess good anti-TMV activity, such as the curative rates against TMV of compounds **5i** and **5m** which were 52.23% and 54.41%. These values are close to that of the the commercial control ningnanmycin (curative rate 55.27%).

**Table 3:** The *in vivo* antiviral activity towards TMV of the new compounds at 0.5 (mg/mL).

Entry	Compound	Protection Effect %	Curative Effect %	Inhibition Effect %
1	**5a**	57.95	48.27	39.72
2	**5b**	63.08	47.18	43.42
3	**5c**	62.11	43.83	33.29
4	**5d**	48.58	49.17	28.45
5	**5e**	58.67	46.28	33.50
6	**5f**	68.32	48.34	43.98
7	**5g**	56.76	48.27	46.77
8	**5h**	49.77	44.20	44.21
9	**5i**	59.79	52.23	40.98
10	**5j**	58.76	48.03	39.27
11	**5k**	40.20	46.73	41.04
12	**5l**	52.34	35.53	29.08
13	**5m**	68.75	54.41	46.88
14	**5n**	66.04	48.09	39.70
15	**5o**	58.97	47.93	42.33
16	**5p**	46.36	38.46	45.96
17	**Ningnanmycin**	82.03	55.27	52.16

## 3. Experimental

### 3.1. Instruments and Chemicals

Melting points were determined on a XT-4 binocular microscope (Beijing Tech Instrument Co., Beijing, China) and were not corrected. IR spectra were recorded on a Bruker VECTOR 22 spectrometer in KBr disks. ^1^H- and ^13^C-NMR spectral analyses (solvent CDCl_3_ or DMSO-*d_6_*) were performed on a JEOL-ECX 500 NMR spectrometer at room temperature using TMS as an internal standard. Elemental analyses were performed on an Elementar Vario-III CHN analyzer. MS spectra were recorded with a VG Autospec-3000 spectrometer. Analytical TLC was performed on silica gel GF254. Column chromatographic purification was carried out using silica gel GF254. Commercial reagents were used as received, unless otherwise indicated. Reactions were performed under a positive pressure of dry argon in oven-dried or flame-dried glassware equipped with a magnetic stir bar. Standard inert atmosphere techniques were used in handling all air and moisture sensitive reagents. All reagents were of analytical reagent grade or chemically pure. All solvents were dried, deoxygenated and redistilled before use.

### 3.2. Synthesis

#### 3.2.1. General Synthetic Methods for **3a**–**d**

To a magnetically stirred solution of 6-substituted benzothiazole (6.80 mmol) in toluene (5 mL) benzofuran-2-methanal (6.80 mmol) dissolved in toluene (5 mL) was added dropwise at room temperature. After attaching a Dean Stark trap, the reaction was allowed to reflux after adding acetic acid (0.5 mL). Complete consumption of starting materials was observed after 24 h. After recrystallization from ethanol the final compounds **3a**–**d** were isolated in good yields.

#### 3.2.2. Characterization of **3a**–**d**

*N-(Benzofuran-2-ylmethylene)benzo[d]thiazol-2-amine* (**3a**): yellow solid; mp:173–174 °C; yield: 77%; ^1^H-NMR (CDCl_3_) δ (ppm): 8.11 (d, 1H, *J* = 5 Hz, 11-CH), 7.98 (d, 1H, *J* = 5 Hz, 9-CH), 7.89 (d, 1H, *J* = 5 Hz, 6-CH), 7.77 (d, 1H, *J =* 10 Hz, 17-CH), 7.61–7.54 (m, 2H, 7-CH, 8-CH), 7.52 (s, 1H, 20-CH), 7.47–7.46 (m, 2H, 18-CH, 19-CH), 7.43-7.39 (m, 1H, 14-CH); ^13^C-NMR (DMSO-*d_6_*) δ (ppm): 171.5 (2-C), 156.4 (16-C), 154.8 (4-C), 151.8 (11-C), 135.1 (12-C), 129.8 (5-C), 129.4 (15-C), 128.0 (8-C), 127.4 (7-C), 125.9 (19-C), 124.7 (18-C), 123.9 (6-C), 123.3 (9-C), 120.2 (17-C), 113.0 (20-C), 112.6 (14-C); IR (KBr, cm^−1^) ν: 3049, 608, 1556, 1546, 1417, 1309, 1116, 954, 813, 750, 729; MS (ESI): *m/z* = 279 ([M+H]^+^), 301 ([M+Na]^+^).

*N-(Benzofuran-2-ylmethylene)-6-chlorobenzo[d]thiazol-2-amine* (**3b**): yellow solid; mp: 209–212 °C; yield: 76%; ^1^H-NMR (CDCl_3_) δ (ppm): 9.17 (s, 1H, 6-CH), 8.23 (s, 1H, 11-CH), 7.96 (s, 1H, 17-CH), 7.91 (d, 1H, *J* = 5 Hz, 9-CH), 7.84 (d, 1H, *J* = 5 Hz, 8-CH), 7.72 (s, 1H, 20-CH), 7.54–7.54 (m, 2H, 18-CH, 19-CH), 7.36 (s, 1H, 14-CH); ^13^C-NMR (DMSO-*d_6_*) δ (ppm): 172.5 (2-C), 156.5 (16-C), 155.3 (4-C), 152.2 (11-C), 150.6 (6-C), 136.5 (12-C), 130.2 (5-C), 129.5 (15-C), 129.2 (8-C), 127.8 (7-C), 124.7 (19-C), 124.4 (18-C), 123.9 (9-C), 122.7 (17-C), 120.6 (20-C), 112.7 (14-C); IR (KBr, cm^−1^) ν: 3086, 1598, 1539, 1425, 1330, 1294, 1166, 1122, 950, 812, 802, 738, 704, 605; MS (ESI): *m/z* = 313 ([M+H]^+^), 335 ([M+Na]^+^).

*N-(Benzofuran-2-ylmethylene)-6-methoxybenzo[d]thiazol-2-amine* (**3c**): yellow solid; mp: 147–150 °C; yield: 64%; ^1^H-NMR (CDCl_3_) δ (ppm): 9.09–9.07 (m, 1H, 11-CH), 7.88 (d, 1H, *J* = 5 Hz, 17-CH ), 7.81 (t, 2H, *J* = 15 Hz, 6-CH, 8-CH), 7.70 (t, 1H, *J =* 15 Hz, 9-CH), 7.62 (d, 1H, *J* = 5 Hz, 20-CH), 7.51 (d, 1H, *J* = 5 Hz, 19-CH), 7.34 (s, 1H, 14-CH), 7.09-7.07 (m, 1H, 18-CH), 3.81 (s, 3H, 22-C OCH_3_); ^13^C-NMR (DMSO-*d_6_*) δ (ppm): 168.9 (2-C), 157.9 (16-C), 156.3 (4-C), 153.5 (11-C), 152.4 (6-C), 146.1 (12-C), 136.5 (5-C), 129.1 (15-C), 128.0 (8-C), 124.6 (7-C), 124.0 (19-C), 123.7 (18-C), 119.4 (9-C), 116.6 (17-C), 112.5 (20-C), 105.6 (14-C), 56.2 (22-C); IR (KBr, cm^−1^) ν: 3093, 1598, 1556, 1541, 1485, 1452, 1429, 1263, 1226, 1120, 1056, 1024, 954, 908, 833, 817, 756, 609; MS (ESI): *m/z* = 309 ([M+H]^+^), 331 ([M+Na]^+^).

*N-(Benzofuran-2-ylmethylene)-6-methylbenzo[d]thiazol-2-amine* (**3d**): yellow solid; mp: 185–190 °C; yelid: 78%; ^1^H-NMR (CDCl_3_) δ (ppm): 8.37-8.36 (m, 2H, 11-CH, 6-CH), 8.05–8.00 (m, 2H, 9-CH, 17-CH), 7.92–7.90 (m, 2H, 18-CH, 19-CH), 7.51–7.47 (m, 2H, 14-CH, 20-CH), 7.46-7.40 (m, 1H, 8-CH), 2.43 (s, 3H, 21-CH_3_); ^13^C-NMR (DMSO-*d_6_*) δ (ppm): 181.4 (2-C), 170.5 (16-C), 166.2 (4-C), 154.3 (11-C), 129.8 (6-C), 129.3 (12-C), 128.8 (5-C), 126.9 (15-C), 124.7 (8-C), 123.8 (7-C), 122.9 (19-C), 122.5 (18-C), 121.3 (9-C), 120.0 (17-C), 117.9 (20-C), 112.9 (14-C), 21.7 (22-C); IR (KBr, cm^−1^) ν: 3028, 1602, 1579, 1562, 1473, 1433, 1361, 1305, 1213, 1186, 1120, 956, 916, 858, 825, 750, 740, 686, 609; MS (ESI): *m/z* = 293 ([M+H]^+^), 315 ([M+Na]^+^).

#### 3.2.3. General Synthetic Methods for **5a**–**p**

To a magnetically stirred solution of imines (0.50 mmol) in DCM (5 mL) malonic ester (0.7 mmol) was added dropwise at room temperature. The reaction was allowed to reach 35 °C, and complete consumption of starting materials was observed after 12–24 h. After removing the solvent by reduced pressure distillation, the mixture was subjected to column chromatography on silica gel (EA/PE = 1:7) to afford compounds **5a**–**p**.

*Dimethyl 2-((Benzo[d]thiazol-2-ylamino)(benzofuran-2-yl)methyl) malonate* (**5a**): white solid; mp: 70–72 °C; yield 82%; ^1^H-NMR (CDCl_3_) δ (ppm): 7.61–7.58 (m, 2H, 24-CH, 27-CH), 7.52–7.47 (m, 1H, 14-CH), 7.45–7.41 (m, 1H, 17-CH), 7.33–7.26 (m, 2H, 25-CH, 26-CH), 7.24–7.18 (m, 1H, 16-CH), 7.15-7.09 (m, 1H, 15-CH), 6.71 (s, 1H, NH), 6.75 (d, 1H, *J* = 15 Hz, 11-CH), 6.07 (d, 1H, *J* = 10 Hz, 8-CH), 4.37–4.33 (m, 1H, 2-CH), 3.76 (s, 6H, 28-CH_3_, 29-CH_3_); ^13^C-NMR (CDCl_3_) δ (ppm): 168.4 (20-C), 167.1 (1-C), 165.8 (3-C), 155.0 (10-C), 154.1 (13-C), 152.1 (22-C), 131.0 (23-C), 128.1 (12-C), 126.1 (15-C), 124.5 (16-C), 123.1 (25-C), 122.3 (26-C), 121.3 (24-C), 121.0 (27-C), 119.7 (14-C), 111.3 (17-C), 104.7 (11-C), 53.9 (8-C), 53.8 (2-C), 53.1 (28-C), 52.8 (29-C); MS (ESI): *m/z* = 411 ([M+H]^+^), 433 ([M+Na]^+^); MS (HREI): C_21_H_18_N_2_O_5_S Na for +, calculated 410.0940, found 410.0940; IR (KBr, cm^−1^) ν 3385, 2951, 1745, 1732, 1595, 1539, 1452, 1435, 1355, 1259, 1207, 1172, 1014, 966, 750, 725.

*Diethyl 2-((Benzo[d]thiazol-2-ylamino)(benzofuran-2-yl)methyl) malonate* (**5b**): white solid; mp: 110–112 °C; yield 80%; ^1^H-NMR (CDCl_3_) δ (ppm): 7.59 (d, 2H, *J* = 5 Hz, 24-CH, 27-CH), 7.50 (s, 1H, 14-CH), 7.43 (s, 1H, 17-CH), 7.31–7.26 (m, 3H, 25-CH, 26-CH, 16-CH), 7.20 (s, 1H, 15-CH), 7.12 (s, 1H, NH), 6.75 (s, 1H, 11-CH), 6.08 (s, 1H, 8-CH), 4.33 (d, 1H, *J* = 5 Hz, 2-CH), 4.25–4.17 (m, 4H, 28-CH_2_, 30-CH_2_ ), 1.21 (s, 6H, 29-CH_3_, 31-CH_3_); ^13^C-NMR (CDCl_3_) δ (ppm): 168.1 (20-C), 166.7 (1-C), 165.9 (3-C), 155.0 (10-C), 154.3 (13-C), 152.1 (22-C), 131.0 (23-C), 128.1 (12-C), 126.0 (15-C), 124.5 (16-C), 123.1 (25-C), 122.2 (26-C), 121.3 (24-C), 120.9 (27-C), 119.6 (14-C), 111.2 (17-C), 104.7 (11-C), 62.5 (28-C), 62.1 (30-C), 54.1 (8-C), 52.8 (2-C), 14.1 (29-C), 14.0 (31-C); MS (ESI): *m/z* = 439 ([M+H]^+^), 461 ([M+Na]^+^); MS (HREI): C_23_H_22_N_2_O_5_S Na for +, calculated 438.1249, found 438.1253; IR (KBr, cm^−1^) ν 3369, 1739, 1718, 1537, 1485, 1454, 1286, 1242, 1201, 1184, 1176, 1018, 947, 802, 758.

*Dipropyl 2-((Benzo[d]thiazol-2-ylamino)(benzofuran-2-yl)methyl) malonate* (**5c**): white solid; mp: 127–129 °C; yield 78%; ^1^H-NMR (CDCl_3_) δ (ppm): 7.59–7.55 (m, 2H, 24-CH, 27-CH), 7.48 (d, 1H, *J* = 5 Hz, 14-CH), 7.42 (d, 1H, *J* = 5 Hz, 17-CH), 7.30–7.23 (m, 2H, 25-CH, 26-CH), 7.19 (t, 1H, *J* = 5 Hz, 16-CH), 7.09 (t, 1H, *J* = 5 Hz, 15-CH), 6.73 (d, 1H, *J* = 5 Hz, 11-CH), 6.07 (s, 1H, NH), 4.35–4.32 (m, 1H, 8-CH), 4.13-4.09 (m, 4H, 28-CH_2_, 29- CH_2_), 3.40 (d, 1H, *J* = 5 Hz, 2-CH), 1.61–1.56 (m, 4H, 30-CH_2_, 31-CH_2_), 0.87-0.82 (m, 6H, 32-CH_3_, 33-CH_3_); ^13^C-NMR (CDCl_3_) δ (ppm): 168.3 (20-C), 166.8 (1-C), 165.7 (3-C), 155.0 (10-C), 154.4 (13-C), 152.2 (22-C), 131.0 (23-C), 128.1 (12-C), 126.0, (15-C) 124.4 (16-C), 123.1 (25-C), 122.2 (26-C), 121.3 (24-C), 120.9 (27-C), 119.7 (14-C), 111.2 (17-C), 104.6 (11-C), 68.0 (28-C), 67.7 (29-C), 54.0 (8-C), 52.8 (2-C), 21.9 (30-C), 21.8 (31-C), 10.3 (32-C), 10.2 (33-C); MS (ESI): *m/z* = 467 ([M+H]^+^), 489 ([M+Na]^+^); MS (HREI): C_25_H_26_N_2_O_5_S Na for +, calculated 438.1249, found 466.1550; IR (KBr, cm^−1^) ν 3356, 2962, 1751, 1724, 1600, 1564, 1548, 1454, 1442, 1386, 1313, 1271, 1176, 1136, 1053, 925, 815, 759, 754.

*Dibenzyl 2-((Benzo[d]thiazol-2-ylamino)(benzofuran-2-yl)methyl) malonate* (**5d**): white solid; mp: 110–112 °C; yield 62%; ^1^H-NMR (CDCl_3_) δ (ppm): 7.57 (d, 1H, *J* = 10 Hz, 24-CH), 7.54 (d, 1H, *J =* 10 Hz, 27-CH), 7.44 (d, 1H, *J* = 10 Hz, 14-CH), 7.34–7.32 (m, 10H, 31-CH, 32-CH, 33-CH, 34-CH, 35-CH, 37-CH, 38-CH, 39-CH, 40-CH, 41-CH), 7.25 (s, 1H, NH), 7.19–7.18 (m, 1H, 17-CH), 7.16–7.15 (m, 2H, 25-CH, 26-CH), 7.13–7.11 (m, 2H, 15-CH, 16-CH), 6.65 (s, 1H, 11-CH), 5.17 (s, 4H, 28-CH_2_, 29-CH_2_), 4.44 (d, 1H, *J* = 5 Hz, 8-CH), 3.48 (s, 1H, 2-CH); ^13^C-NMR (CDCl_3_) δ (ppm): 167.9 (20-C), 166.4 (1-C), 166.3 (3-C), 165.5 (10-C), 155.0 (13-C), 154.1 (22-C), 152.1 (30-C), 135.3 (36-C), 134.8 (23-C), 134.7 (32-C), 131.1 (34-C), 128.7 (38-C), 128.6 (40-C), 128.5 (12-C), 128.5 (31-C), 128.4 (35-C), 128.3 (37-C), 128.2 (41-C), 128.1 (39-C), 126.0 (33-C), 124.5 (15-C), 123.1 (26-C), 122.2 (25-C), 121.3 (16-C), 120.9 (24-C), 119.7 (17-C), 111.3 (27-C), 104.7 (14-C), 67.4 (11-C), 54.1 (28-C), 54.0 (29-C), 52.6 (8-C), 41.7 (2-C); MS (ESI): *m/z* = 563 ([M+H]^+^), 585 ([M+Na]^+^); MS (HREI): C_33_H_26_N_2_O_5_S Na for +, calculated 562.1562, found 562.1569; IR (KBr, cm^−1^) ν 3352, 2922, 1747, 1716, 1537, 1454, 1444, 1348, 1249, 1172, 954, 750, 727.

*Dimethyl 2-(Benzofuran-2-yl((6-chlorobenzo[d]thiazol-2-yl)amino)methyl) malonate* (**5e**): white solid; mp: 115–117 °C; yield 85%; ^1^H-NMR (CDCl_3_) δ (ppm): 7.53 (s, 1H, 24-CH), 7.49 (d, 1H, *J* = 5 Hz, 27-CH), 7.46 (d, 1H, *J* = 5 Hz, 14-CH), 7.42 (d, 1H, *J* = 5 Hz, 17-CH), 7.26 (d, 1H, *J* = 5 Hz, 25-CH), 7.22–7.17 (m, 2H, 26-CH, 15-CH), 6.73 (d, 1H, *J* = 5 Hz, 11-CH), 6.03 (s, 1H, NH), 5.29 (d, 1H, *J* = 5 Hz, 8-CH), 4.32-4.31 (m, 1H, 2-CH), 3.73 (s, 6H, 29-CH_3_, 30-CH_3_); ^13^C-NMR (CDCl_3_) δ (ppm): 168.4 (20-C), 167.0 (1-C), 165.9 (3-C), 155.0 (10-C), 153.8 (13-C), 150.8 (22-C), 132.2 (23-C), 128.0 (12-C), 127.4 (15-C), 126.5 (16-C), 124.6 (25-C), 123.2 (26-C), 121.4 (24-C), 120.6 (27-C), 120.3 (14-C), 111.3 (17-C), 104.7 (11-C), 53.7 (8-C), 53.4 (2-C), 53.1 (29-C), 52.7 (30-C); MS (ESI): *m/z* = 445 ([M+H]^+^), 467 ([M+Na]^+^); MS (HREI): C_21_H_17_ClN_2_O_5_S Na for +, calculated 444.0547, found 444.0547; IR (KBr, cm^−1^) ν 3340, 2954, 1745, 1593, 1537, 1483, 1436, 1359, 1226, 1161, 1138, 1037, 974, 954, 875, 812, 759.

*Diethyl 2-(Benzofuran-2-yl((6-chlorobenzo[d]thiazol-2-yl)amino)methyl) malonate* (**5f**): white solid; mp: 92–94 °C; yield 86%; ^1^H-NMR (CDCl_3_) δ (ppm): 7.53 (s, 1H, 24-CH), 7.49 (d, 1H, *J* = 5 Hz, 27-CH), 7.45 (d, 1H, *J* = 5 Hz, 14-CH), 7.42 (d, 1H, *J* = 5 Hz, 17-CH), 7.26 (d, 1H, *J* = 5 Hz, 26-CH), 7.22–7.17 (m, 2H, 15-CH, 16-CH), 6.94 (s, 1H, 11-CH), 6.72 (s, 1H, 8-CH), 6.05 (s, 1H, NH), 4.28 (d, 1H,*J* = 5 Hz, 2-CH), 4.24–4.13 (m, 4H, 28-CH_2_, 30-CH_2_), 1.21–1.16 (m, 6H, 29-CH_3_, 31-CH_3_); ^13^C-NMR (CDCl_3_) δ (ppm): 168.1 (20-C), 166.6 (1-C), 165.9 (3-C), 155.0 (10-C), 154.1 (13-C), 150.8 (22-C), 132.2 (23-C), 128.0 (12-C), 127.3 (15-C), 126.5 (16-C), 124.5 (25-C), 123.1 (26-C), 121.3 (24-C), 120.6 (27-C), 120.3 (14-C), 111.3 (17-C), 104.7 (11-C), 62.5 (28-C), 62.2 (30-C), 54.0 (8-C), 52.7 (2-C), 14.1 (29-C), 14.0 (31-C); MS (ESI): *m/z* = 473 ([M+H]^+^), 495 ([M+Na]^+^); MS (HREI): C_23_H_21_ClN_2_O_5_S Na for +, calculated 472.0860, found 472.0850; IR (KBr, cm^−1^) ν 3346, 2974, 1741, 1718, 1537, 1483, 1396, 1367, 1301, 1242, 1172, 1029, 974, 812, 763.

*Dipropyl 2-(Benzofuran-2-yl((6-chlorobenzo[d]thiazol-2-yl)amino)methyl) malonate* (**5g**): white solid; mp: 88–90 °C; yield 82%; ^1^H-NMR (CDCl_3_) δ (ppm): 7.54 (s, 1H, 24-CH), 7.49 (d, 1H, *J* = 5 Hz, 27-CH), 7.45 (d, 1H,*J* = 5 Hz, 14-CH), 7.42 (d, 1H, *J* = 5 Hz, 17-CH), 7.27–7.22 (m, 2H, 25-CH, 26-CH), 7.20-7.17 (m, 1H, 15-CH), 6.93 (s, 1H, 11-CH), 6.72 (s, 1H, 8-CH), 6.05 (s, 1H, NH), 4.31 (d, 1H, *J* = 5 Hz, 2-CH), 4.14-4.06 (m, 4H, 29-CH_2_, 30-CH_2_), 1.61–1.57 (m, 4H, 31-CH_2_, 32-CH_2_), 0.87–0.83 (m, 6H, 33-CH_3_, 34-CH_3_); ^13^C-NMR (CDCl_3_) δ (ppm): 168.3 (20-C), 166.7 (1-C), 165.8 (3-C), 155.0 (10-C), 154.1 (13-C), 150.8 (22-C), 132.2 (23-C), 128.1 (12-C), 127.3 (15-C), 126.5 (16-C), 124.5 (25-C), 123.1 (26-C), 121.3 (24-C), 120.6 (27-C), 120.3 (14-C), 111.3 (17-C), 104.7 (11-C), 68.0 (29-C), 67.7 (30-C), 54.0 (8-C), 52.7 (2-C), 21.9 (31-C), 21.8 (32-C), 10.4 (33-C), 10.3 (34-C); MS (ESI): *m/z* = 501 ([M+H]^+^), 523 ([M+Na]^+^); MS (HREI): C_25_H_25_ClN_2_O_5_S Na for +, calculated 500.1173, found 500.1171; IR (KBr, cm^−1^) ν 3357, 2966, 1747, 1720, 1593, 1533, 1483, 1446, 1392, 1354, 1290, 1238, 1197, 1172, 1053, 945, 812, 759.

*Dibenzyl 2-(Benzofuran-2-yl((6-chlorobenzo[d]thiazol-2-yl)amino)methyl) malonate* (**5h**): white solid; mp: 116–118 °C; yield 67%; ^1^H-NMR (CDCl_3_) δ (ppm): 7.53 (s, 1H, 24-CH), 7.45 (d, 1H, *J* = 10 Hz, 27-CH), 7.42 (d, 1H, *J* = 5 Hz, 14-CH), 7.36–7.66 (m, 7H, 31-CH, 32-CH, 33-CH, 34-CH, 35-CH, 37-CH, 38-CH), 7.26–7.24 (m, 2H, 39-CH, 40-CH), 7.19–7.18 (m, 2H, 17-CH, 41-CH), 7.17–7.15 (m, 2H, 15-CH, 26-CH), 7.13–7.11 (m, 2H, 11-CH, 26-CH), 6.81 (s, 1H, NH), 6.11 (s, 1H, 8-CH), 5.17–5.14 (m, 4H, 28-CH_2_, 29-CH_2_), 3.47 (s, 1H, 2-CH); ^13^C-NMR (CDCl_3_) δ (ppm): 168.0 (20-C), 166.4 (1-C), 165.6 (3-C), 155.0 (10-C), 153.8 (13-C), 150.8 (22-C), 135.3 (30-C), 134.7 (36-C), 134.6 (23-C), 132.3 (32-C), 128.7 (34-C), 128.6 (38-C), 128.6 (40-C), 128.5 (12-C), 128.5 (31-C), 128.5 (35-C), 128.4 (37-C), 128.4 (41-C), 128.2 (39-C), 128.0 (33-C), 127.4 (15-C), 126.5 (26-C), 124.6 (25-C), 123.2 (16-C), 121.4 (24-C), 120.5 (17-C), 120.4 (27-C), 111.3 (14-C), 104.7 (11-C), 68.2 (28-C), 67.8 (29-C), 54.0 (8-C), 41.9 (2-C); MS (ESI): *m/z* = 597 ([M+H]^+^), 619 ([M+Na]^+^); MS (HREI): C_33_H_25_ClN_2_O_5_S Na for +, calculated 596.1173, found 596.1189; IR (KBr, cm^−1^) ν 3388, 2966, 1745, 1730, 1599, 1543, 1529, 1487, 1454, 1381, 1259, 1220, 1147, 1004, 817, 748, 694.

*Dimethyl 2-(Benzofuran-2-yl((6-methoxybenzo[d]thiazol-2-yl)amino)methyl) malonate* (**5i**): white solid; mp: 85–87 °C; yield 81%; ^1^H-NMR (CDCl_3_) δ (ppm): 7.49–7.45 (m, 2H, 24-CH, 27-CH), 7.42 (d, 1H, *J* = 5 Hz, 14-CH), 7.27–7.23 (m, 1H, 17-CH), 7.20–7.17 (m, 1H, 25-CH), 7.11 (s, 1H, 26-CH), 6.89 (d, 1H, *J* = 5 Hz, 15-CH), 6.73 (d, 1H, *J* = 5 Hz, 11-CH ), 6.64 (s, 1H, NH), 6.01 (s, 1H, 8-CH), 4.34–4.32 (m, 1H, 2-CH), 3.80 (s, 3H, 29-C-OCH_3_), 3.73 (s, 6H, 30-CH_3_, 31-CH_3_); ^13^C-NMR (CDCl_3_) δ (ppm): 168.4 (20-C), 167.1 (1-C), 164.1 (3-C), 155.6 (10-C), 154.9 (13-C), 154.2 (22-C), 146.3 (23-C), 132.0 (12-C), 128.1 (15-C), 124.5 (16-C), 123.1 (25-C), 121.3 (26-C), 120.0 (24-C), 113.7 (27-C), 111.3 (14-C), 105.3 (17-C), 104.7 (11-C), 56.1 (8-C), 53.9 (2-C), 53.3 (29-C), 53.1 (30-C), 52.8 (31-C); MS (ESI): *m/z* = 441 ([M+H]^+^), 463 ([M+Na]^+^); MS (HREI): C_22_H_20_N_2_O_6_S Na for +, calculated 440.1042, found 440.1047; IR (KBr, cm^−1^) ν 3377, 2953, 1745, 1724, 1604, 1544, 1483, 1471, 1454, 1436, 1359, 1247, 1170, 1058, 1029, 968, 815, 759.

*Diethyl 2-(benzofuran-2-yl((6-methoxybenzo[d]thiazol-2-yl)amino)methyl) malonate* (**5j**): white solid; mp: 85–88 °C; yield 80%; ^1^H-NMR (CDCl_3_): δ (ppm) 7.50–7.45 (m, 2H, 24-CH, 27-CH), 7.42 (d, 1H, *J* = 5 Hz, 14-CH), 7.26–7.23 (m, 1H, 17-CH), 7.20–7.17 (m, 1H, 26-CH), 7.11 (s, 1H, 15-CH), 6.90–6.87 (m, 1H, 16-CH), 6.72 (s, 1H, 11-CH), 6.70 (s, 1H, NH), 6.02 (s, 1H, 8-CH), 4.29 (d, 1H, *J* = 5 Hz, 2-CH), 4.24–4.14 (m, 4H, 28-CH_2_, 30-CH_2_), 3.80 (s, 3H, 25-C-OCH_3_), 1.21–1.17 (m, 6H, 29-CH_3_, 31-CH_3_); ^13^C-NMR (CDCl_3_) δ (ppm): 168.1 (20-C), 166.7 (1-C), 164.1 (3-C), 155.5 (10-C), 154.9 (13-C), 154.5 (22-C), 146.3 (23-C), 131.9 (12-C), 128.1 (15-C), 124.4 (16-C), 123.1 (25-C), 121.3 (26-C), 120.0 (24-C), 113.7 (27-C), 111.2 (14-C), 105.3 (17-C), 104.6 (11-C), 62.4 (28-C), 62.1 (30-C), 55.9 (8-C), 54.1 (25-C), 52.7 (2-C), 14.1 (29-C), 14.0 (31-C); MS (ESI): *m/z* = 469 ([M+H]^+^), 491 ([M+Na]^+^); MS (HREI): C_24_H_24_N_2_O_6_S Na for +, calculated 468.1355, found 468.1336; IR (KBr, cm^−1^) ν 3377, 2974, 1745, 1712, 1602, 1543, 1490, 1469, 1436, 1280, 1188, 1033, 981, 855, 825, 756.

*Dipropyl 2-(Benzofuran-2-yl((6-methoxybenzo[d]thiazol-2-yl)amino)methyl) malonate* (**5k**): white solid; mp: 67–70 °C; yield 79%; ^1^H-NMR (CDCl_3_) δ (ppm): 7.49–7.45 (m, 3H, 24-CH, 27-CH, 14-CH), 7.25–7.06 (m, 3H, 17-CH, 25-CH, 26-CH), 6.89-6.81 (m, 1H, 15-CH), 6.72 (s, 1H, 11-CH), 6.62 (s, 1H, NH), 5.98 (s, 1H, 8-CH), 4.31–4.26 (m, 1H, 2-CH), 4.08–4.01 (m, 4H, 29-CH_2_, 30-CH_2_), 3.80 (s, 3H, 28-C-OCH_3_), 1.60-1.52 (m, 4H, 31-CH_2_, 32-CH_2_), 0.87–0.77 (m, 6H, 33-CH_3_, 34-CH_3_); ^13^C-NMR (CDCl_3_) δ (ppm): 168.2 (20-C), 166.8 (1-C), 164.0 (3-C), 155.5 (10-C), 155.0 (13-C), 154.5 (22-C), 146.3 (23-C), 132.0 (12-C), 128.2 (15-C), 124.4 (16-C), 123.0 (25-C), 121.2 (26-C), 120.0 (24-C), 113.6 (27-C), 111.2 (14-C), 105.3 (17-C), 104.6 (11-C), 67.9 (29-C), 67.6 (30-C), 56.0 (8-C), 54.1 (2-C), 52.7 (28-C), 21.8 (31-C), 21.7 (32-C), 10.3 (33-C), 10.2 (34-C); MS (ESI): *m/z* = 497 ([M+H]^+^), 519 ([M+Na]^+^); MS (HREI): C_26_H_28_N_2_O_6_S Na for +, calculated 496.1668, found 496.1669; IR (KBr, cm^−1^) ν 3348, 2951, 1741, 1716, 1604, 1543, 1483, 1357, 1288, 1172, 1064, 947, 850, 806, 759.

*Dibenzyl 2-(Benzofuran-2-yl((6-methoxybenzo[d]thiazol-2-yl)amino)methyl) malonate* (**5l**): white solid; mp: 110–112 °C; yield 60%; ^1^H-NMR (CDCl_3_): δ (ppm) 7.42 (d, 2H, *J* = 5 Hz, 27-CH, 24-CH), 7.35 (d, 1H, *J* = 5 Hz, 14-CH), 7.27–7.23 (m, 14H, 31-CH, 32-CH, 33-CH, 34-CH, 35-CH, 37-CH, 38-CH, 39-CH, 40-CH, 17-CH, 41-CH, 15-CH, 26-CH, 11-CH), 6.90–6.88 (m, 1H, 26-CH), 6.64 (s, 1H, NH), 6.01 (s, 1H, 8-CH), 5.19-5.06 (m, 4H, 28-CH_2_, 29-CH_2_), 4.43 (d, 1H, *J* = 5 Hz, 2-CH), 3.80 (s, 3H, 25-C-OCH_3_); ^13^C-NMR (CDCl_3_) δ (ppm): 167.9 (20-C), 166.5 (1-C), 163.8 (3-C), 155.6 (10-C), 155.0 (13-C), 154.2 (22-C), 146.3 (30-C), 134.8 (36-C), 134.7 (23-C), 132.1 (32-C), 128.7 (34-C), 128.6 (38-C), 128.5 (40-C), 128.5 (12-C), 128.5 (31-C), 128.4 (35-C), 128.4 (37-C), 128.3 (41-C), 128.2 (39-C), 128.1 (33-C), 124.4 (15-C), 123.1 (26-C), 121.3 (25-C), 120.1 (16-C), 113.7 (24-C), 111.3 (17-C), 105.3 (27-C), 104.7 (14-C), 68.1 (11-C), 67.8, 67.8 (28-C, 29-C), 56.0 (25-C), 54.2 (8-C), 52.6 (2-C); MS (ESI): *m/z* = 593 ([M+H]^+^), 615 ([M+Na]^+^); MS (HREI): C_34_H_28_N_2_O_6_S Na for +, calculated 592.1668, found 592.1687; IR (KBr, cm^−1^) ν 3350, 2954, 1747, 1716, 1602, 1543, 1469, 1454, 1348, 1222, 1172, 1028, 954, 823, 750, 696.

*Dimethyl 2-(Benzofuran-2-yl((6-methylbenzo[d]thiazol-2-yl)amino)methyl) malonate* (**5m**): white solid; mp: 108–109 °C; yield 81%; ^1^H-NMR (CDCl_3_) δ (ppm): 7.48 (d, 1H, *J* = 5 Hz, 24-CH), 7.45 (d, 1H, *J* = 5 Hz, 27-CH), 7.42 (d, 1H, *J* = 5 Hz, 14-CH), 7.38 (s, 1H, 17-CH), 7.25 (t, 1H, *J* = 15 Hz, 25-CH), 7.29 (t, 1H, *J =* 15 Hz, 26-CH), 7.10 (d, 1H, *J* = 5 Hz*,* 15-CH), 6.72 (s, 1H, 11-CH), 6.71 (s, 1H, NH ), 6.01 (s, 1H, 8-CH), 4.43 (d, 1H, *J* = 5 Hz, 2-CH), 3.73 (s, 6H, 29-CH_3_, 30-CH_3_), 2.38 (s, 3H, 28-CH_3_); ^13^C-NMR (CDCl_3_) δ (ppm): 168.4 (20-C), 167.0 (1-C), 165.1 (3-C), 155.0 (10-C), 154.2 (13-C), 150.0 (22-C), 132.1 (23-C), 131.0 (12-C), 128.1 (15-C), 127.2 (16-C), 124.5 (25-C), 123.1 (26-C), 121.3 (24-C), 121.0 (27-C), 119.3 (14-C), 111.3 (17-C), 104.7 (11-C), 53.8 (8-C), 53.3 (2-C), 53.0 (29-C), 52.8 (30-C), 21.3 (28-C); MS (ESI): *m/z* = 425 ([M+H]^+^), 447 ([M+Na]^+^); MS (HREI): C_22_H_20_N_2_O_5_S Na for +, calculated 424.1093, found 424.1097; IR (KBr, cm^−1^) ν 3361, 2954, 1743, 1724, 1539, 1487, 1454, 1435, 1357, 1224, 1172, 1147, 1033, 972, 817, 759.

*Diethyl 2-(Benzofuran-2-yl((6-methylbenzo[d]thiazol-2-yl)amino)methyl) malonate* (**5n**): white solid; mp: 105–107 °C; yield 76%; ^1^H-NMR (CDCl_3_) δ (ppm): 7.48-7.47 (d, 1H, *J* = 5 Hz, 24-CH), 7.45 (d, 1H, *J* = 5 Hz, 27-CH), 7.42 (d, 1H, *J* = 5 Hz, 14-CH), 7.38 (s, 1H, 17-CH), 7.25 (t, 1H, *J =* 15 Hz, 26-CH), 7.20–7.17 (m, 1H, 15-CH), 7.10 (d, 1H, *J* = 5 Hz, 16-CH), 6.74 (s, 1H, NH), 6.72 (s, 1H, 11-CH), 6.03 (s, 1H, 8-CH), 4.29 (m, 1H, 2-CH), 4.24–4.14 (m, 4H, 28-CH_2_, 30-CH_2_), 2.38 (s, 3H, 25-CH_3_), 1.21–1.16 (m, 6H, 29-CH_3_, 31-CH_3_); ^13^C-NMR (CDCl_3_) δ (ppm): 168.1 (20-C), 165.1 (1-C), 155.0 (3-C), 154.5 (10-C), 150.0 (13-C), 133.7 (22-C), 132.0 (23-C), 131.0 (12-C), 128.1 (15-C), 127.2 (16-C), 124.4 (25-C), 123.0 (26-C), 121.3 (24-C), 121.0 (27-C), 119.2 (14-C), 111.2 (17-C), 104.6 (11-C), 62.4 (28-C), 62.1 (30-C), 54.1 (8-C), 52.8 (2-C), 21.3 (25-C), 14.1 (29-C), 14.0 (31-C); MS (ESI): *m/z* = 453 ([M+H]^+^), 475 ([M+Na]^+^); MS (HREI): C_24_H_24_N_2_O_5_S Na for +, calculated 452.1406, found 452.1407; IR (KBr, cm^−1^) ν 3352, 2976, 1743, 1720, 1600, 1560, 1541, 1487, 1456, 1336, 1301, 1238, 1172, 1029, 939, 858, 810, 763.

*Dipropyl 2-(Benzofuran-2-yl((6-methylbenzo[d]thiazol-2-yl)amino)methyl) malonate* (**5o**): white solid; mp: 76–78 °C; yield 73%; ^1^H-NMR (CDCl_3_) δ (ppm): 7.48–7.43 (m, 3H, 24-CH, 27-CH, 14-CH), 7.41 (d, 1H, *J* = 10 Hz, 17-CH), 7.38 (s, 1H, 25-CH), 7.25–7.23 (m, 1H, 26-CH), 7.10–7.08 (m, 1H, 15-CH), 6.80 (s, 1H, 11-CH), 6.71 (s, 1H, NH), 6.04 (s, 1H, 8-CH), 4.33-4.32 (m, 1H, 2-CH), 4.13–4.06 (m, 4H, 29-CH_2_, 30-CH_2_), 2.38 (s, 3H, 28-CH_3_), 1.61-1.56 (m, 4H, 31-CH_2_, 32-CH_2_), 0.87–0.82 (m, 6H, 33-CH_3_, 34-CH_3_); ^13^C-NMR (CDCl_3_) δ (ppm): 168.2 (20-C), 166.8 (1-C), 165.1 (3-C), 155.0 (10-C), 154.5 (13-C), 150.0 (22-C), 132.0 (23-C), 131.0 (12-C), 128.1 (15-C), 127.2 (16-C), 124.4 (25-C), 123.0 (26-C), 121.3 (24-C), 121.0 (27-C), 119.2 (14-C), 111.2 (17-C), 104.6 (11-C), 68.0 (29-C), 67.6 (30-C), 54.1 (8-C), 52.8 (2-C), 21.9 (28-C), 21.8 (31-C), 21.3 (32-C), 10.3 (33-C), 10.2 (34-C); MS (ESI): *m/z* = 481([M+H]^+^), 503 ([M+Na]^+^); MS (HREI): C_26_H_28_N_2_O_5_S Na for +, calculated 480.1719, found 480.1711; IR (KBr, cm^−1^) ν 3365, 2966, 1747, 1722, 1535, 1483, 1456, 1354, 1290, 1172, 1055, 954, 808, 761.

*Dibenzyl 2-(Benzofuran-2-yl((6-methylbenzo[d]thiazol-2-yl)amino)methyl) malonate* (**5p**): white solid; mp: 126–128 °C; yield 64%; ^1^H-NMR (CDCl_3_) δ (ppm): 7.43–7.41 (m, 2H, 27-CH, 24-CH), 7.35–7.31 (m, 3H, 14-CH, 31-CH, 32-CH), 7.23–7.14 (m, 10H, 33-CH, 34-CH, 35-CH, 37-CH, 38-CH, 39-CH, 40-CH, 17-CH, , 41-CH, 15-CH), 7.12–7.08 (m, 3H, 26-CH, 11-CH, 26-CH), 6.64–6.62 (m, 1H, NH), 6.10 (s, 1H, 8-CH), 5.17–5.04 (m, 4H, 28-CH_2_, 29-CH_2_), 4.43-4.41 (m, 1H, 2-CH), 2.39–2.37 (s, 3H, 25-CH_3_); ^13^C-NMR (CDCl_3_) δ (ppm): 167.9 (20-C), 166.5 (1-C), 165.0 (3-C), 155.0 (10-C), 154.2 (13-C), 150.0 (22-C), 134.8 (30-C), 134.7 (36-C), 132.0 (23-C), 131.1 (32-C), 128.7 (34-C), 128.7 (38-C), 128.6 (40-C), 128.6 (12-C), 128.5 (31-C), 128.5 (35-C), 128.2 (37-C), 128.2 (41-C), 128.1 (39-C), 128.1 (33-C), 127.2 (15-C), 127.2 (26-C), 124.7 (25-C), 123.1 (16-C), 121.4 (24-C), 121.0 (17-C), 119.3 (27-C), 111.3 (14-C), 104.7 (11-C), 68.1 (28-C), 67.8 (29-C), 54.1 (8-C), 52.7 (2-C), 21.4 (25-C); MS (ESI): *m/z* = 577 ([M+H]^+^), 599 ([M+Na]^+^); MS (HREI): C_34_H_28_N_2_O_5_S Na for +, calculated 576.1719, found 576.1702; IR (KBr, cm^−1^) ν 3348, 2954, 1743, 1724, 1537, 1487, 1452, 1382, 1238, 1170, 1012, 910, 808, 731, 702.

### 3.3. Anti-TMV Activity Section

#### 3.3.1. Purification of Tobacco Mosaic Virus

Using Gooding’s method [21], the upper leaves of *Nicotiana tabacum* inoculated with TMV were selected and ground in phosphate buffer and then filtered through double-layer pledget. The filtrate was centrifuged at 10,000 *g*, treated with PEG twice, and centrifuged again. The whole experiment was processed at 4 °C. Absorbance value was estimated at 260 nm by ultraviolet spectrophotometer:

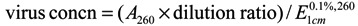
(1)


#### 3.3.2. Inhibition Effect of Compound on TMV *in Vivo* [22]

The virus was inhibited by mingling with the compound solution at the same volume for 30 min. The mixture was then inoculated on the left side of the leaves of *N. tabacum L.*, whereas the right side of the leaves was inoculated with the mixture of solvent and the virus for control. The local lesion numbers were recorded 3–4 days after inoculation [10]. Three repetitions were conducted for each compound.

#### 3.3.3. Curative Effect of Compounds on TMV *in Vivo* [22]

The leaves of *N. tabacum L*. growing at the same ages were selected. TMV at a concentration of 6 × 10^−3^ mg/mL was dipped and inoculated on the whole leaves. Then the leaves were washed with water and dried. The compound solution was smeared on the left side, and the solvent was smeared on the right side for control. The local lesion numbers were then recorded 3–4 days after inoculation. For each compound, three repetitions were conducted to ensure the reliability of the results. 

#### 3.3.4. Protective Effect of Compounds on TMV *in Vivo* [22]

The leaves of *N. tabacum L.* growing at the same ages were selected. The compound solution was smeared on the left side for 12 h and the solvent was smeared on the right side for control. The TMV at a concentration of 6 × 10^−3^ mg/mL was inoculated on the whole leaves. The local lesion numbers were then recorded 3–4 days after inoculation. For each compound, three repetitions were conducted to ensure the reliability of the results:

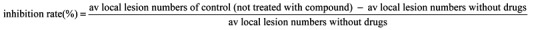



## 4. Conclusions

Sixteen novel β-amino ester derivatives containing benzofuran and benzothiazole units were designed and synthesized seeking anti-TMV activity. The bioassays identified these new compounds as possessing weak to good antiviral activities. The bioassay test results showed that compounds **5i** and **5m** have good antiviral activity against TMV, with curative rates of 52.23% and 54.41% at a concentration of 0.5 mg/mL.

## Supplementary Materials

Supplementary materials can be accessed at: http://www.mdpi.com/1420-3049/18/11/13623/s1.
