# Supplementary material for: Synthesis and Anti-TMV Activity of Dialkyl/dibenzyl 2-((6-Substituted-benzo[d]thiazol-2-ylamino)(benzofuran-2-yl)methyl) Malonates
Source: Molecules. 2013 Nov 4;18(11):13623–35. doi: 10.3390/molecules181113623 (PMC6270053; doi:10.3390/molecules181113623)

Supplementary Materials

Dimethyl-2-((benzo[d]thiazol-2-ylamino)(benzofuran-2-yl)methyl) (**5a**)

Figure S1. The High Resolution Mass Spectra of **5a**.

Monoisotopic Mass, Odd and Even Electron Ions
16 formula(e) evaluated with 1 results within limits (up to 51 closest results for each mass)
Elements Used:
C: 0-200 H: 0-400 N: 2-2 O: 4-6 S: 1-1

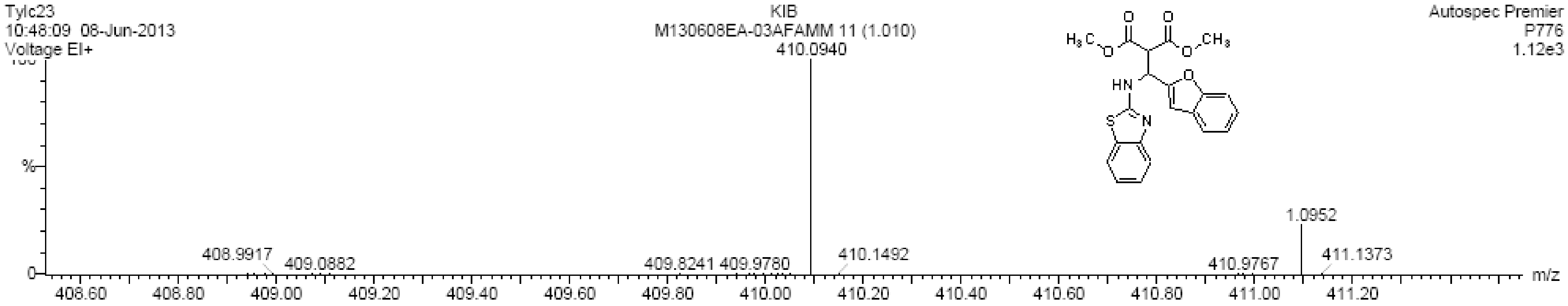

Minimum:				-10.0		
Maximum:		80.0	10.0	120.0		
Mass	Calc. Mass	mDa	PPM	DBE	i-FIT	Formula
410.0940	410.0936	0.4	1.0	14.0	2773598.5	C21 H18 N2 O5 S

Figure S2. The ^{1}H-NMR Spectra of **5a**.

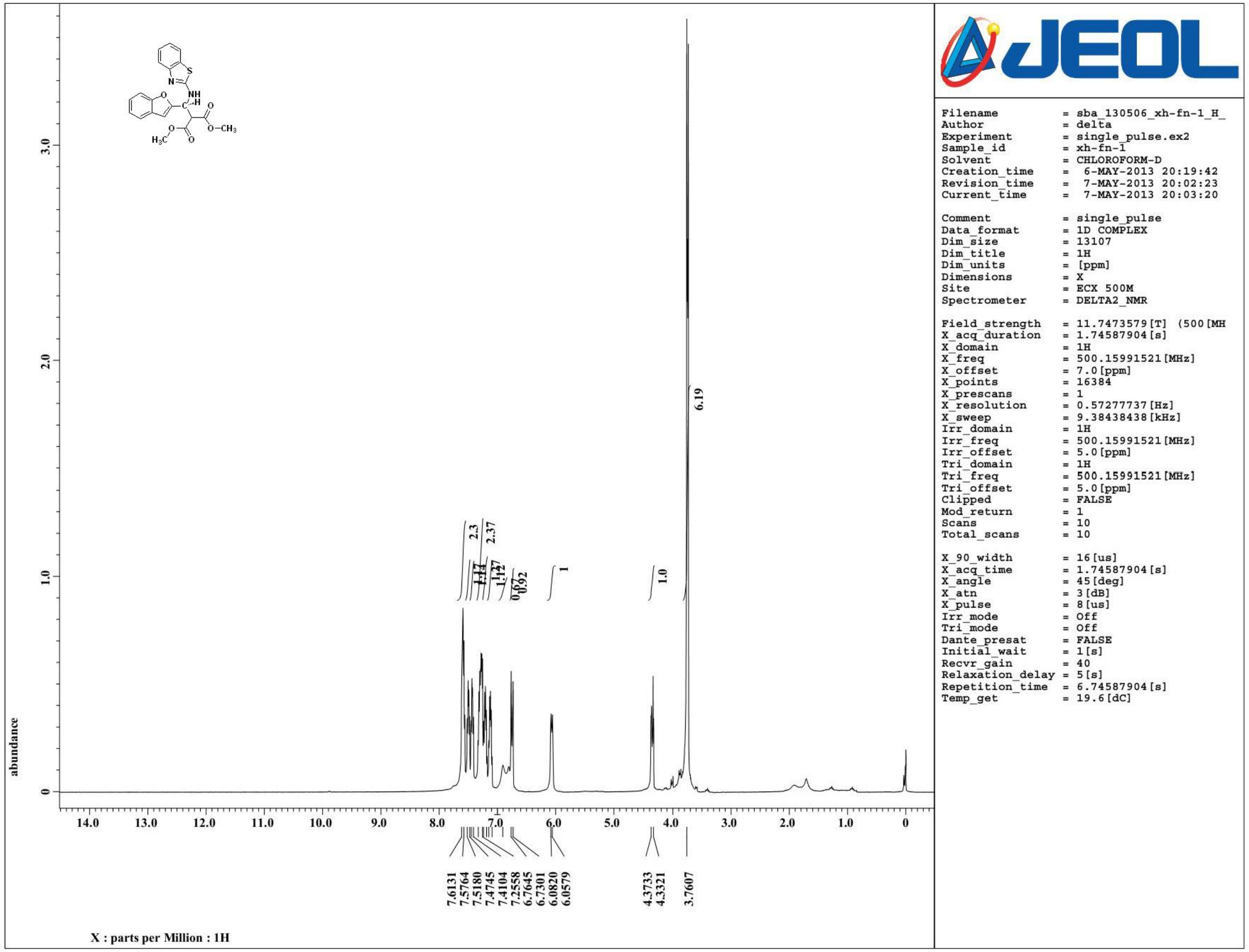

Figure S3. The ^{13}C-NMR Spectra of **5a**.

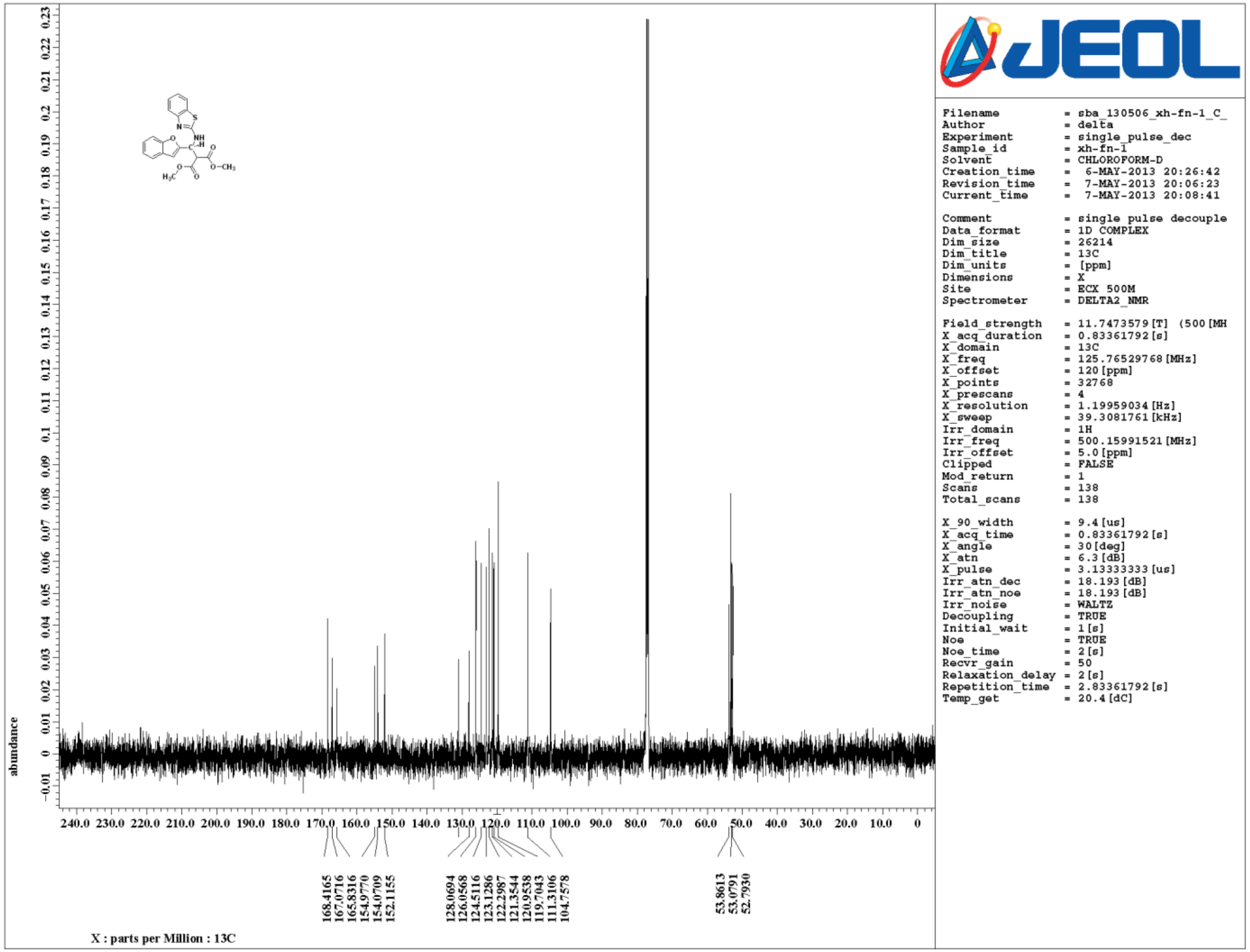

Figure S4. The IR spectra of **5a**.

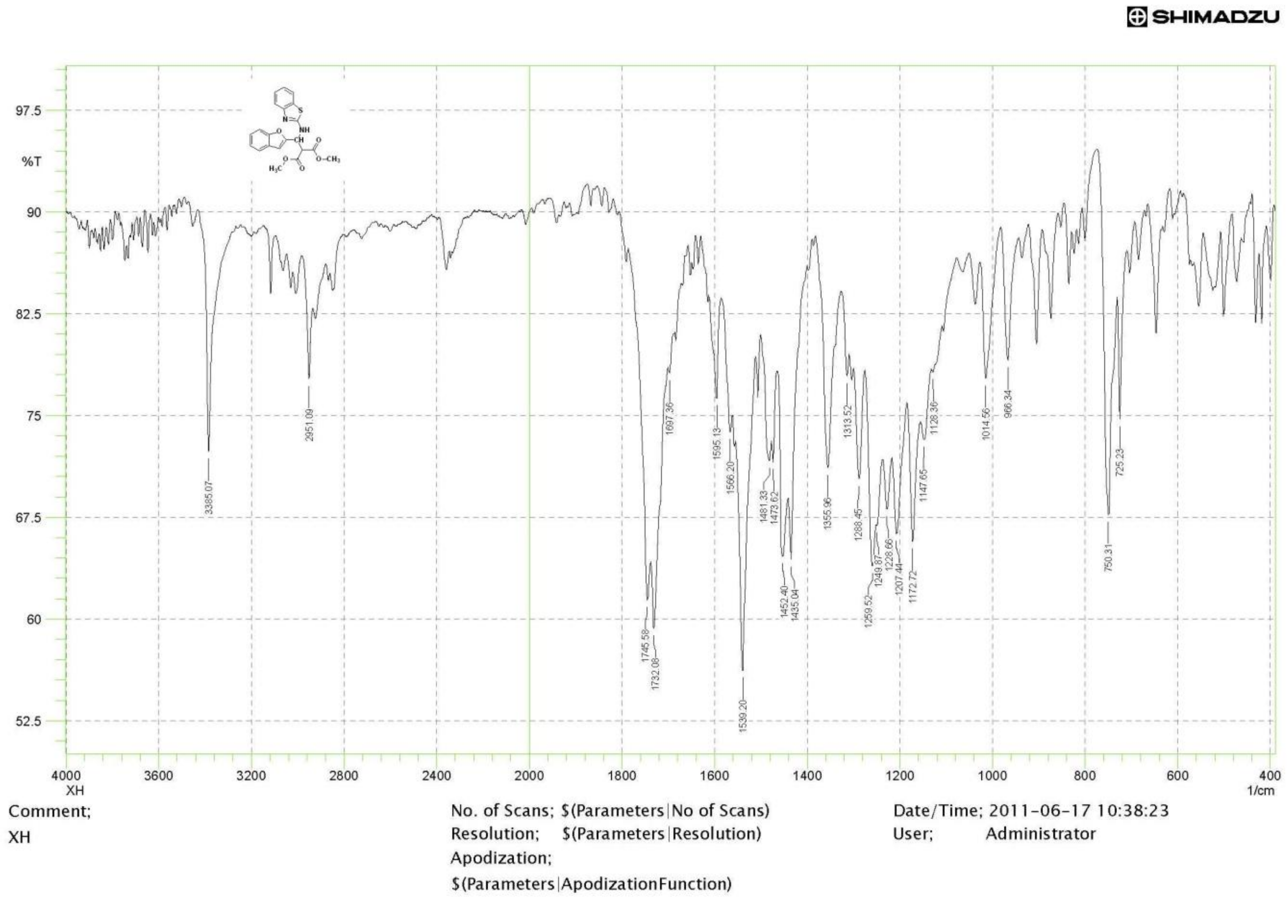

Diethyl 2-((benzo[d]thiazol-2-ylamino)(benzofuran-2-yl)methyl) malonate (**5b**)

Figure S5. The High Resolution Mass Spectra of **5b**.

Monoisotopic Mass, Odd and Even Electron Ions
17 formula(e) evaluated with 1 results within limits (up to 51 closest results for each mass)
Elements Used:
C: 0-200 H: 0-400 N: 2-2 O: 4-6 S: 1-1

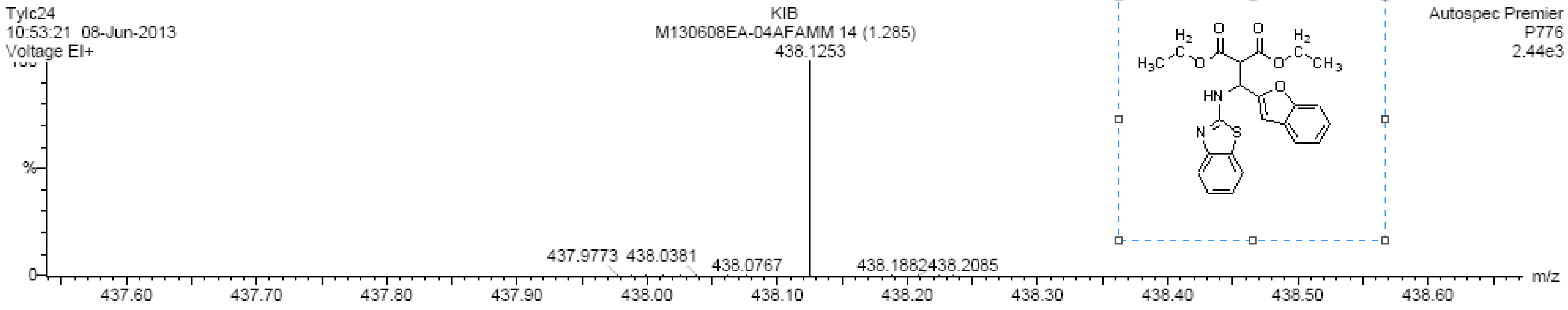

Minimum:				-10.0		
Maximum:		80.0	10.0	120.0		
Mass	Calc. Mass	mDa	PPM	DBE	i-FIT	Formula
438.1253	438.1249	0.4	0.9	14.0	5547234.5	C23 H22 N2 O5 S

Figure S6. The ^{1}H-NMR Spectra of **5b**.

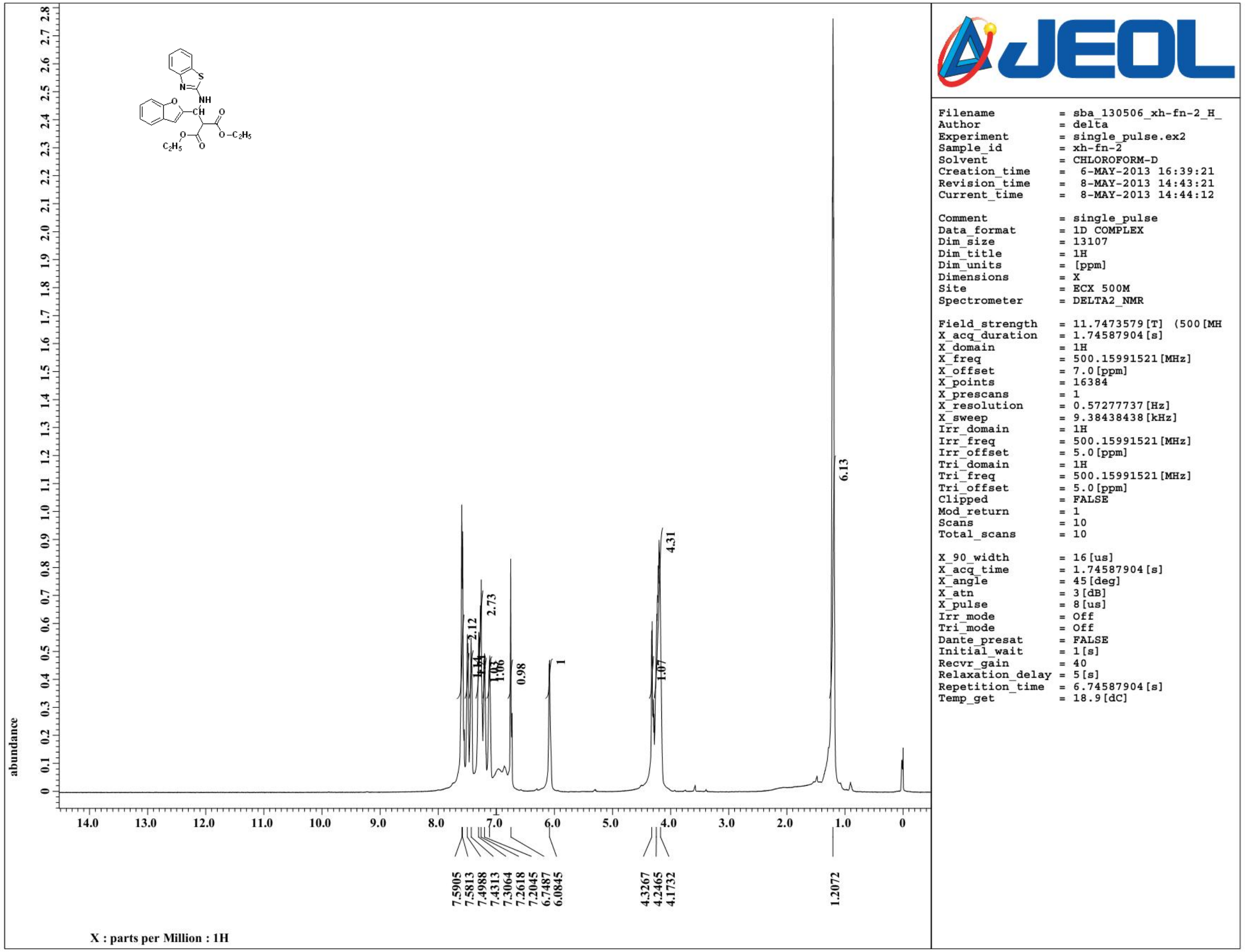

Figure S7. The ^{13}C-NMR Spectra of **5b**.

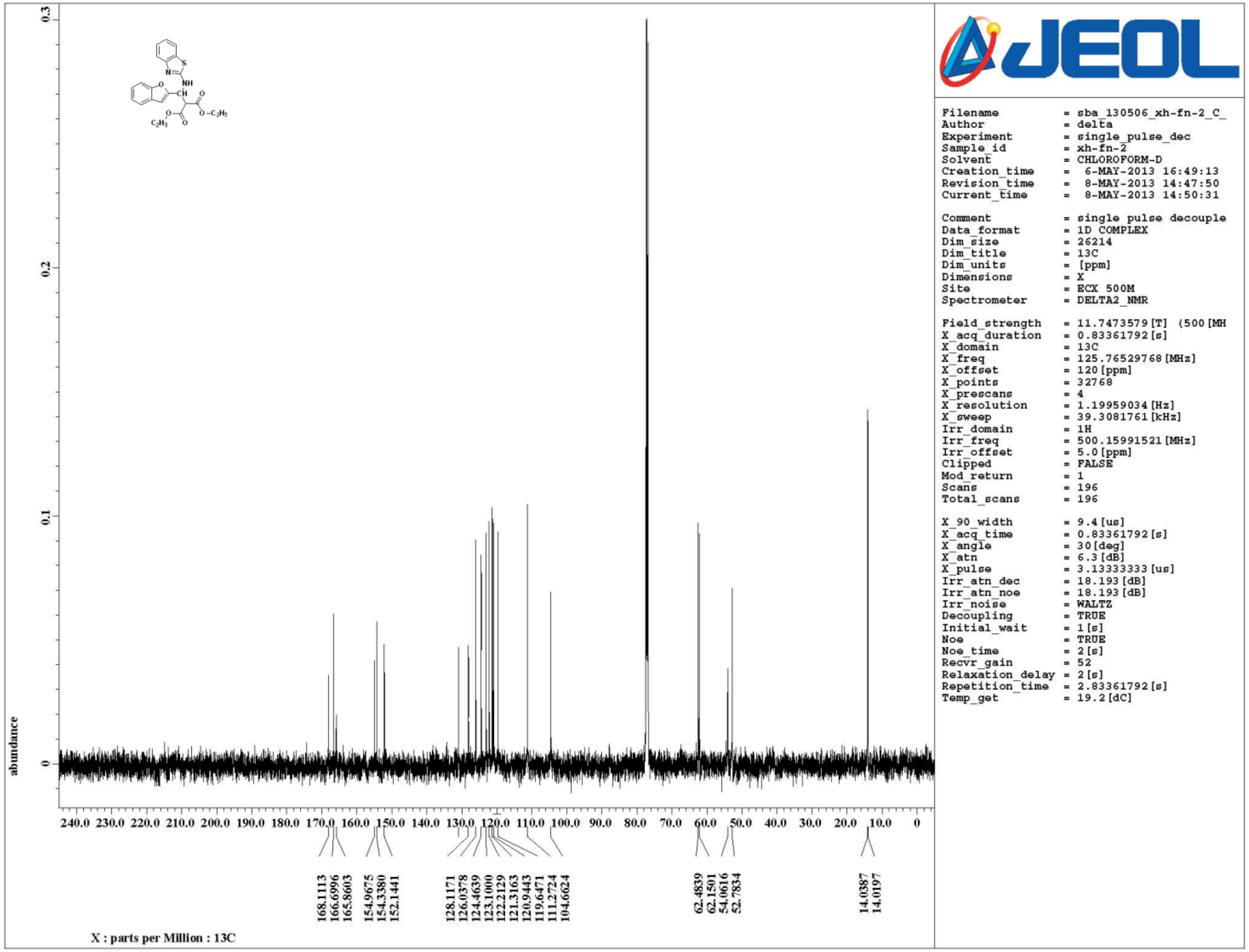

Figure S8. The IR spectra of **5b**.

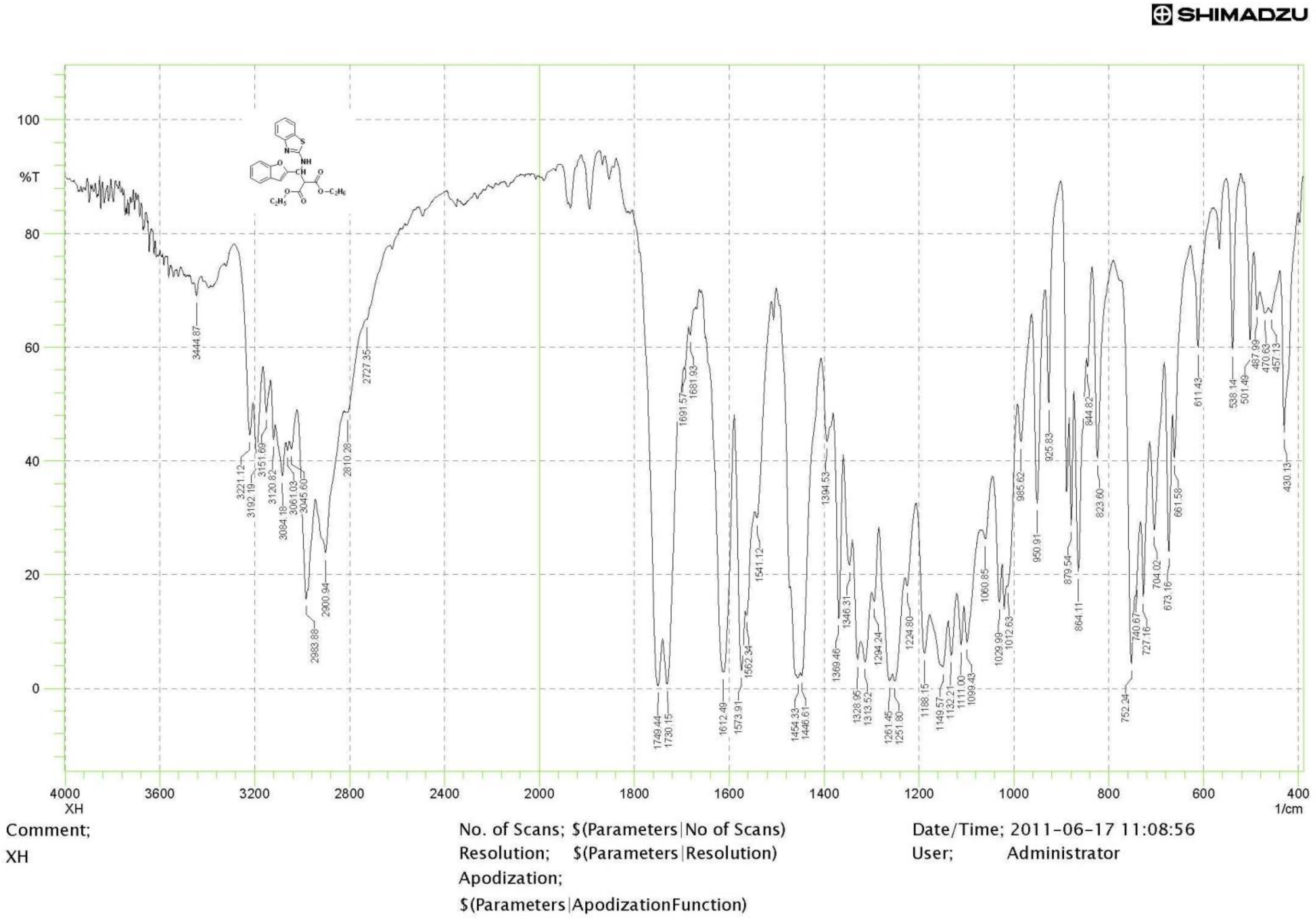

Dipropyl 2-((benzo[d]thiazol-2-ylamino)(benzofuran-2-yl)methyl) malonate (**5c**)

Figure S9. The High Resolution Mass Spectra of **5c**.

Monoisotopic Mass, Odd and Even Electron Ions
18 formula(e) evaluated with 1 results within limits (up to 51 closest results for each mass)
Elements Used:
C: 0-200 H: 0-400 N: 2-2 O: 4-6 S: 1-1

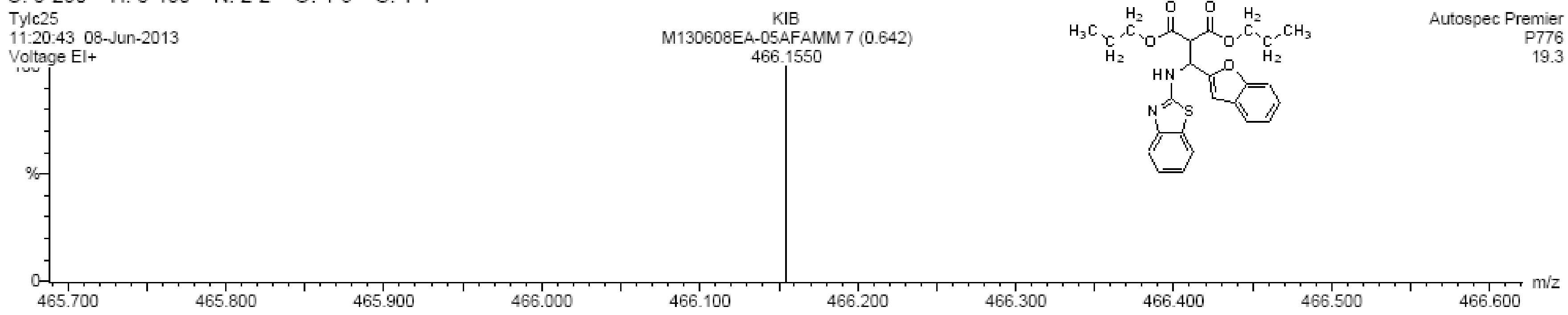

Minimum:				-10.0		
Maximum:		80.0	10.0	120.0		

Mass	Calc. Mass	mDa	PPM	DBE	i-FIT	Formula
466.1550	466.1562	-1.2	-2.6	14.0	5546029.0	C25 H26 N2 O5 S

Figure S10. The ^{1}H-NMR Spectra of **5c**.

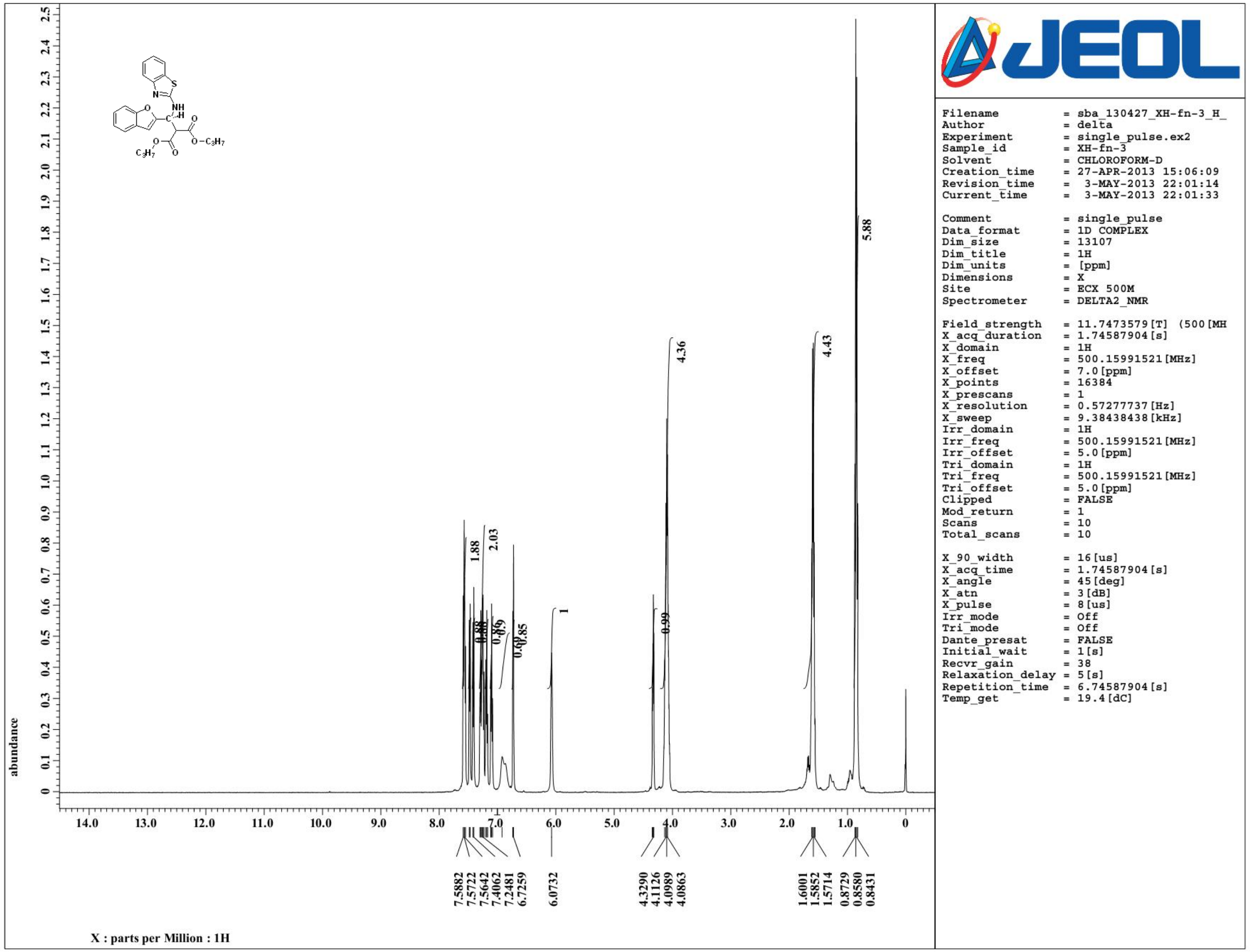

Figure S11. The ^{13}C-NMR Spectra of **5c**.

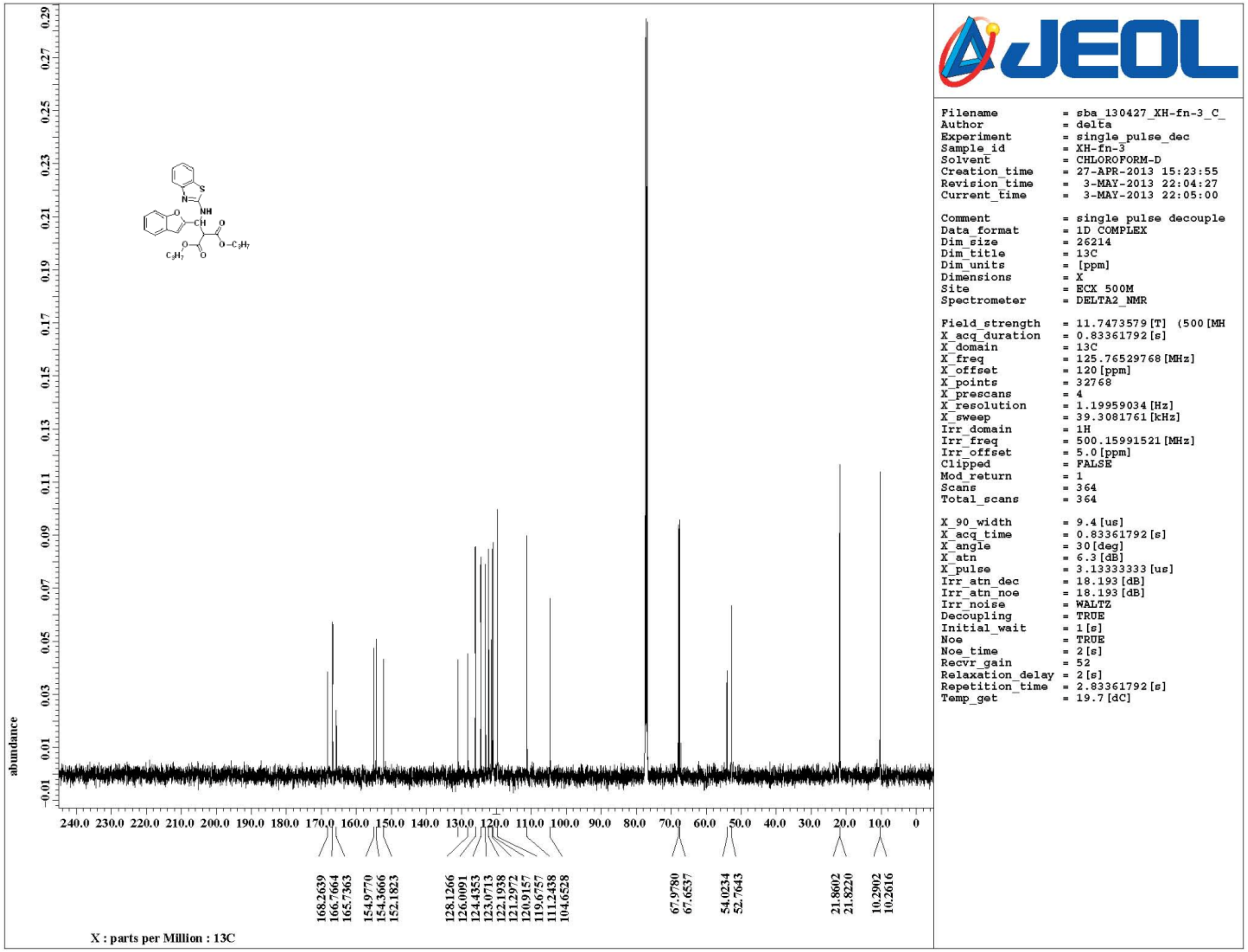

Figure S12. The IR spectra of **5c**.

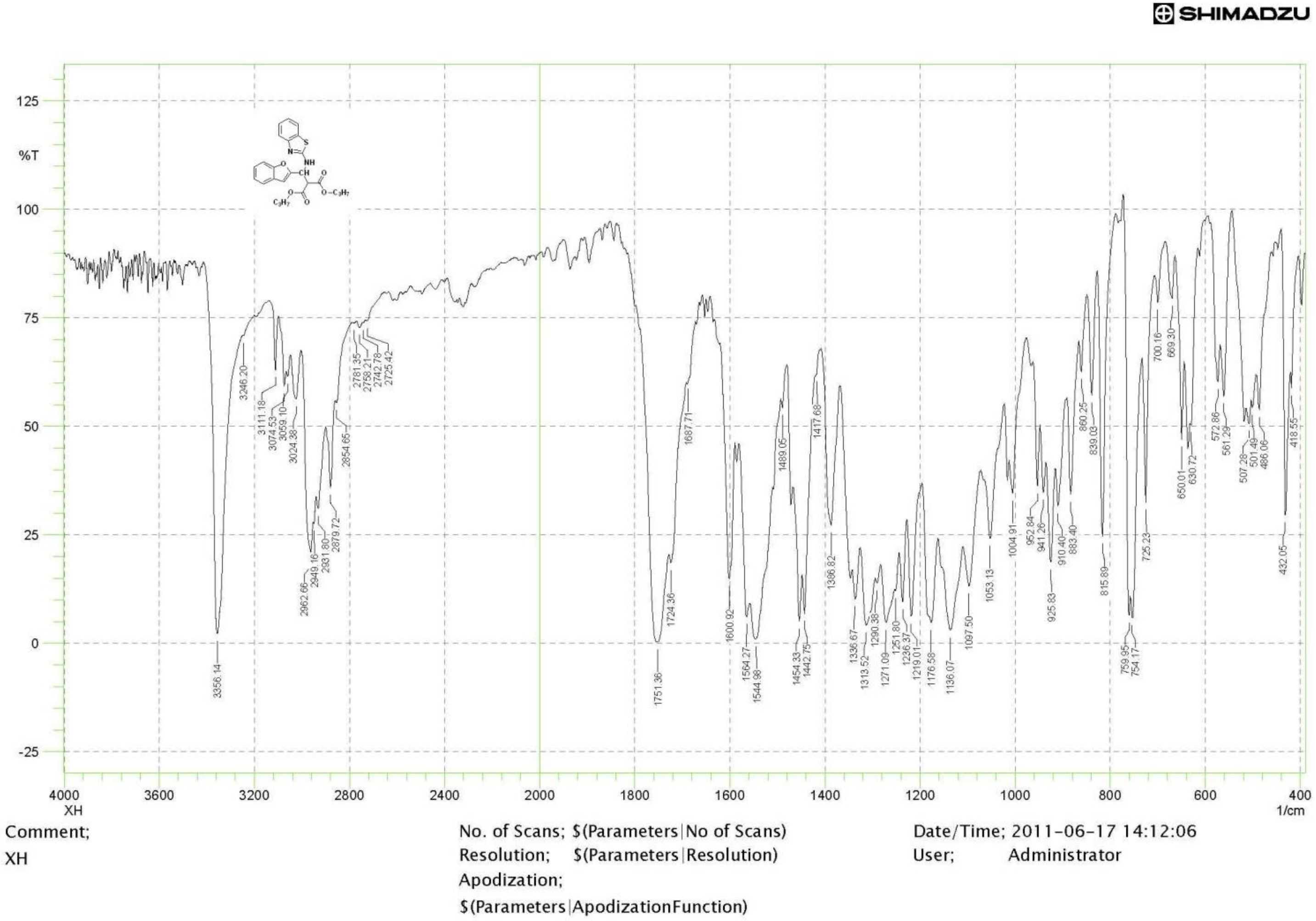

Dibenzyl 2-((benzo[d]thiazol-2-ylamino)(benzofuran-2-yl)methyl) malonate (**5d**)

Figure S13. The High Resolution Mass Spectra of **5d**.

Monoisotopic Mass, Odd and Even Electron Ions
22 formula(e) evaluated with 1 results within limits (up to 51 closest results for each mass)
Elements Used:
C: 0-200 H: 0-400 N: 2-2 O: 4-6 S: 1-1

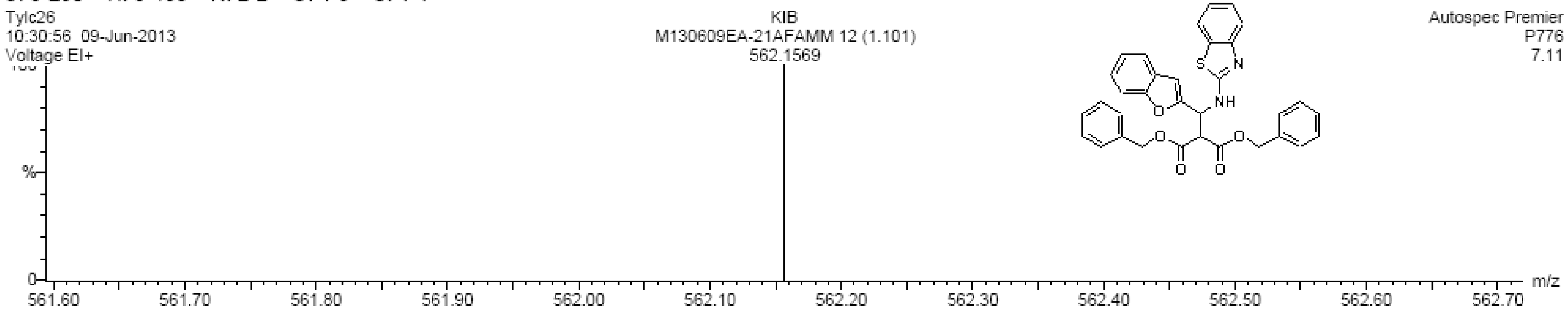

Minimum:				-10.0		
Maximum:		10.0	10.0	120.0		
Mass	Calc. Mass	mDa	PPM	DBE	i-FIT	Formula
562.1569	562.1562	0.7	1.2	22.0	5546026.0	C33 H26 N2 O5 S

Figure S14. The ^{1}H-NMR Spectra of **5d**.

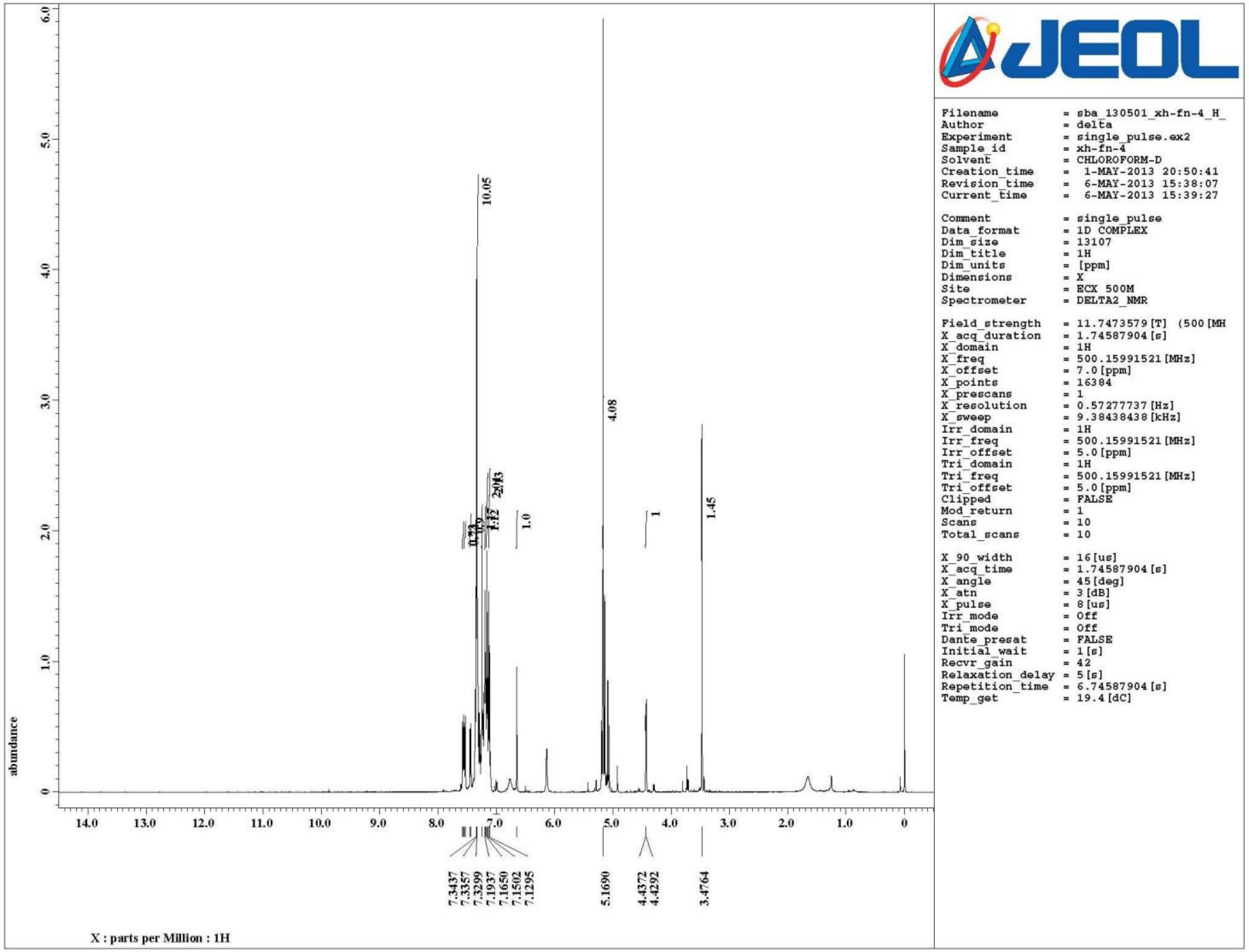

Figure S15. The ^{13}C-NMR Spectra of **5d**.

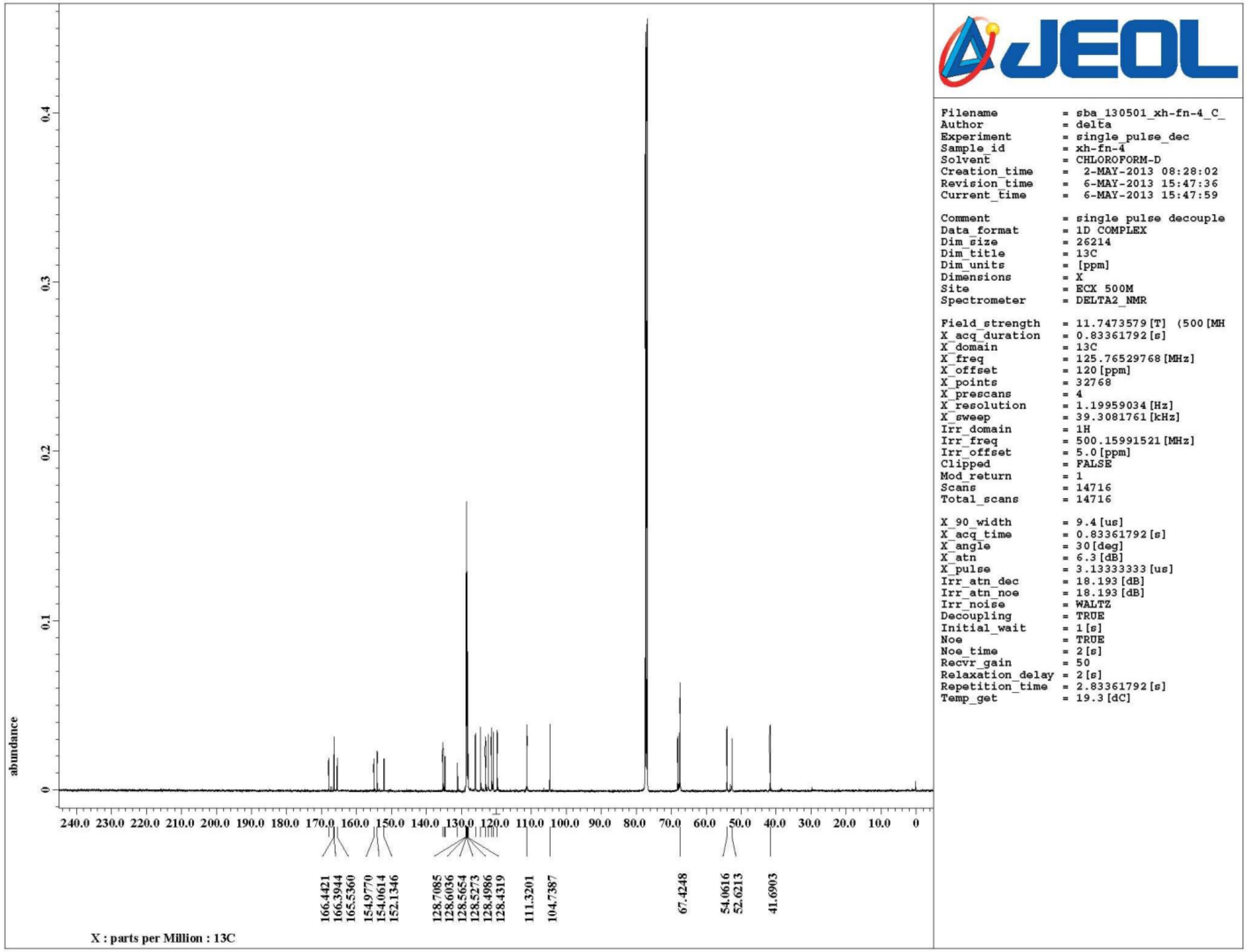

Figure S16. The IR spectra of **5d**.

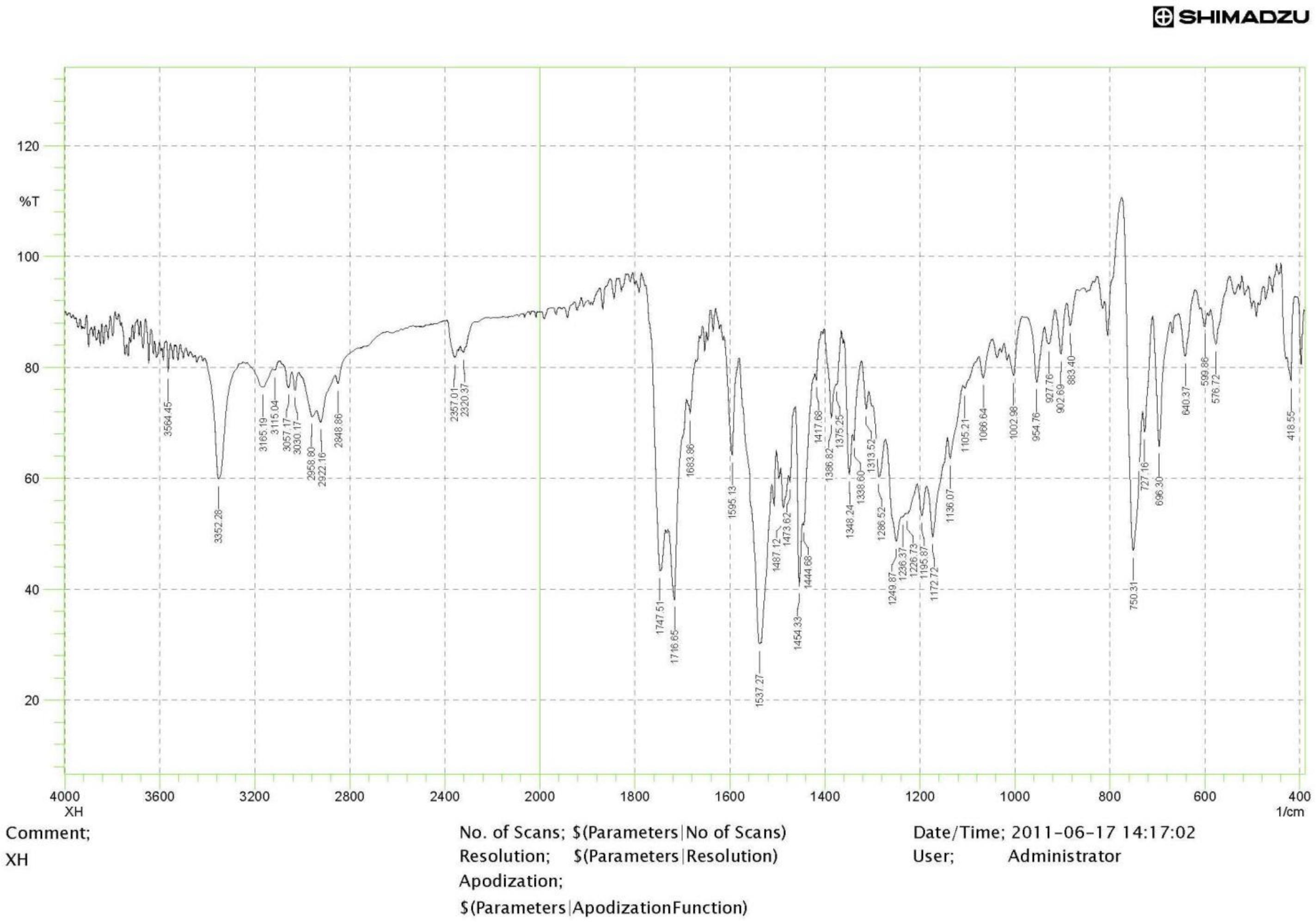

Dimethyl2-(benzofuran-2-yl((6-chlorobenzo[d]thiazol-2-yl)amino) methyl)malonate (**5e**)

Figure S17. The High Resolution Mass Spectra of **5e**.

Monoisotopic Mass, Odd and Even Electron Ions
16 formula(e) evaluated with 1 results within limits (up to 51 closest results for each mass)
Elements Used:
C: 0-200 H: 0-400 N: 2-2 O: 4-6 S: 1-1 Cl: 1-1

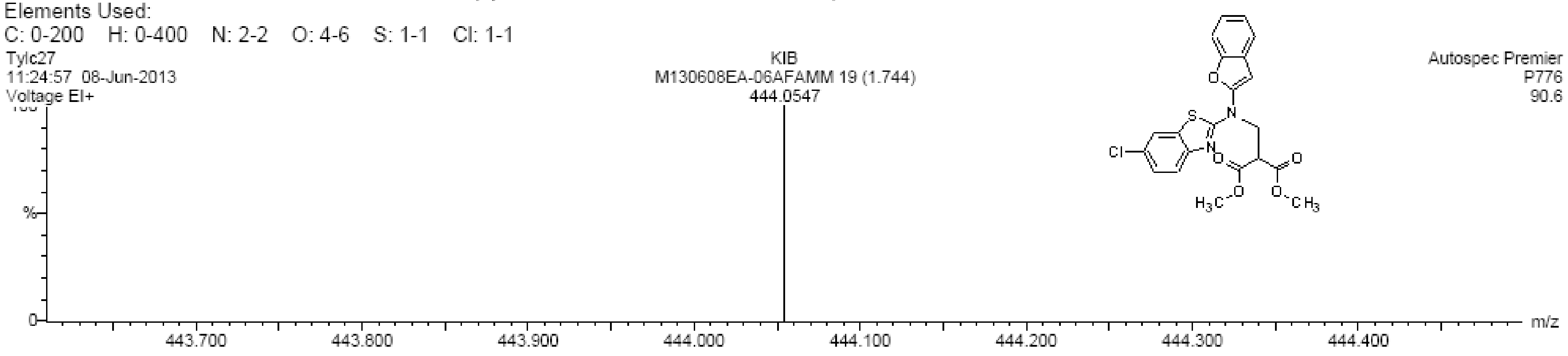

Minimum:				-10.0		
Maximum:		80.0	10.0	120.0		
Mass	Calc. Mass	mDa	PPM	DBE	i-FIT	Formula
444.0547	444.0547	0.0	0.0	14.0	5546065.5	C21 H17 N2 O5 S Cl

Figure S18. The ^{1}H-NMR Spectra of **5e**.

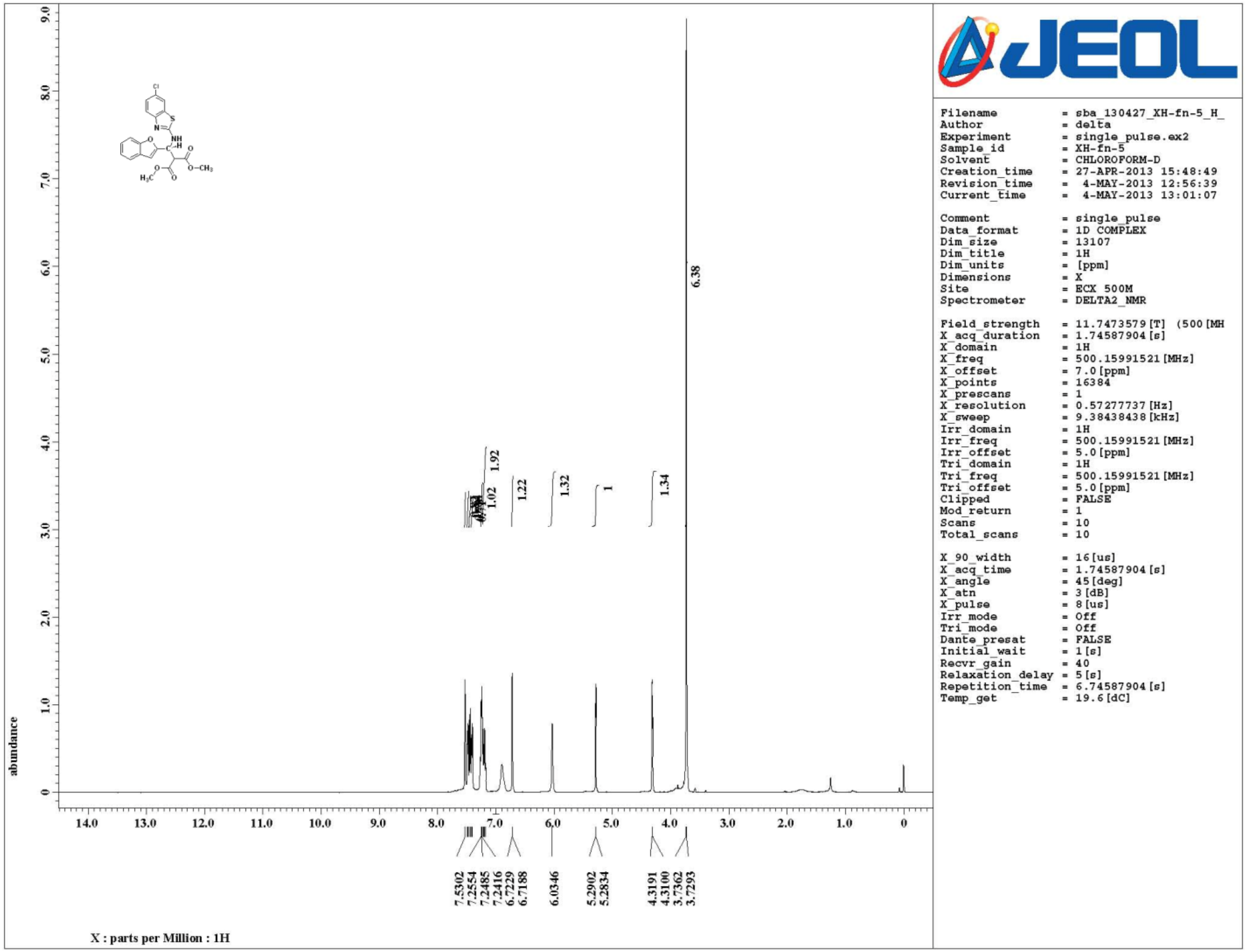

Figure S19. The ^{13}C-NMR Spectra of **5e**.

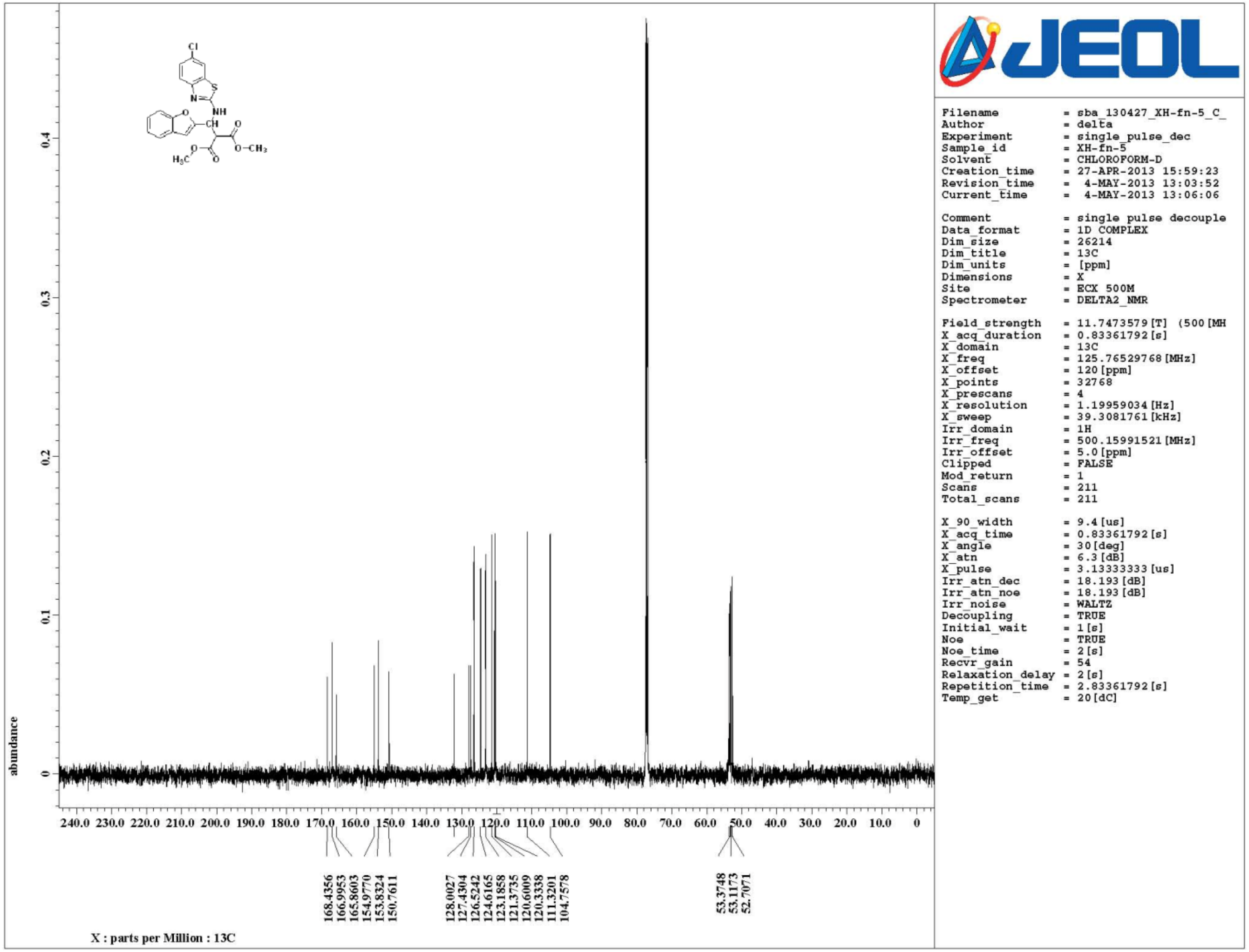

Figure S20. The IR spectra of **5e**.

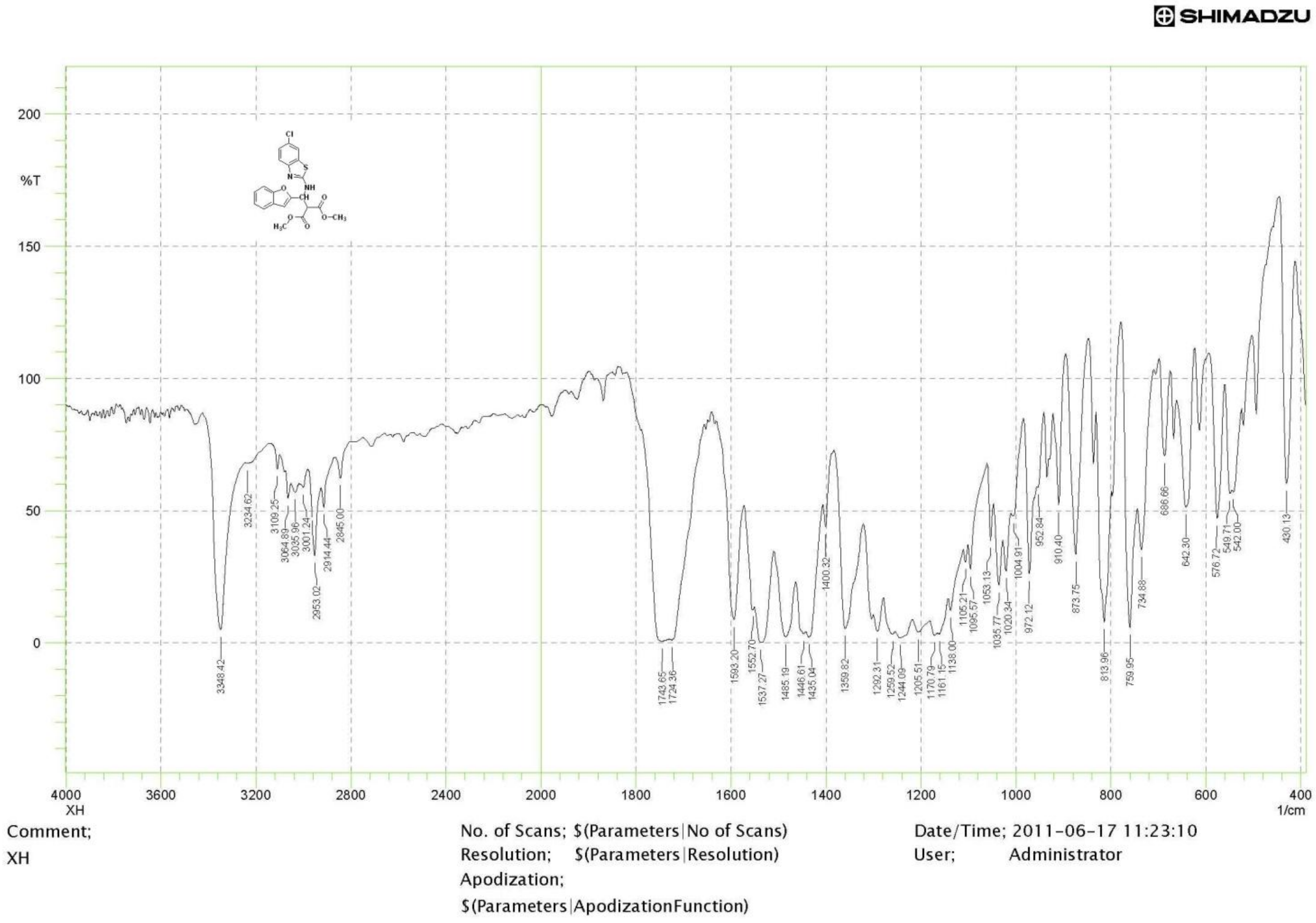

Diethyl 2-(benzofuran-2-yl((6-chlorobenzo[d]thiazol-2-yl)amino)methyl)malonate (**5f**)

Figure S21. The High Resolution Mass Spectra of **5f**.

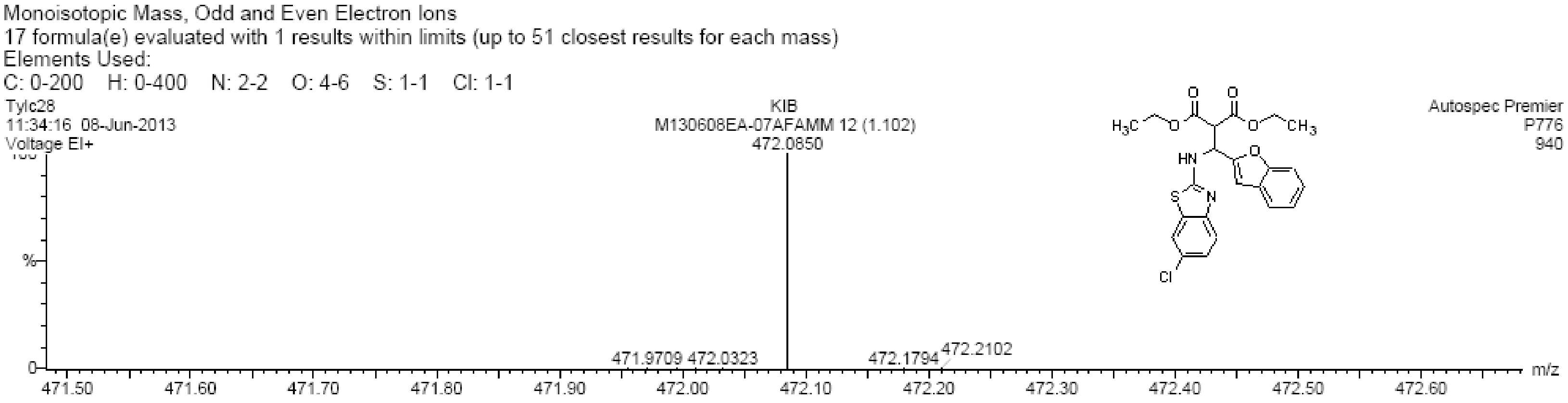

Minimum:				-10.0		
Maximum:		80.0	10.0	120.0		
Mass	Calc. Mass	mDa	PPM	DBE	i-FIT	Formula
472.0850	472.0860	-1.0	-2.1	14.0	5546490.5	C23 H21 N2 O5 S Cl

Figure S22. The ^{1}H-NMR Spectra of **5f**.

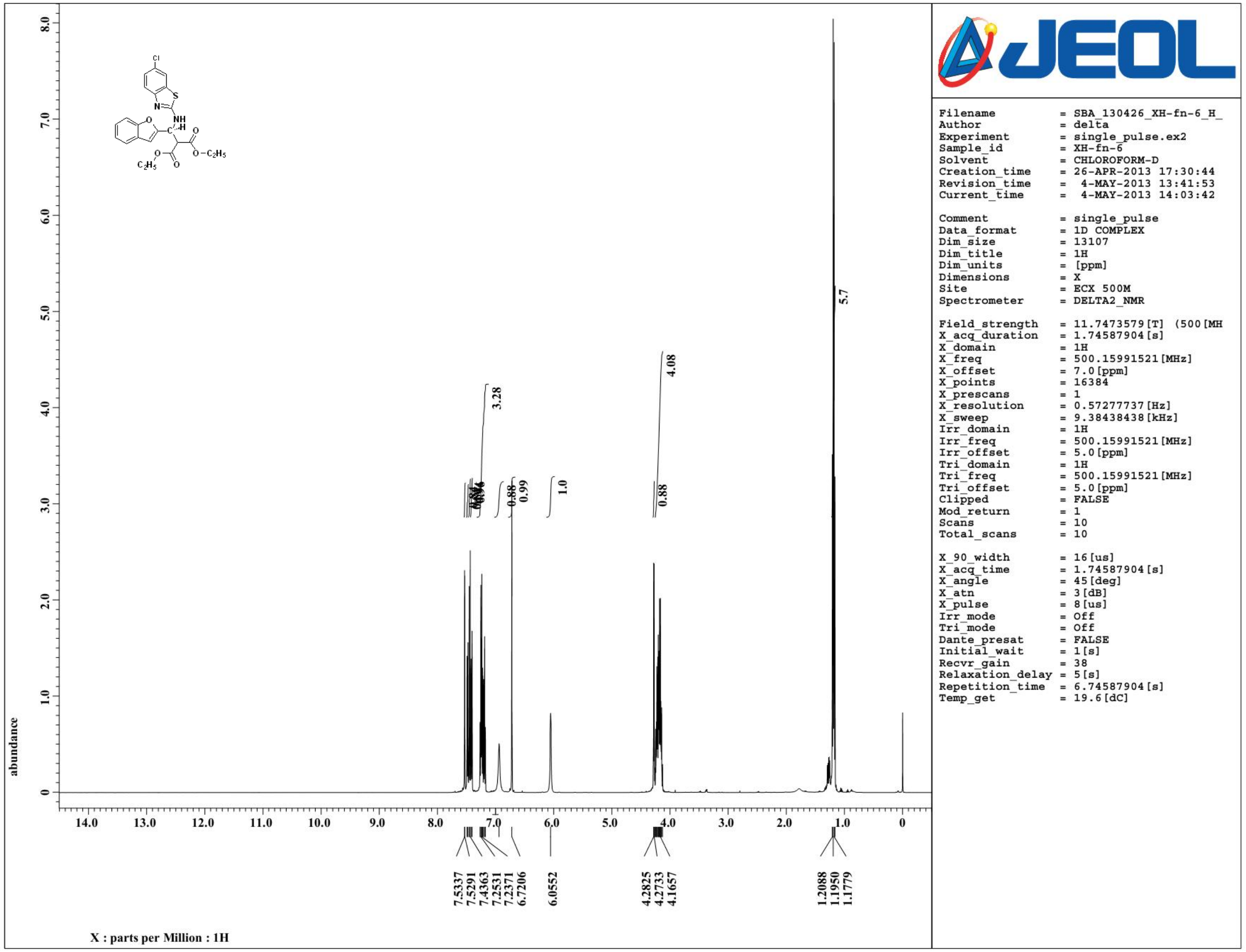

Figure S23. The ^{13}C-NMR Spectra of **5f**.

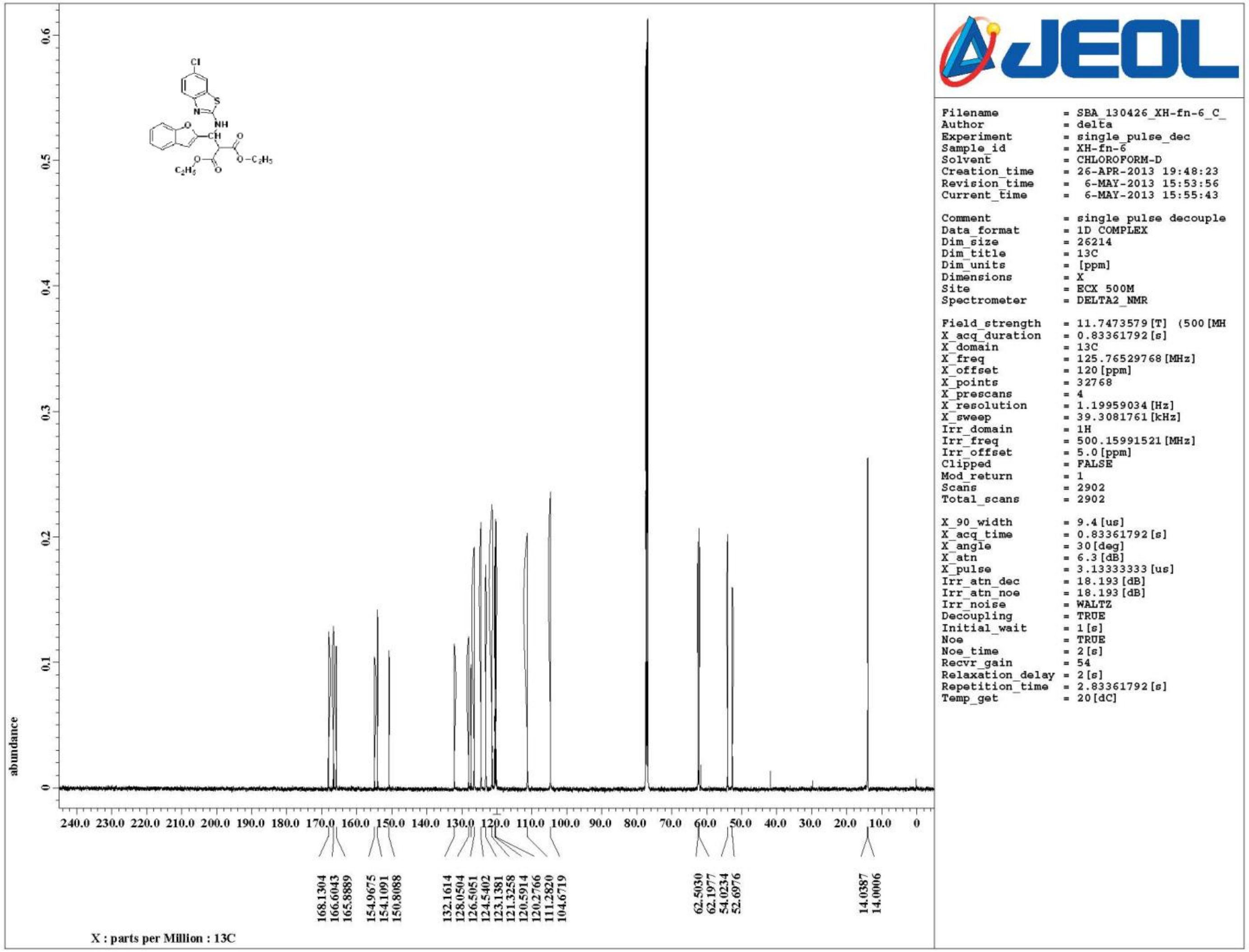

Figure S24. The IR spectra of **5f**.

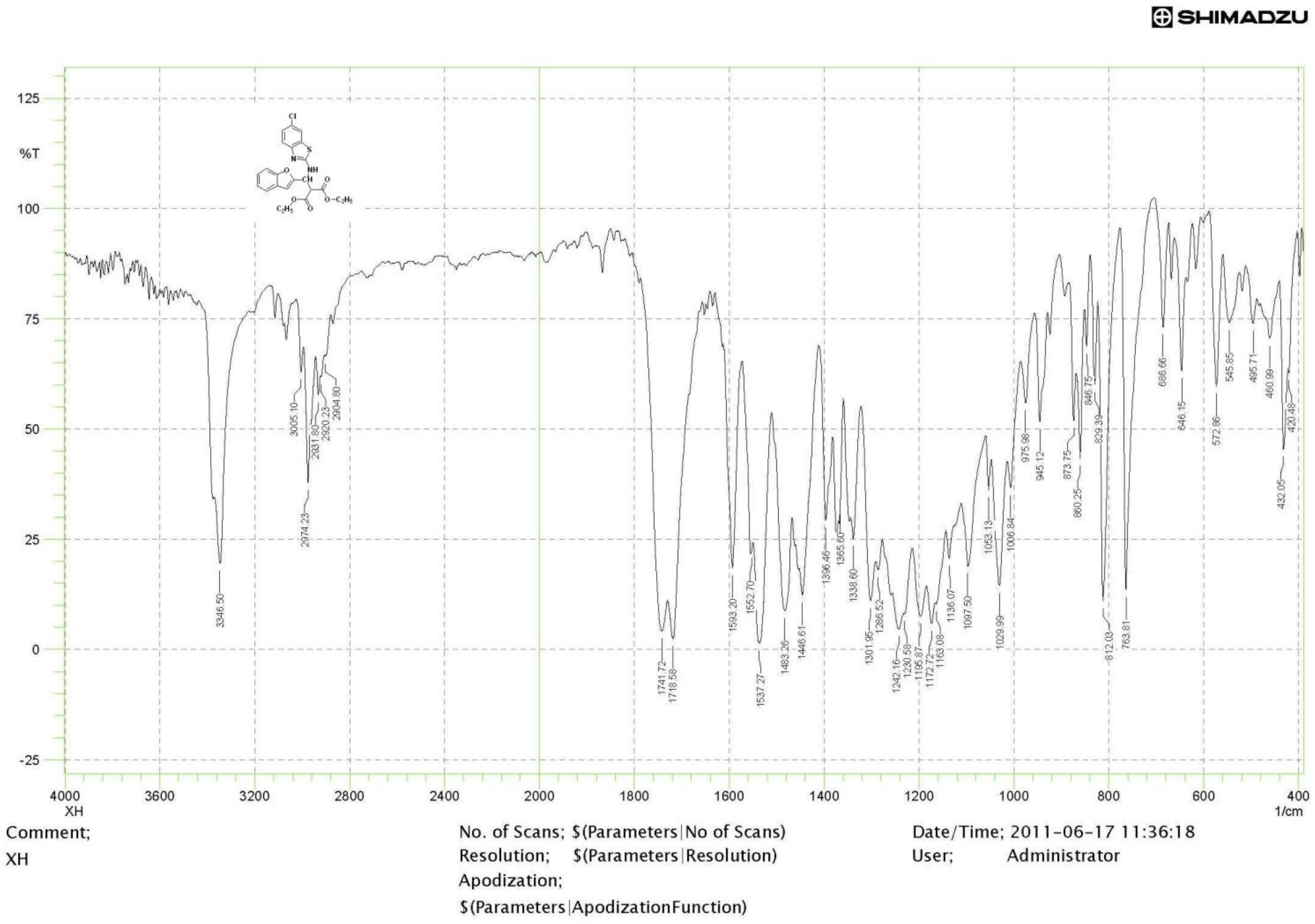

Dipropyl2-(benzofuran-2-yl((6-chlorobenzo[d]thiazol-2-yl)amino) methyl)malonate (**5g**)

Figure S25. The High Resolution Mass Spectra of **5g**.

Monoisotopic Mass, Odd and Even Electron Ions
18 formula(e) evaluated with 1 results within limits (up to 51 closest results for each mass)
Elements Used:
C: 0-200 H: 0-400 N: 2-2 O: 4-6 S: 1-1 Cl: 1-1

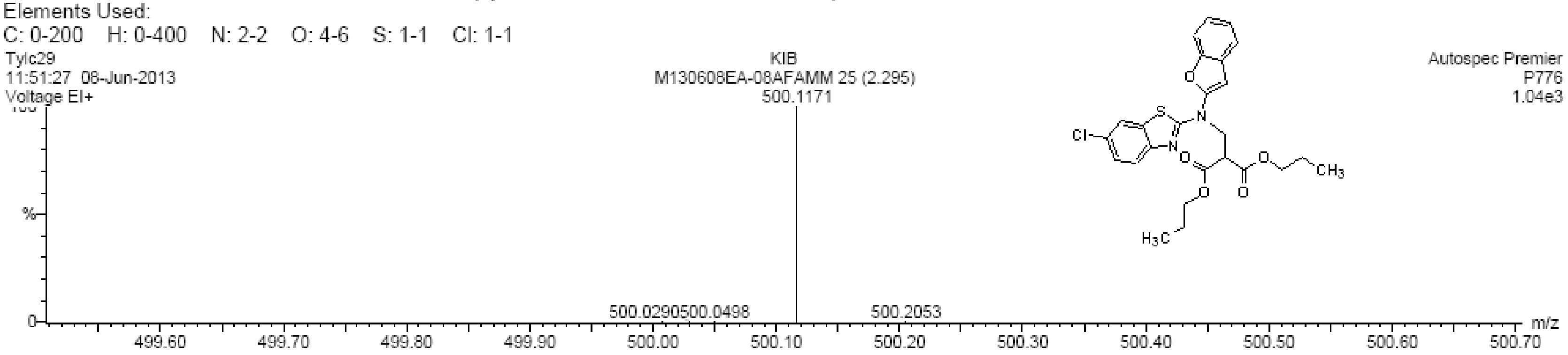

Minimum:				-10.0		
Maximum:		80.0	10.0	120.0		
Mass	Calc. Mass	mDa	PPM	DBE	i-FIT	Formula
500.1171	500.1173	-0.2	-0.4	14.0	5546539.0	C25 H25 N2 O5 S Cl

Figure S26. The ^{1}H-NMR Spectra of **5g**.

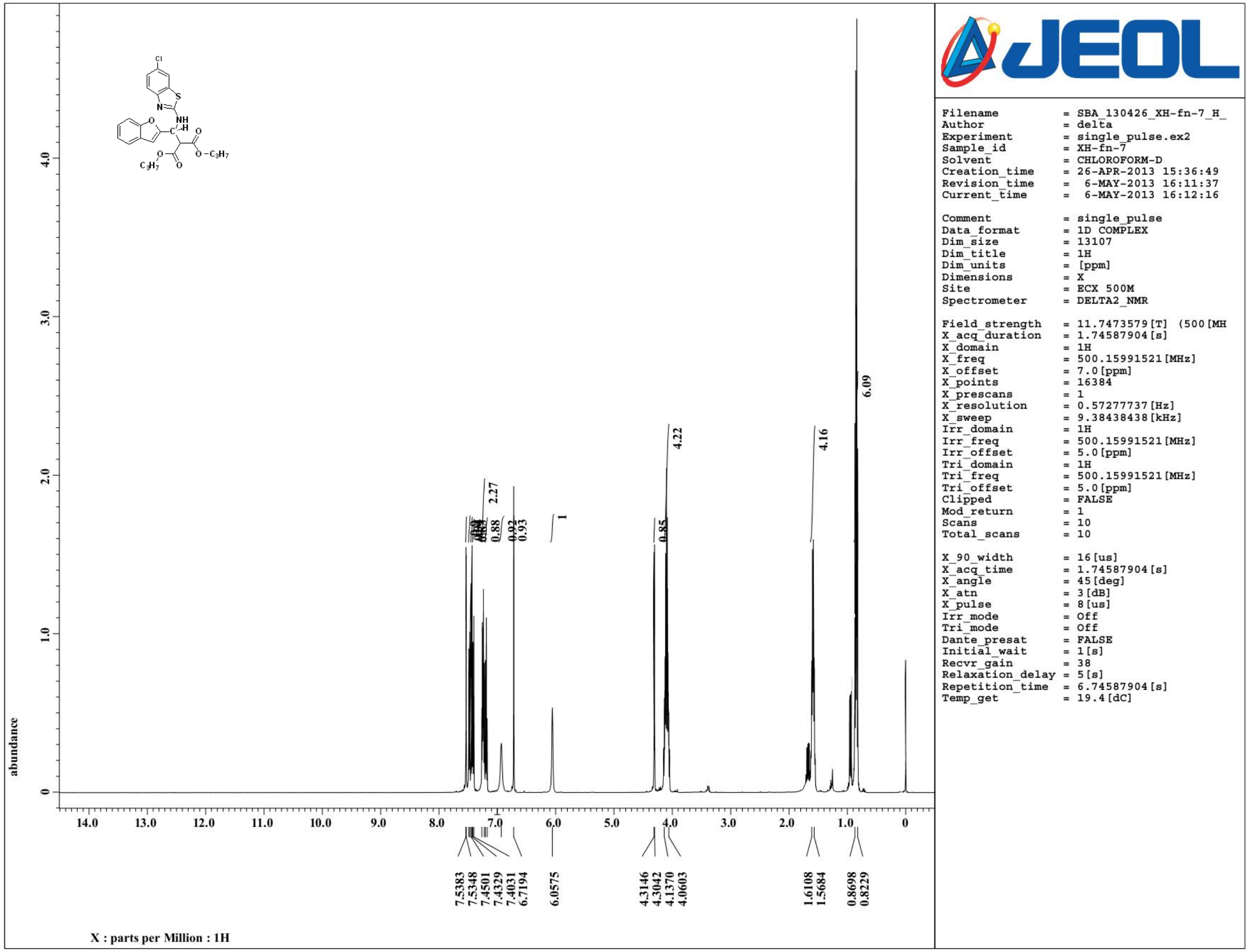

Figure S27. The ^{13}C-NMR Spectra of **5g**.

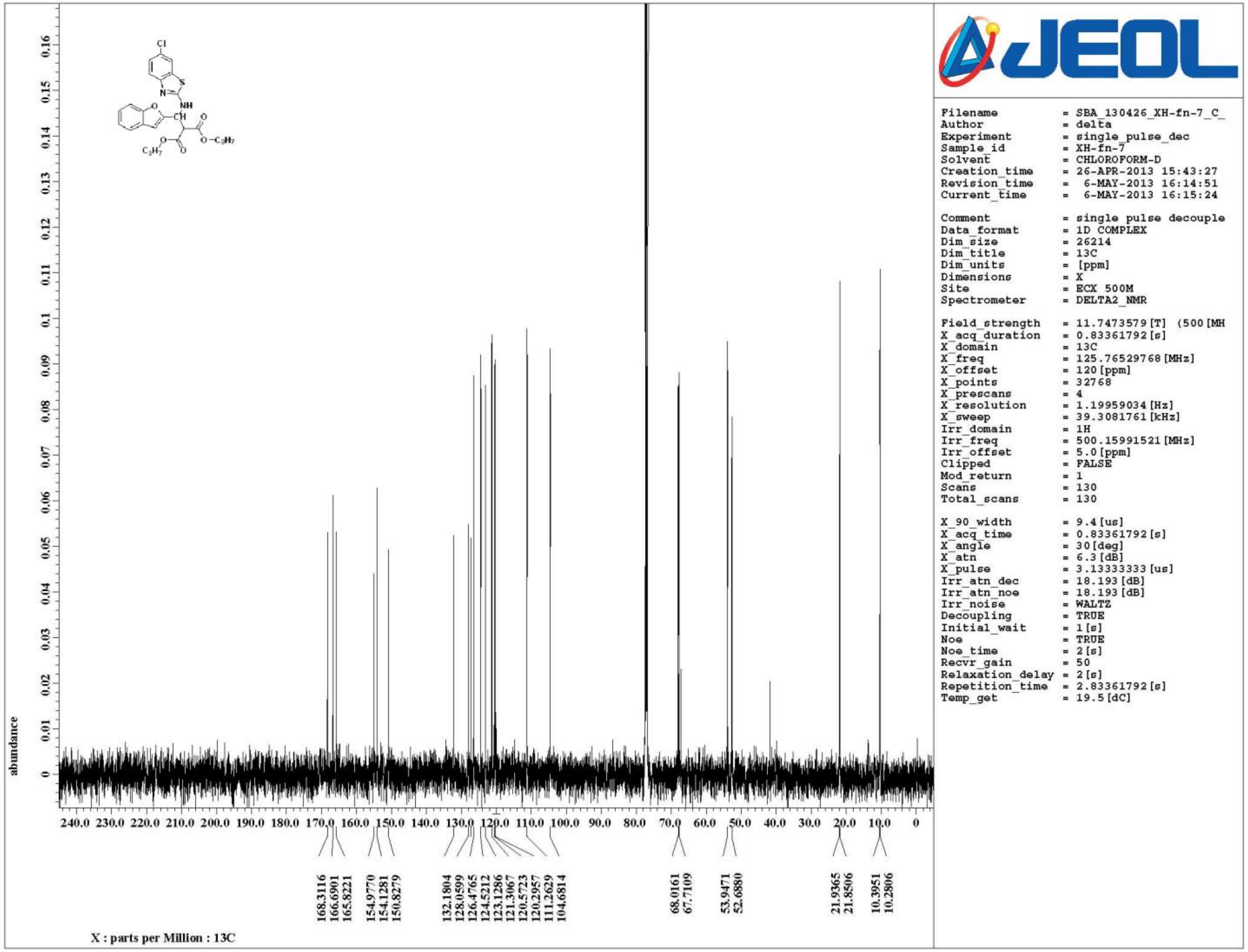

Figure S28. The IR spectra of **5g**.

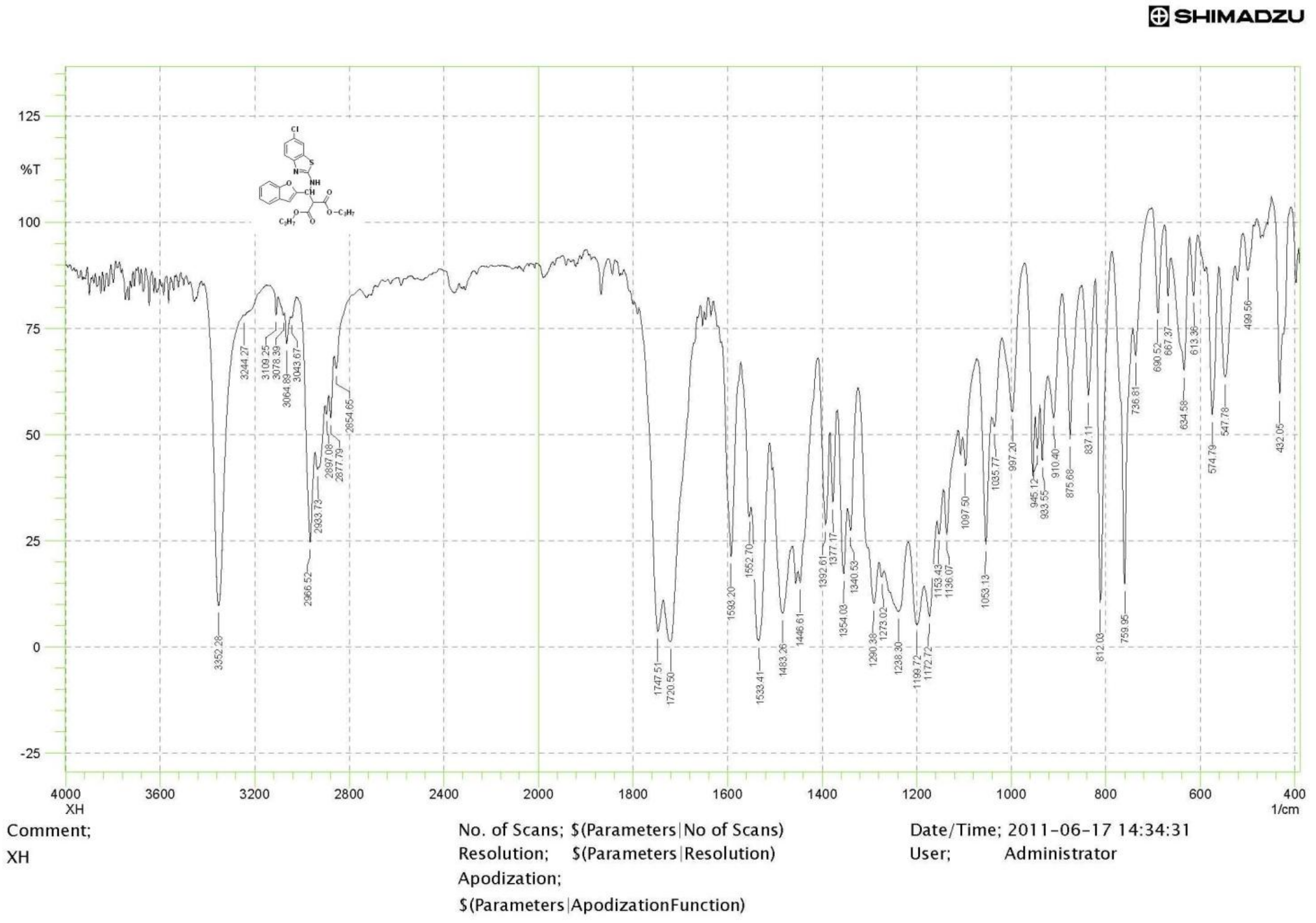

Dibenzyl2-(benzofuran-2-yl((6-chlorobenzo[d]thiazol-2-yl)amino)methyl)malonate (**5h**)

Figure S29. The High Resolution Mass Spectra of **5h**.

Monoisotopic Mass, Odd and Even Electron Ions
22 formula(e) evaluated with 1 results within limits (up to 51 closest results for each mass)
Elements Used:
C: 0-200 H: 0-400 N: 2-2 O: 4-6 S: 1-1 Cl: 1-1

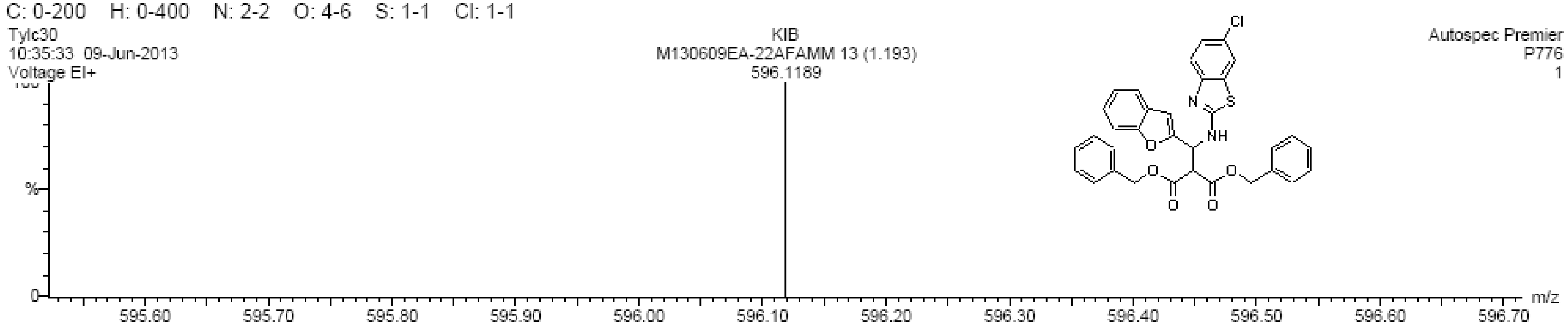

Minimum:				-10.0		
Maximum:		10.0	10.0	120.0		
Mass	Calc. Mass	mDa	PPM	DBE	i-FIT	Formula
596.1189	596.1173	1.6	2.7	22.0	5546025.5	C33 H25 N2 O5 S Cl

Figure S30. The ^{1}H-NMR Spectra of **5h**.

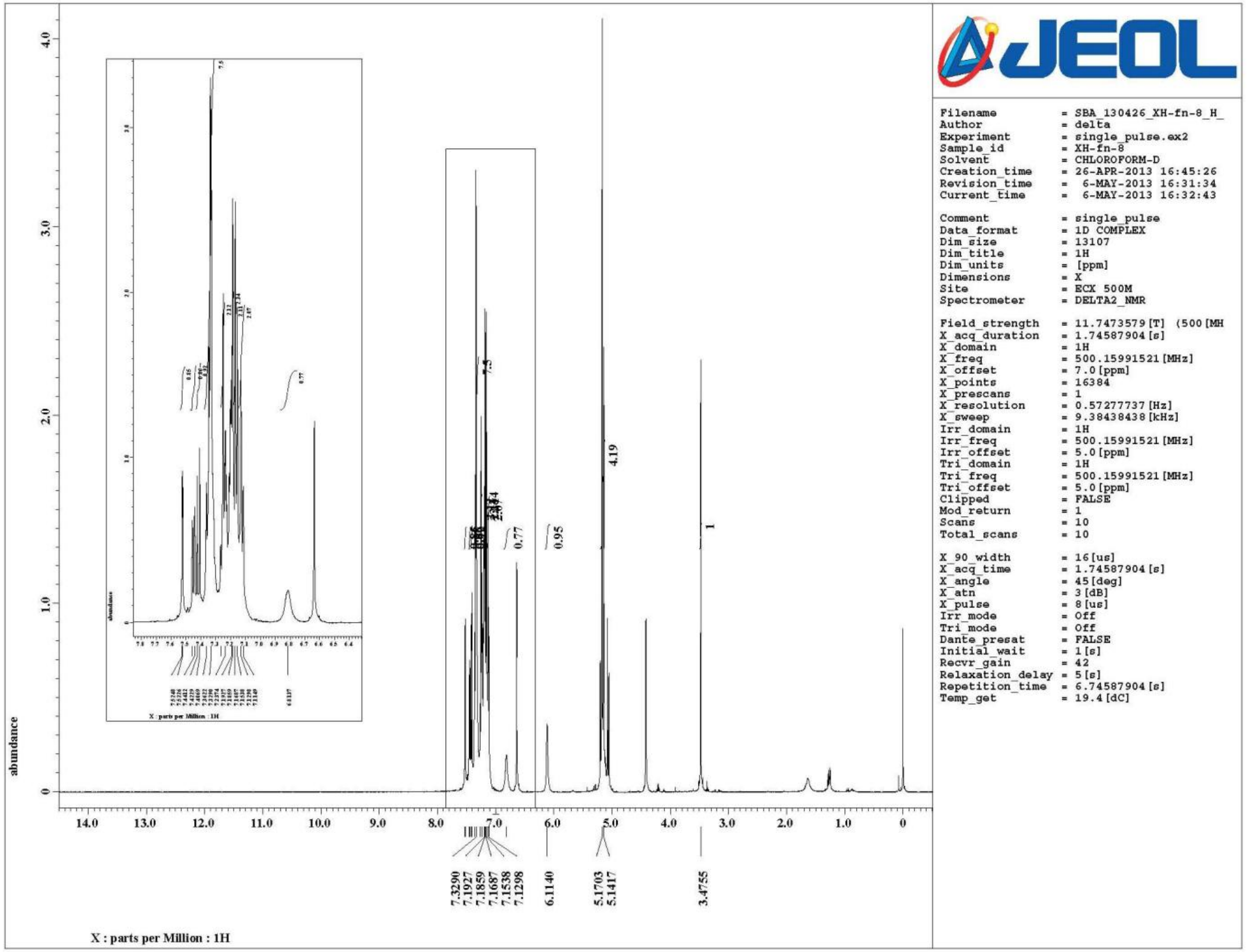

Figure S31. The ^{13}C-NMR Spectra of **5h**.

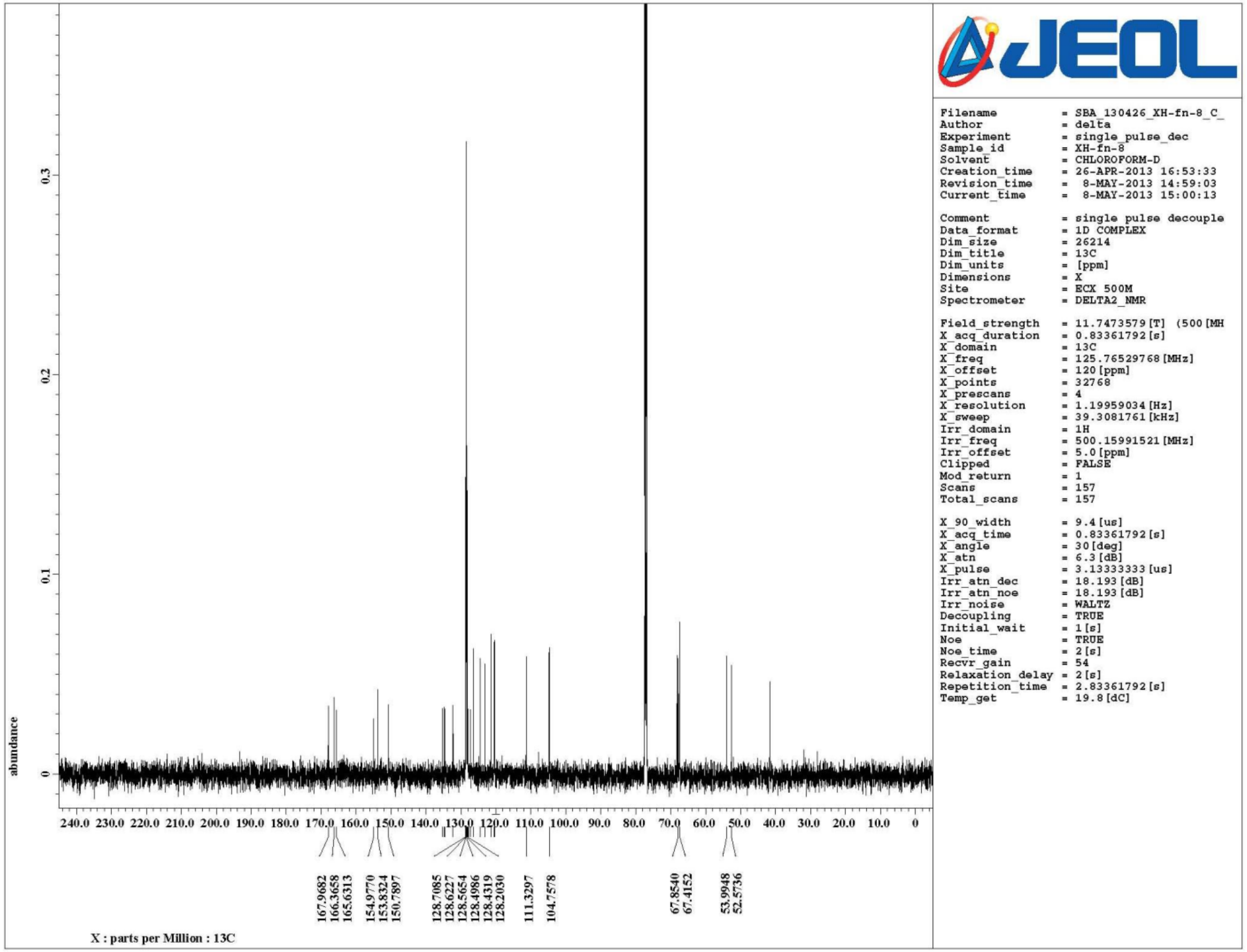

Figure S32. The IR spectra of **5h**.

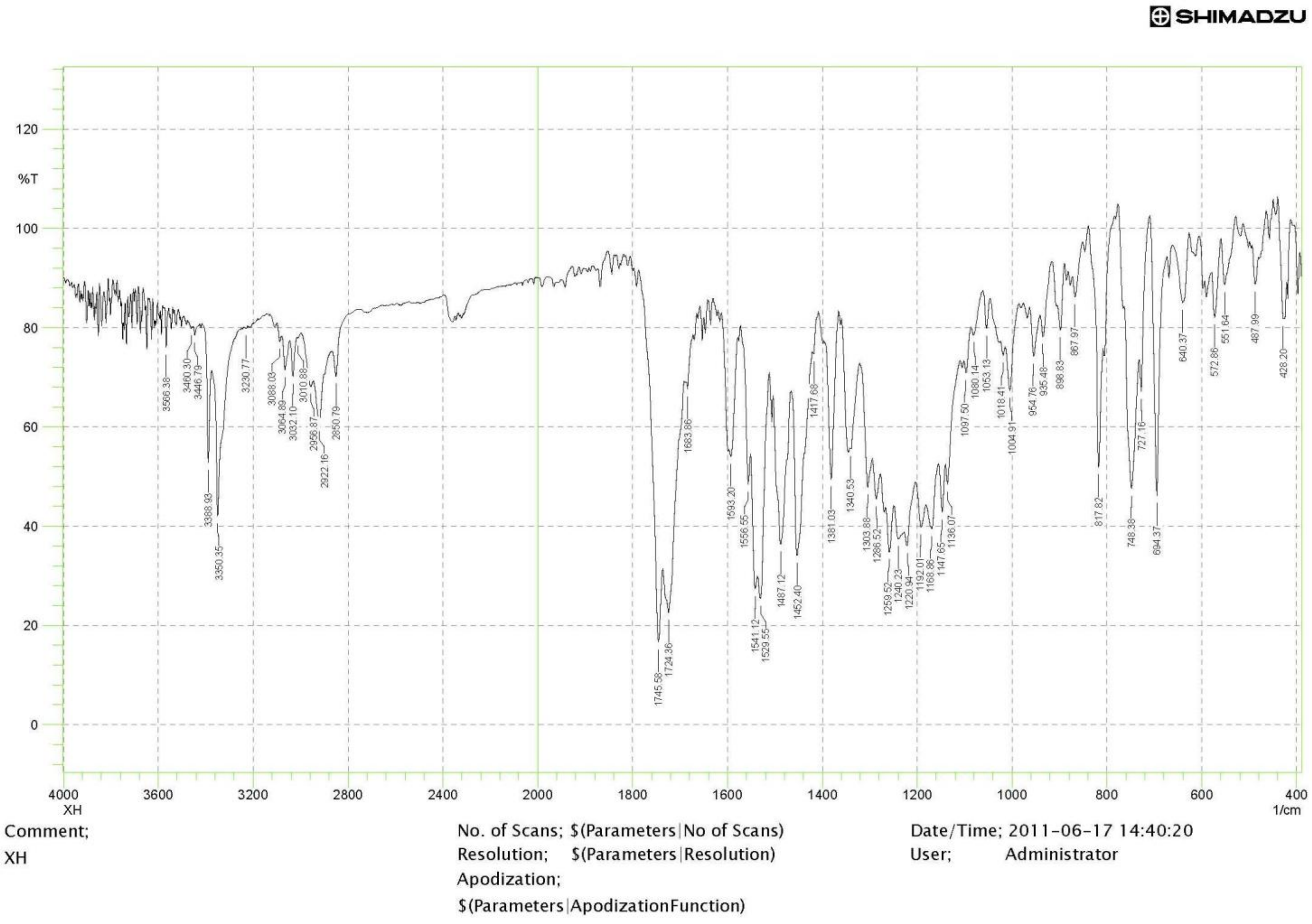

Dimethyl 2-(benzofuran-2-yl((6-methoxybenzo[d]thiazol-2-yl)amino)methyl)malonate (**5i**)

Figure S33. The High Resolution Mass Spectra of **5i**.

Monoisotopic Mass, Odd and Even Electron Ions
17 formula(e) evaluated with 1 results within limits (up to 51 closest results for each mass)
Elements Used:
C: 0-200 H: 0-400 N: 2-2 O: 5-7 S: 1-1

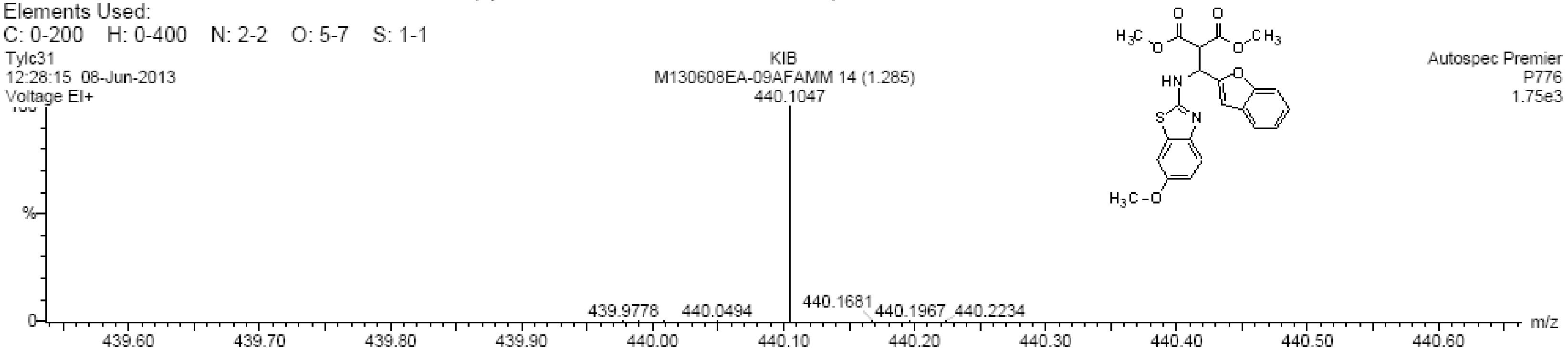

Minimum:				-10.0		
Maximum:		200.0	10.0	120.0		
Mass	Calc. Mass	mDa	PPM	DBE	i-FIT	Formula
440.1047	440.1042	0.5	1.1	14.0	5546887.5	C22 H20 N2 O6 S

Figure S34. The ^{1}H-NMR Spectra of **5i**.

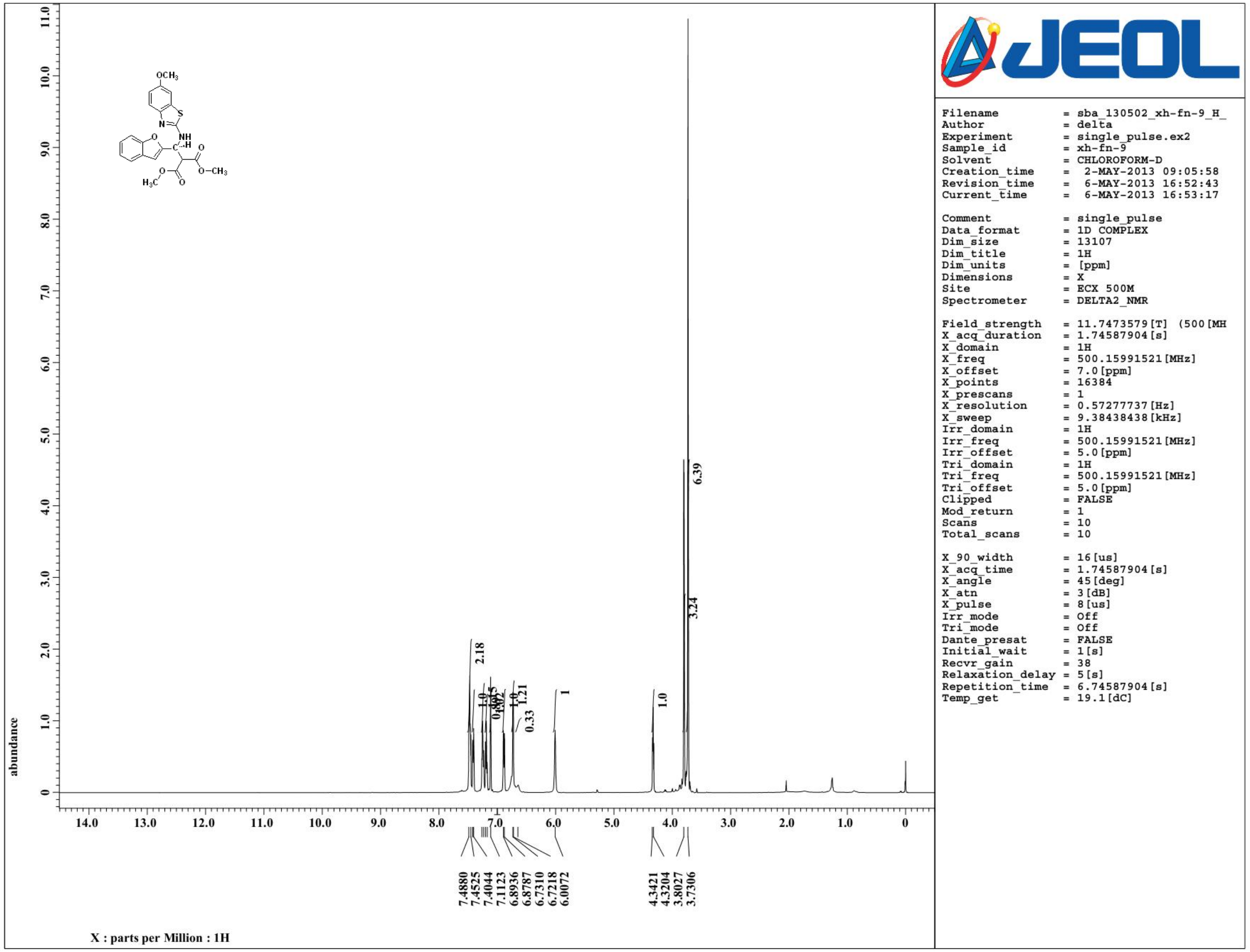

Figure S35. The ^{13}C-NMR Spectra of **5i**.

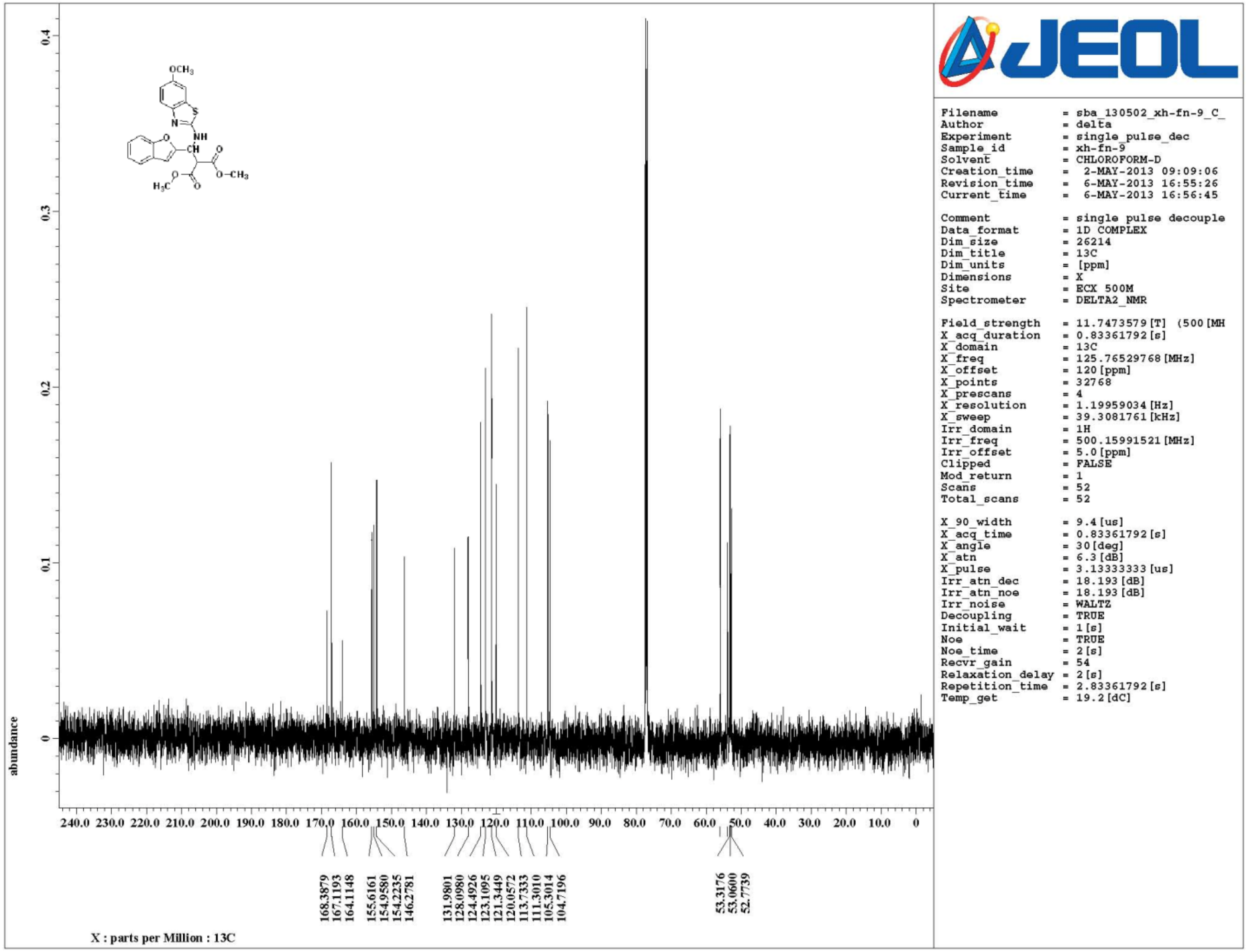

Figure S36. The IR spectra of **5i**.

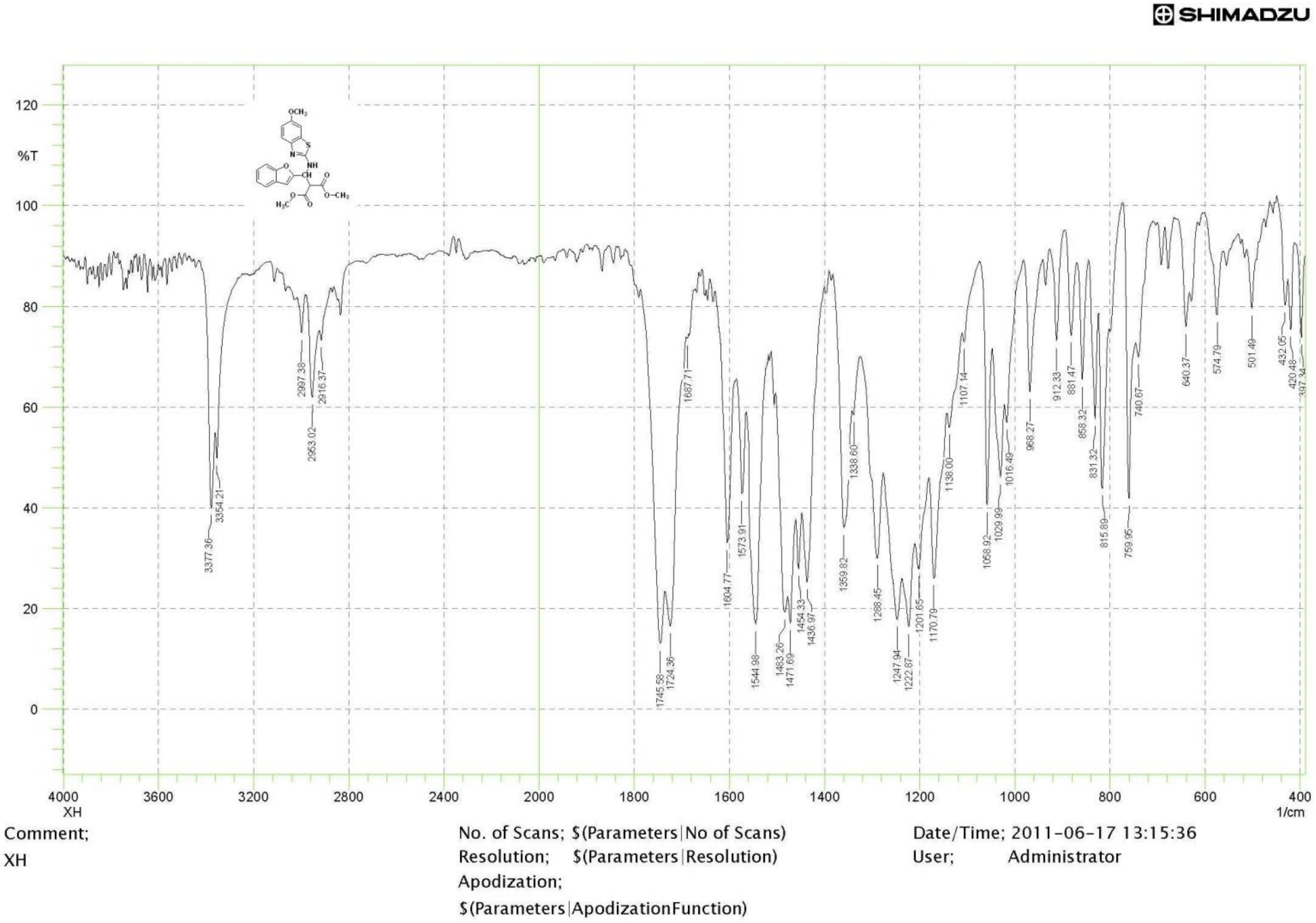

Diethyl 2-(benzofuran-2-yl((6-methoxybenzo[d]thiazol-2-yl)amino)methyl)malonate (**5j**)

Figure S37. The High Resolution Mass Spectra of **5j**.

Monoisotopic Mass, Odd and Even Electron Ions
18 formula(e) evaluated with 1 results within limits (up to 51 closest results for each mass)
Elements Used:
C: 0-200 H: 0-400 N: 2-2 O: 5-7 S: 1-1

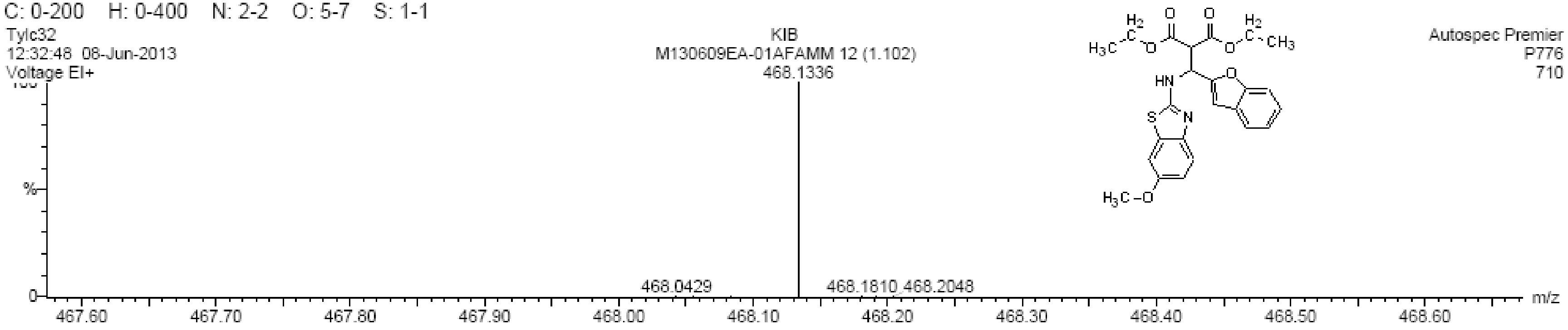

Minimum:				-10.0		
Maximum:		200.0	10.0	120.0		
Mass	Calc. Mass	mDa	PPM	DBE	i-FIT	Formula
468.1336	468.1355	-1.9	-4.1	14.0	5546370.5	C24 H24 N2 O6 S

Figure S38. The ^{1}H-NMR Spectra of **5j**.

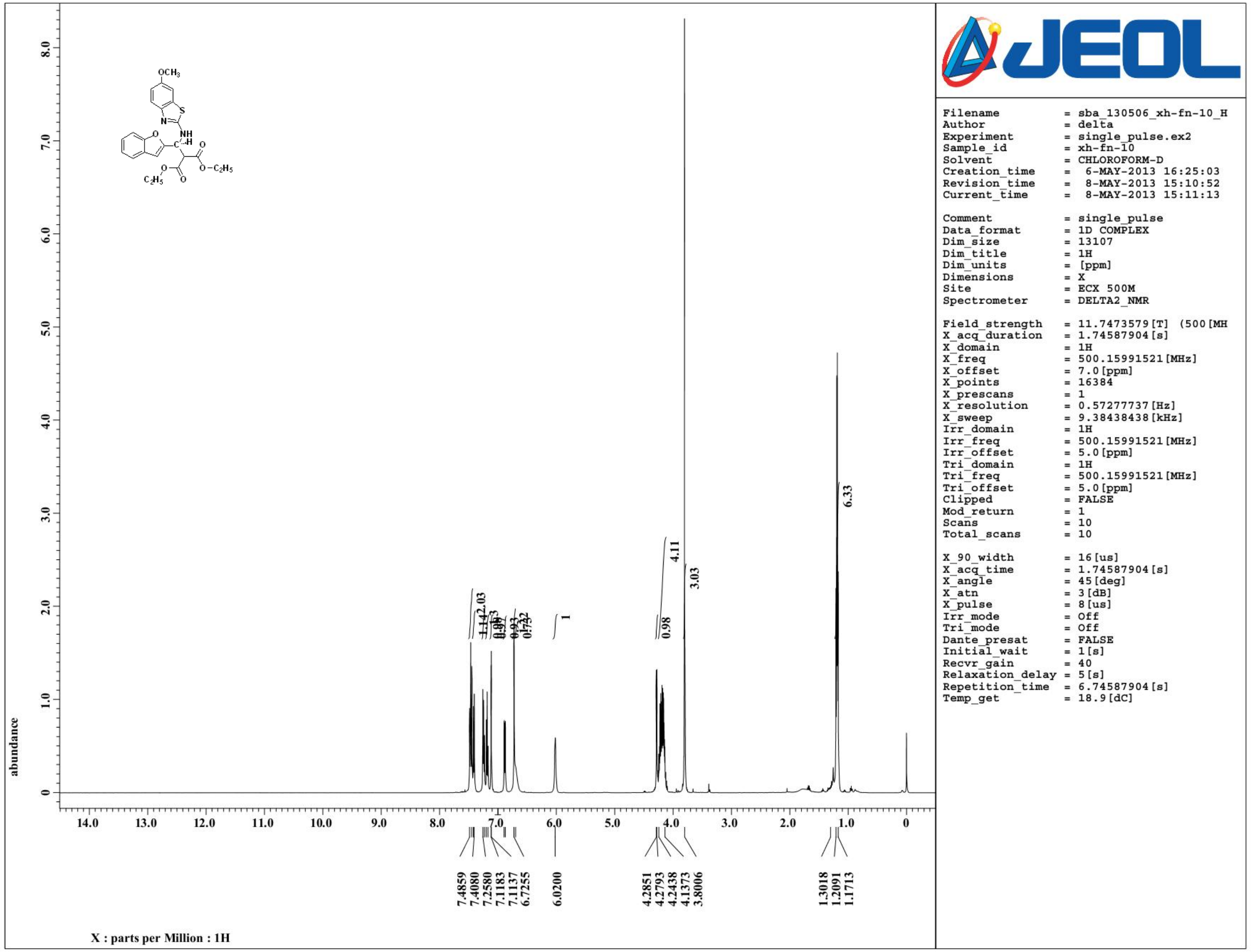

Figure S39. The ^{13}C-NMR Spectra of **5j**.

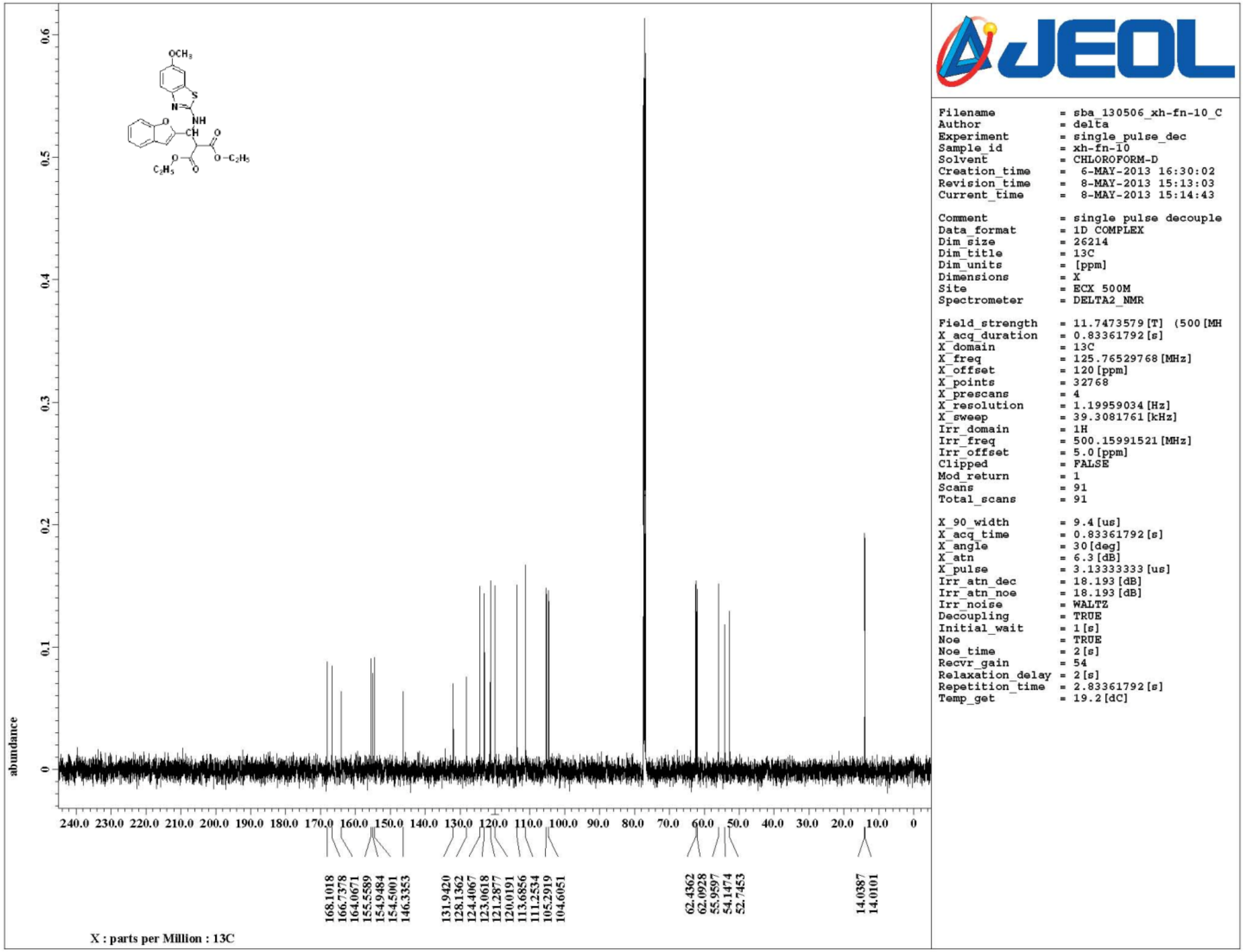

Figure S40. The IR spectra of **5j**.

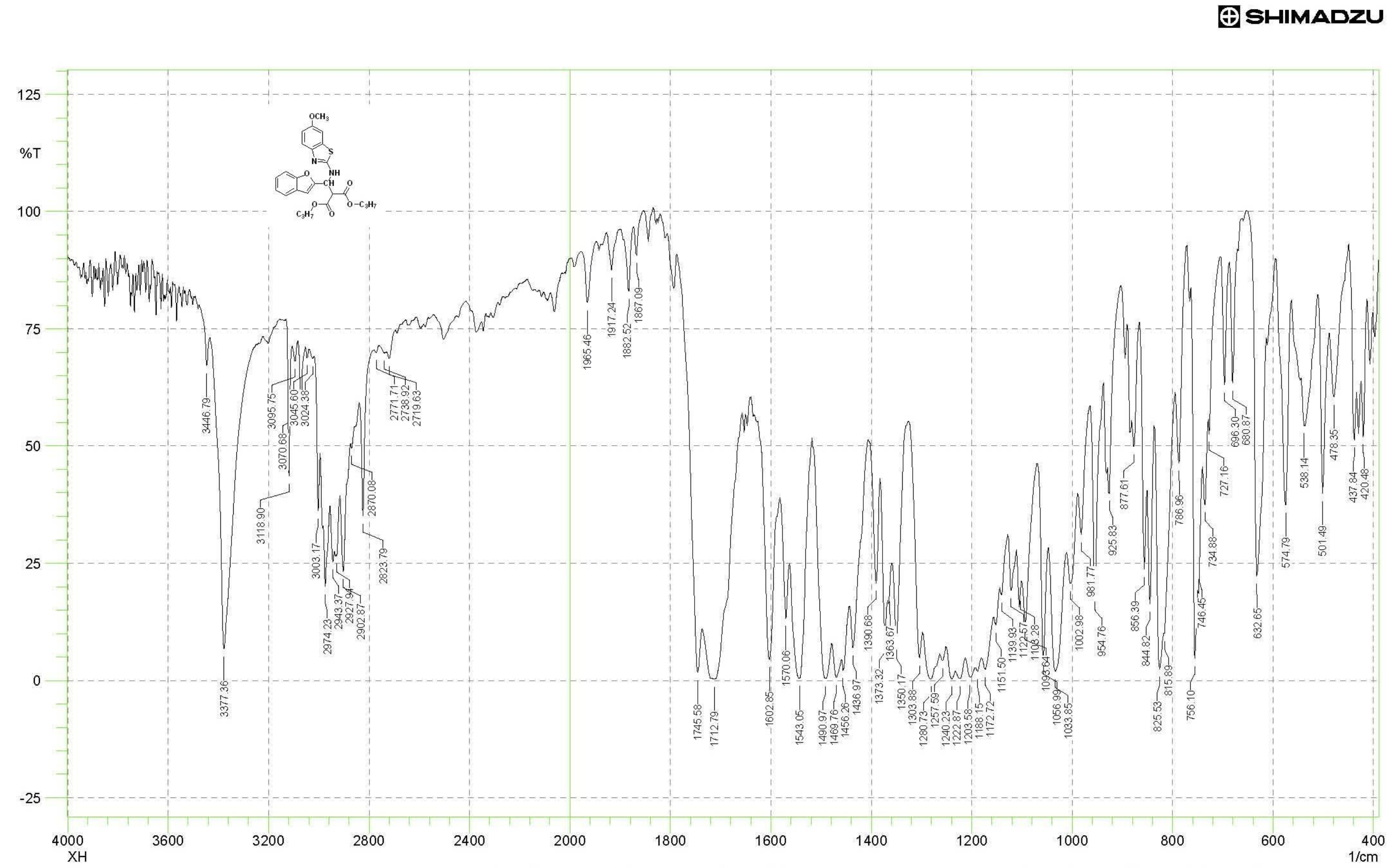

Comment;
XH

No. of Scans; $(Parameters|No of Scans)
Resolution; $(Parameters|Resolution)
Apodization;
$(Parameters|ApodizationFunction)

Date/Time; 2011-06-17 14:52:40
User; Administrator

Dipropyl 2-(benzofuran-2-yl((6-methoxybenzo[d]thiazol-2-yl)amino)methyl)malonate (**5k**)

Figure S41. The High Resolution Mass Spectra of **5k**.

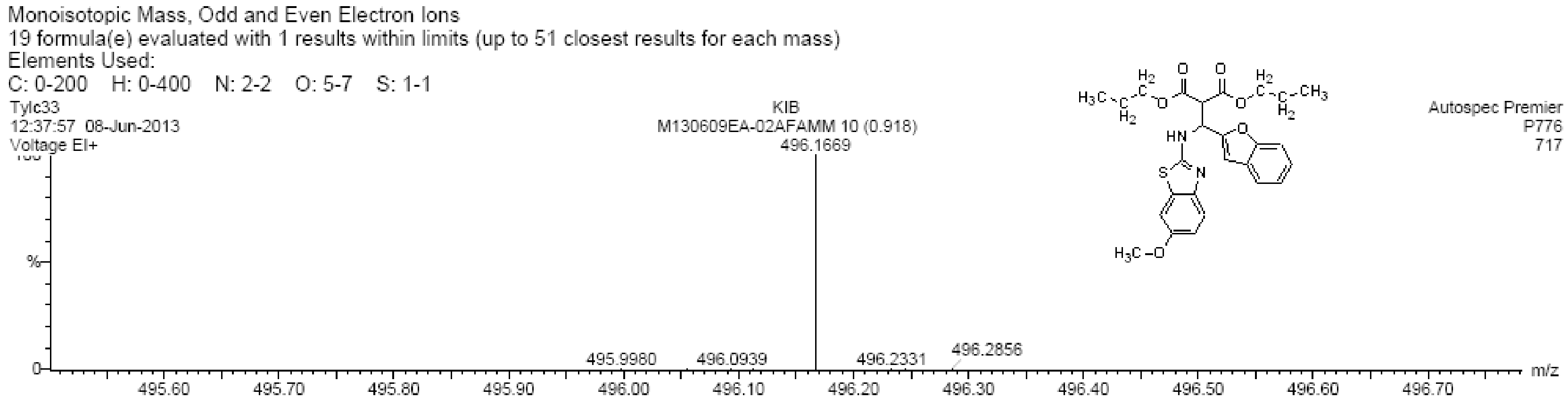

Minimum:				-10.0		
Maximum:		200.0	10.0	120.0		
Mass	Calc. Mass	mDa	PPM	DBE	i-FIT	Formula
496.1669	496.1668	0.1	0.2	14.0	5546375.0	C26 H28 N2 O6 S

Figure S42. The ^{1}H-NMR Spectra of **5k**.

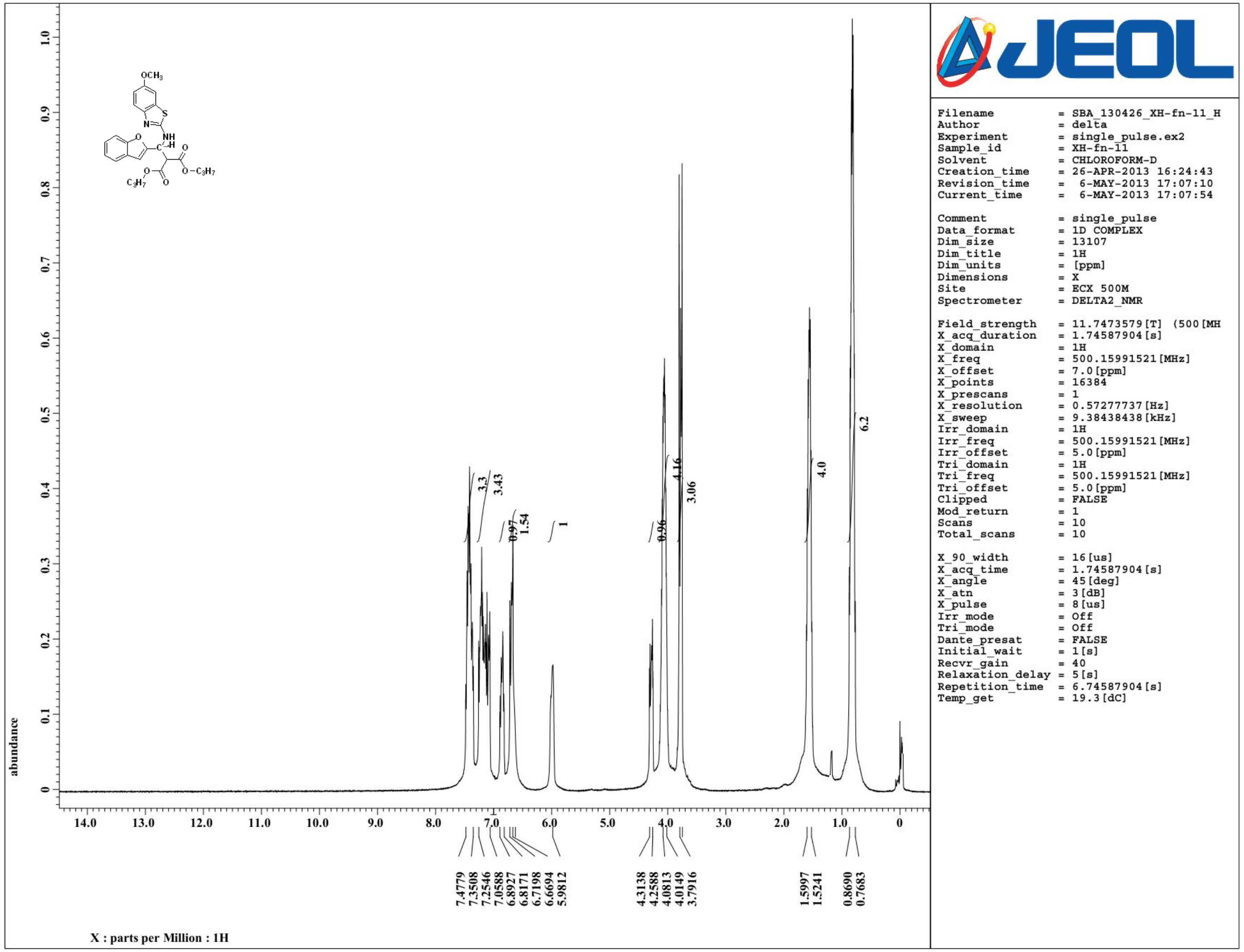

Figure S43. The ^{13}C-NMR Spectra of **5k**.

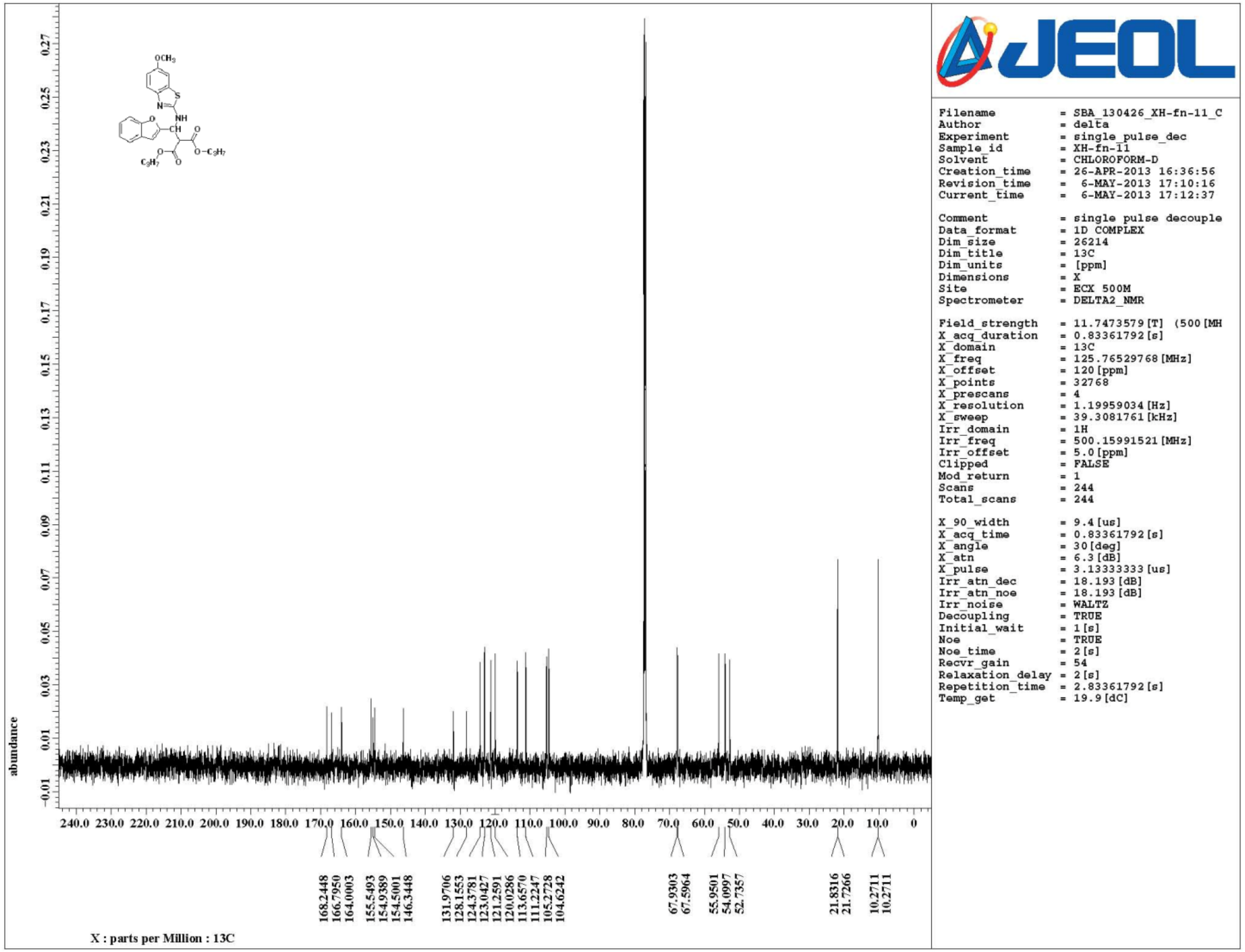

Figure S44. The IR spectra of **5k**.

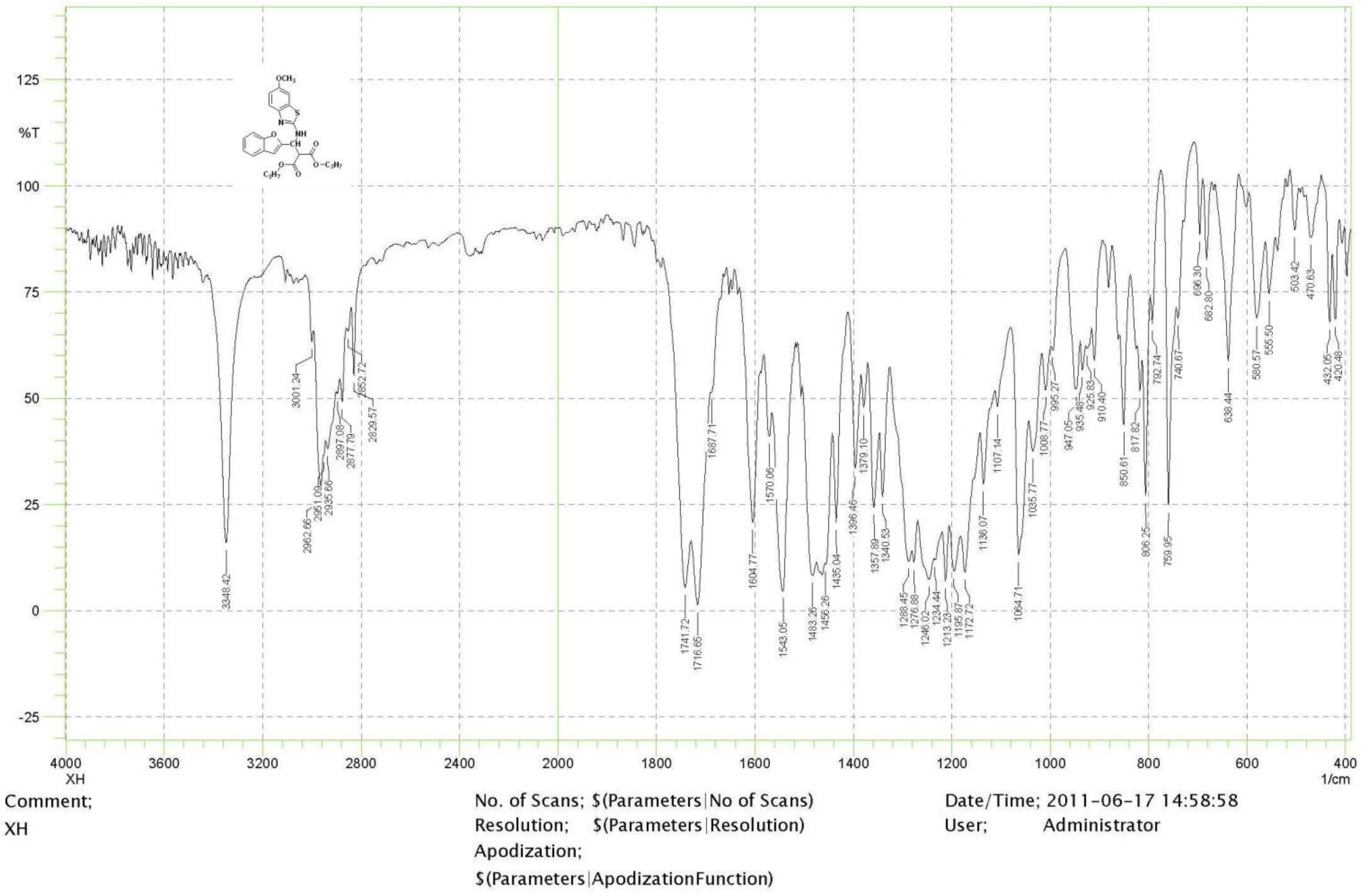

Dibenzyl 2-(benzofuran-2-yl((6-methoxybenzo[d]thiazol-2-yl)amino)methyl)malonate (**5l**)

Figure S45. The High Resolution Mass Spectra of **5l**.

Monoisotopic Mass, Odd and Even Electron Ions
23 formula(e) evaluated with 1 results within limits (up to 51 closest results for each mass)
Elements Used:
C: 0-200 H: 0-400 N: 2-2 O: 4-6 S: 1-1

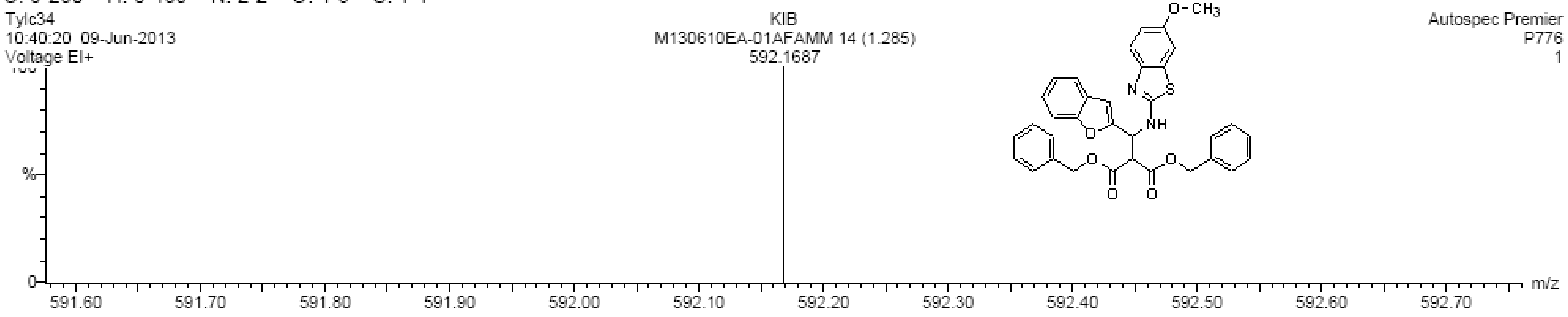

Mass	Calc. Mass	mDa	PPM	DBE	i-FIT	Formula
Minimum:				-10.0		
Maximum:		10.0	10.0	120.0		
592.1687	592.1668	1.9	3.2	22.0	5546026.0	C34 H28 N2 O6 S

Figure S46. The ^{1}H-NMR Spectra of **5l**.

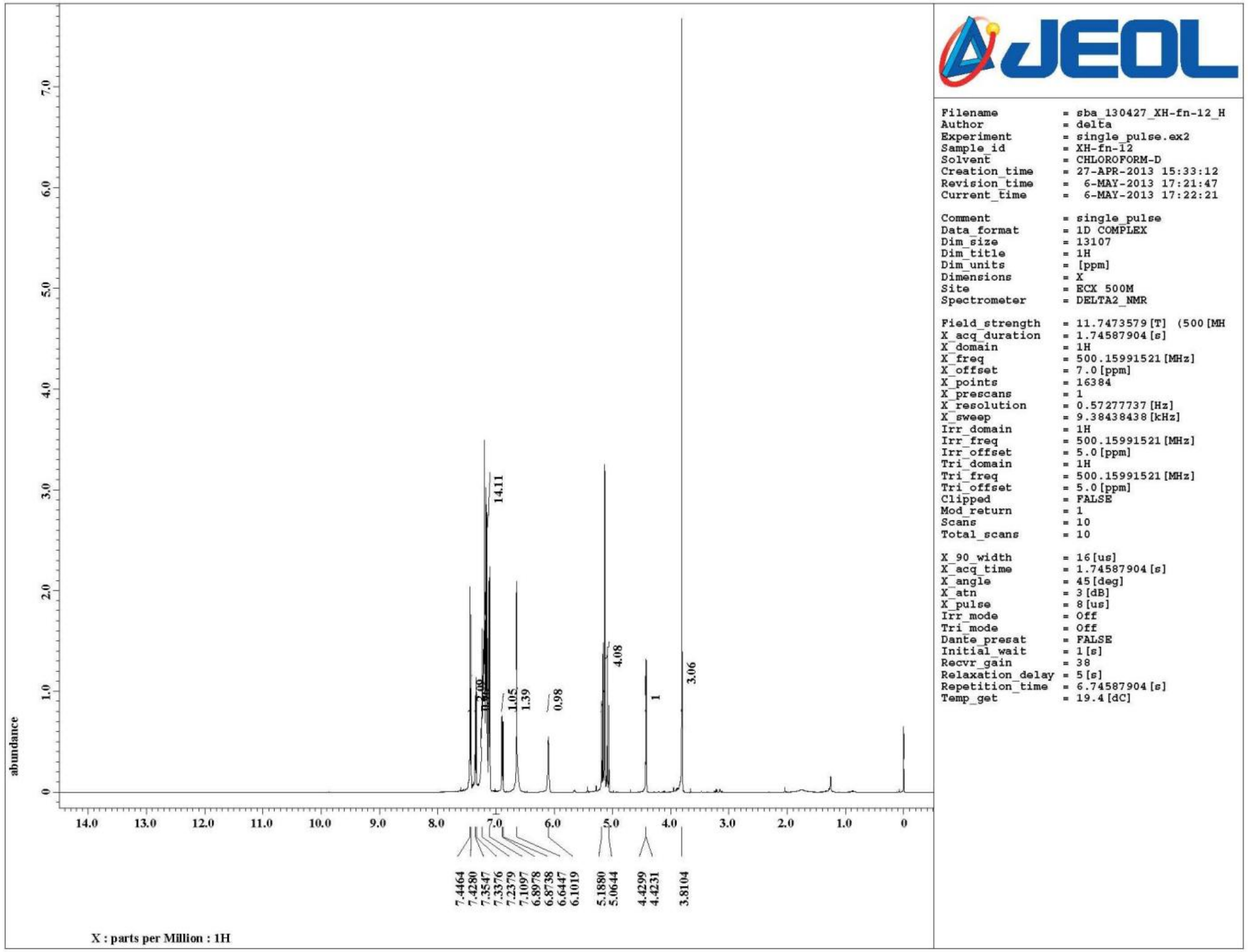

Figure S47. The ^{13}C-NMR Spectra of **5l**.

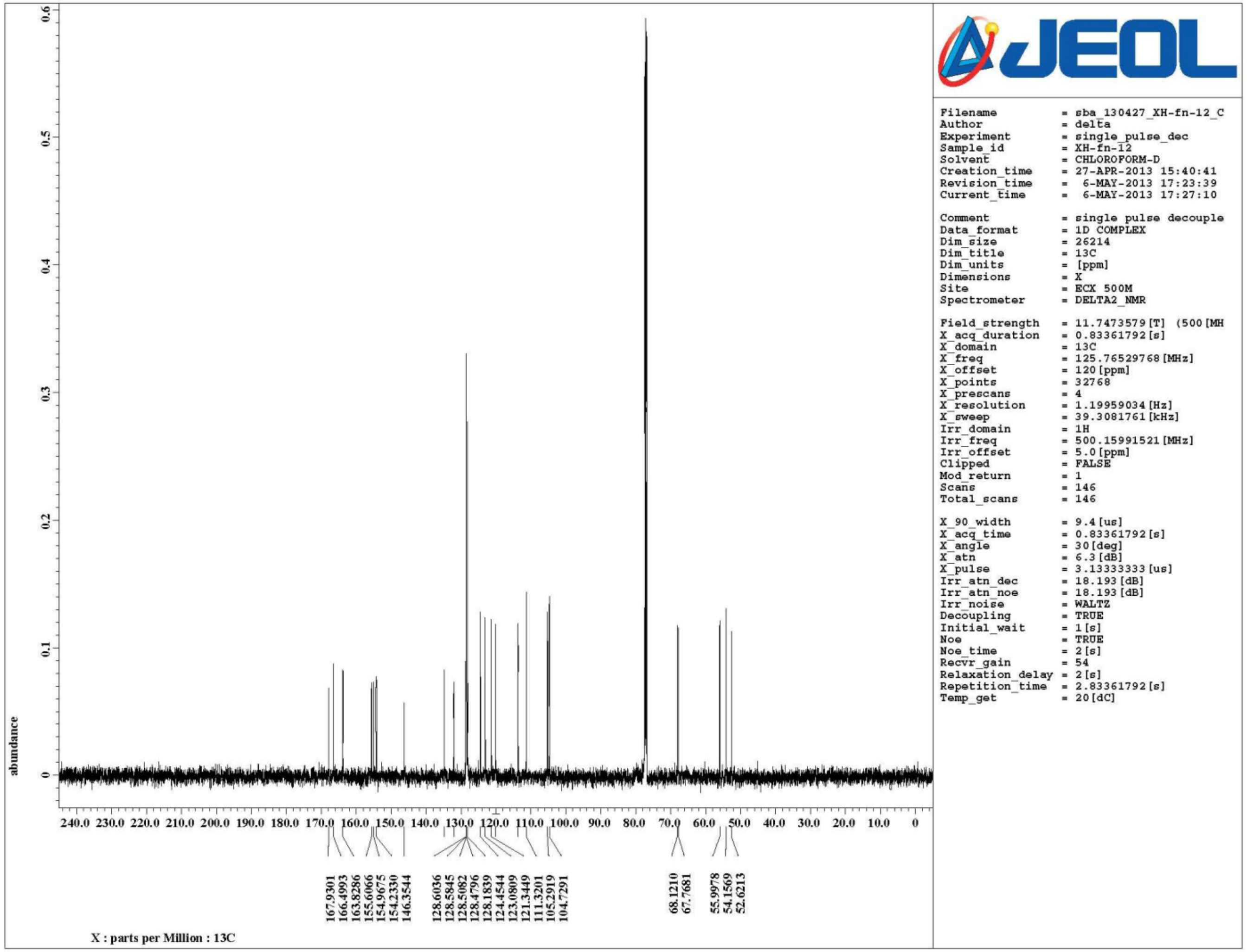

Figure S48. The IR spectra of **5l**.

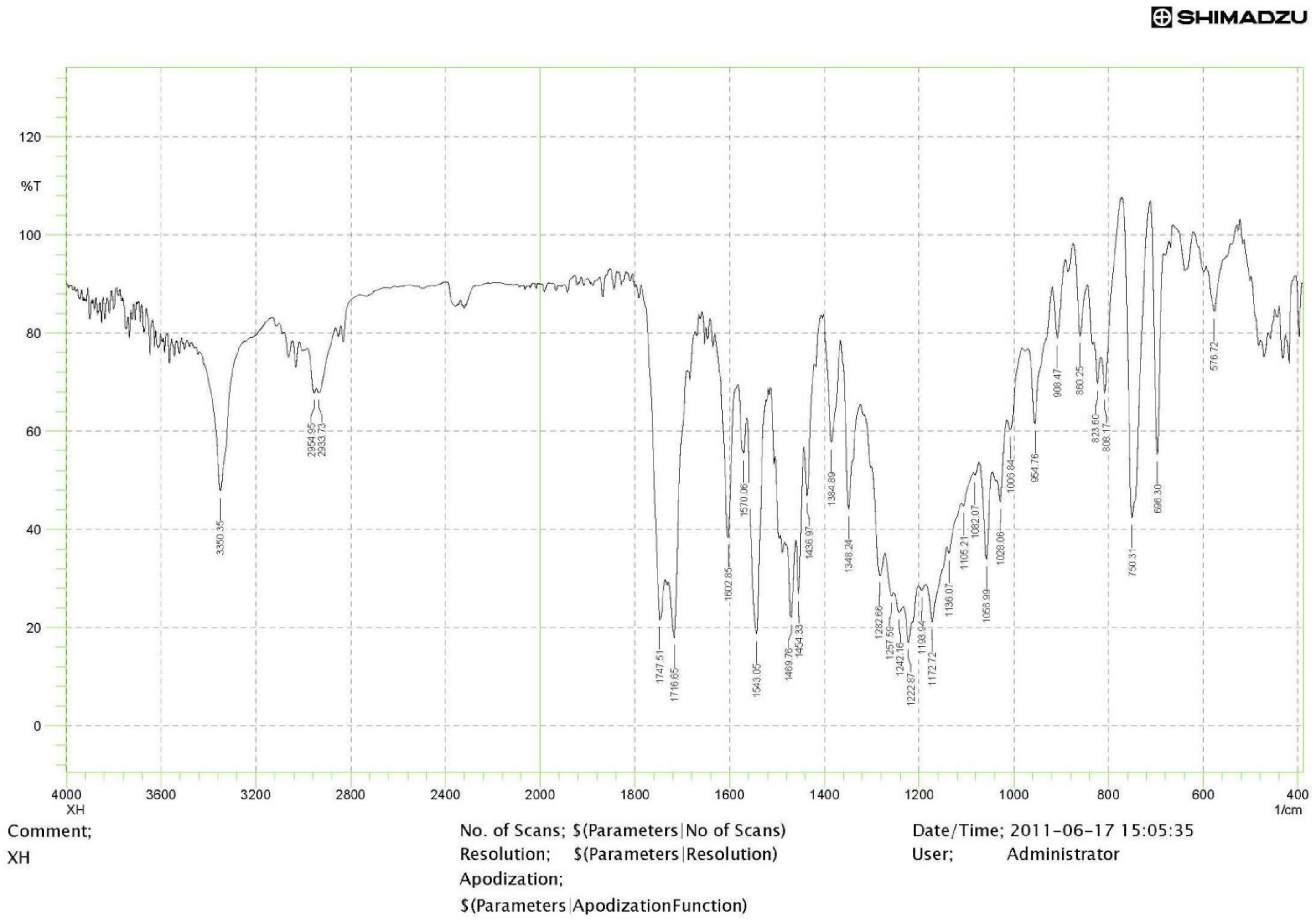

Dimethyl 2-(benzofuran-2-yl((6-methylbenzo[d]thiazol-2-yl)amino)methyl)malonate (**5m**)

Figure S49. The High Resolution Mass Spectra of **5m**.

Monoisotopic Mass, Odd and Even Electron Ions
17 formula(e) evaluated with 1 results within limits (up to 51 closest results for each mass)
Elements Used:
C: 0-200 H: 0-400 N: 2-2 O: 5-7 S: 1-1

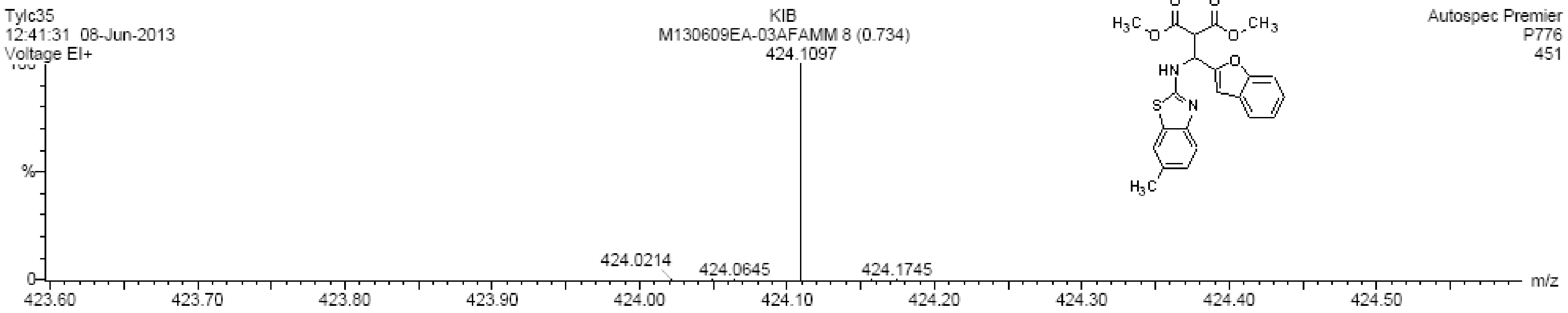

Minimum:				-10.0		
Maximum:		200.0	10.0	120.0		
Mass	Calc. Mass	mDa	PPM	DBE	i-FIT	Formula
424.1097	424.1093	0.4	0.9	14.0	5546240.0	C22 H20 N2 O5 S

Figure S50. The ^{1}H-NMR Spectra of **5m**.

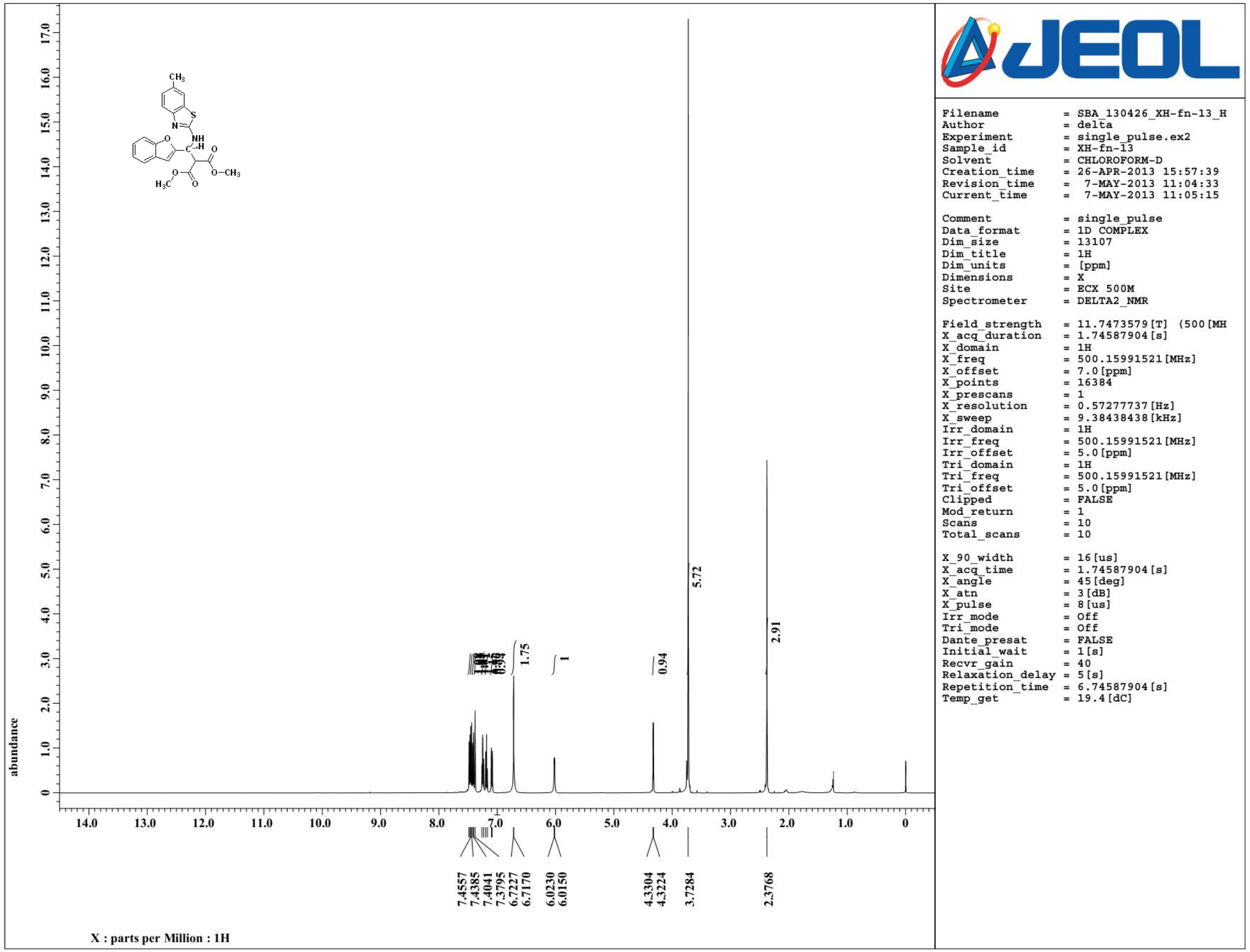

Figure S51. The ^{13}C-NMR Spectra of **5m**.

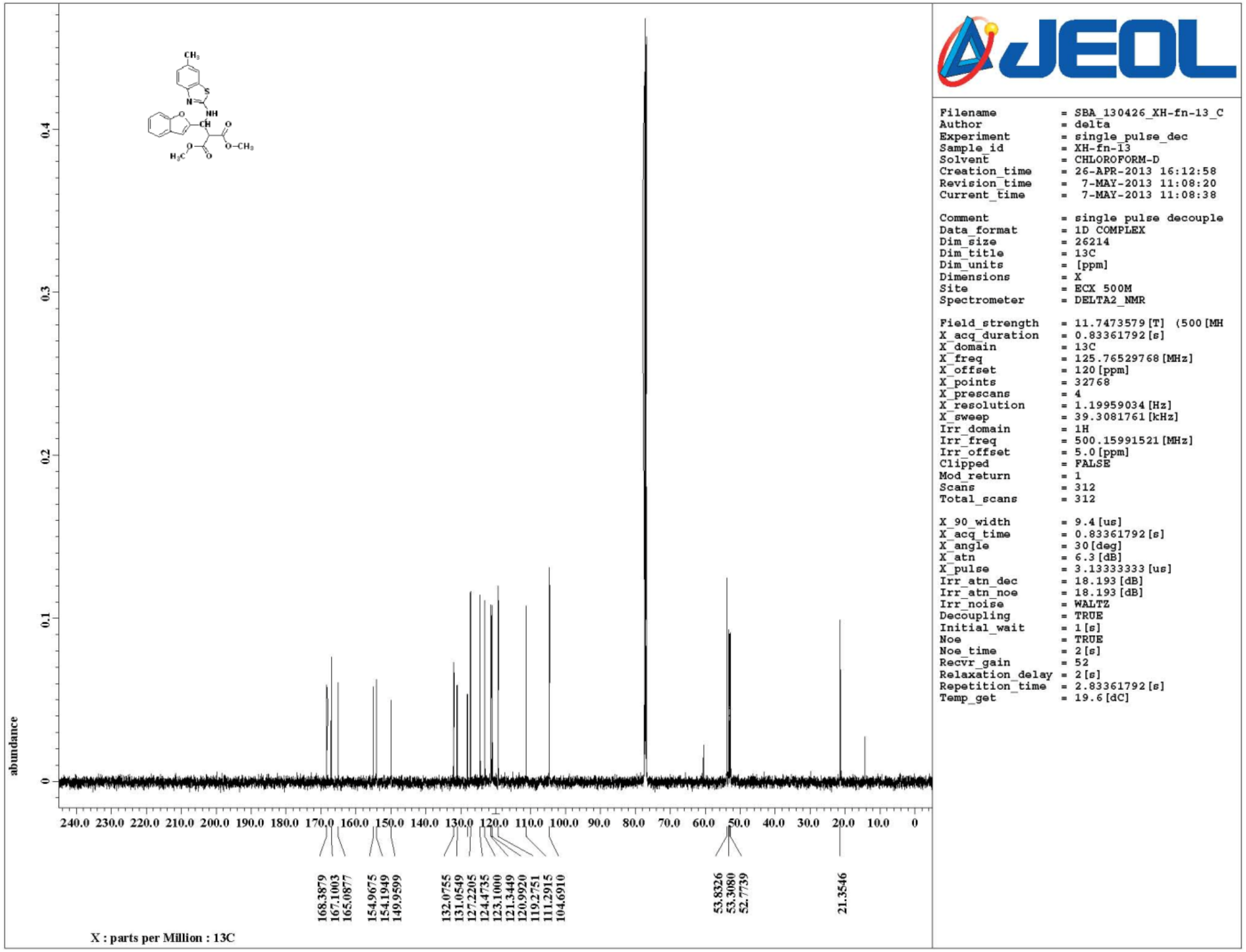

Figure S52. The IR spectra of **5m**.

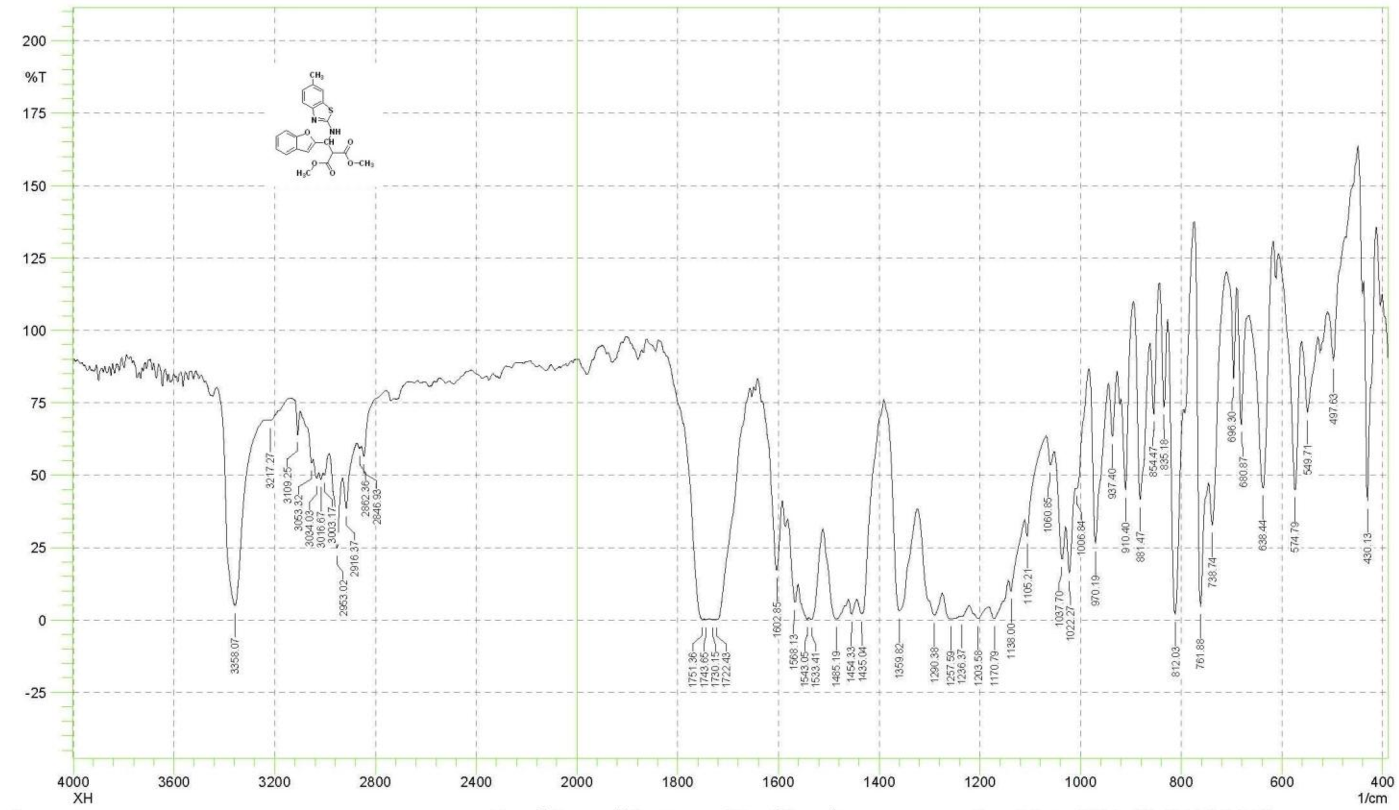

Comment;
XH

No. of Scans; $(Parameters|No of Scans)
Resolution; $(Parameters|Resolution)
Apodization;
$(Parameters|ApodizationFunction)

Date/Time; 2011-06-17 13:39:29
User; Administrator

Diethyl 2-(benzofuran-2-yl((6-methylbenzo[d]thiazol-2-yl)amino)methyl)malonate (**5n**)

Figure S53. The High Resolution Mass Spectra of **5n**.

Monoisotopic Mass, Odd and Even Electron Ions
18 formula(e) evaluated with 1 results within limits (up to 51 closest results for each mass)
Elements Used:
C: 0-200 H: 0-400 N: 2-2 O: 5-7 S: 1-1

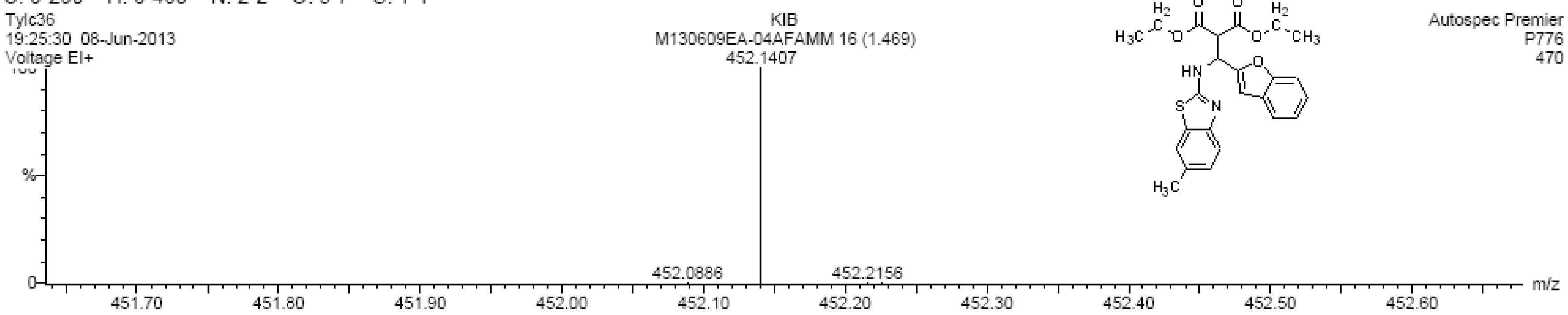

Minimum:				-10.0		
Maximum:		200.0	10.0	120.0		
Mass	Calc. Mass	mDa	PPM	DBE	i-FIT	Formula
452.1407	452.1406	0.1	0.2	14.0	5546251.0	C24 H24 N2 O5 S

Figure S54. The ^{1}H-NMR Spectra of **5n**.

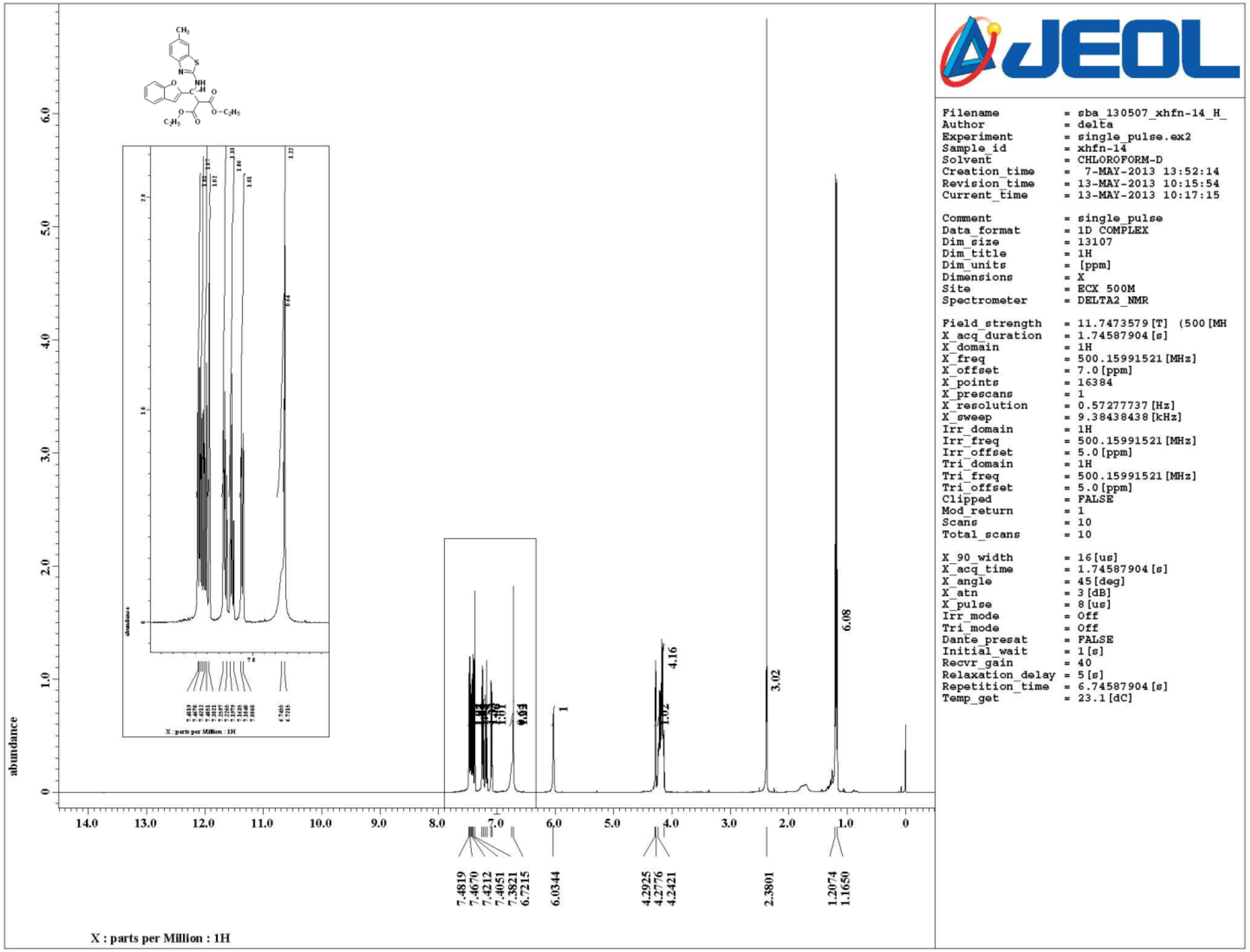

Figure S55. The ^{13}C-NMR Spectra of **5n**.

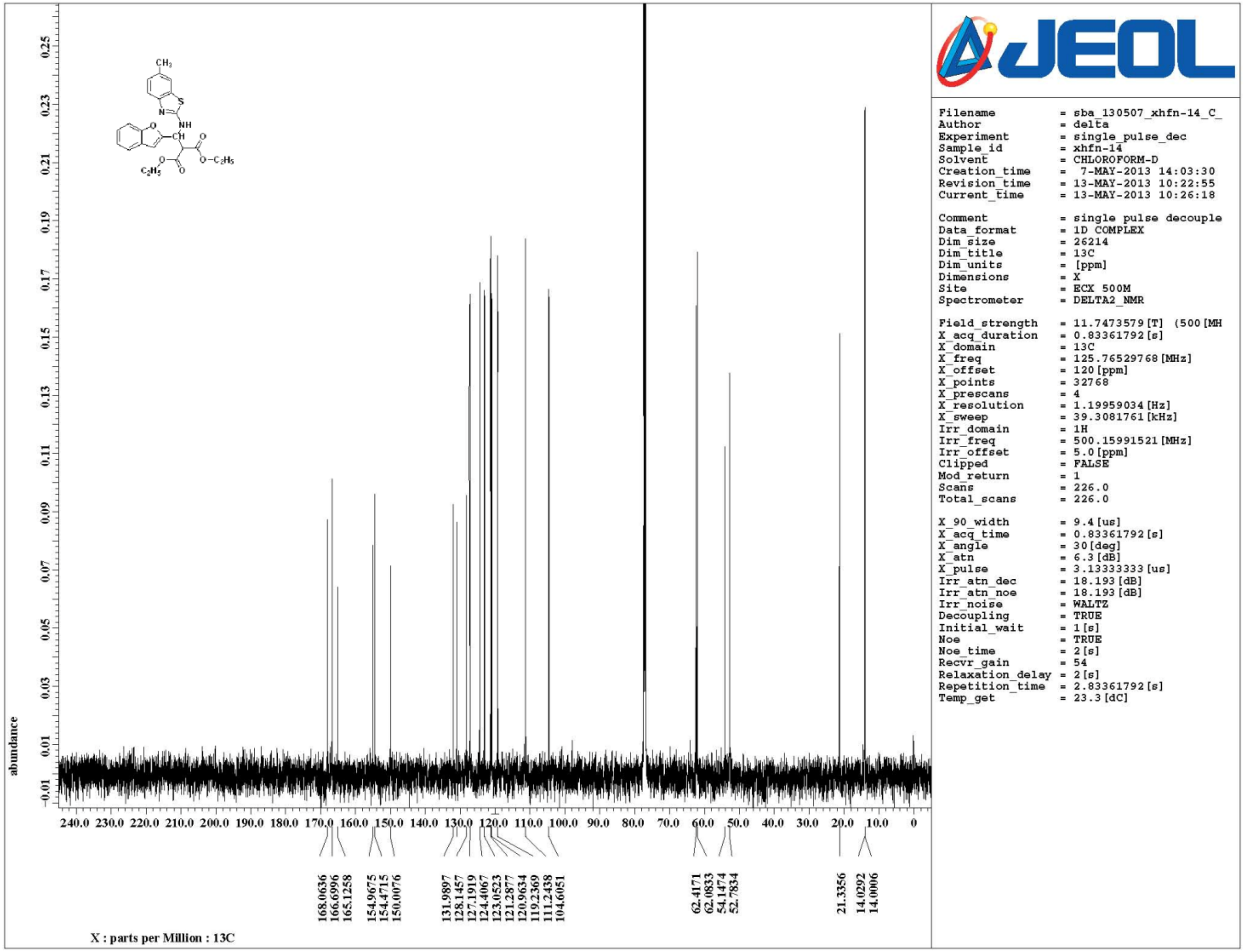

Figure S56. The IR spectra of **5n**.

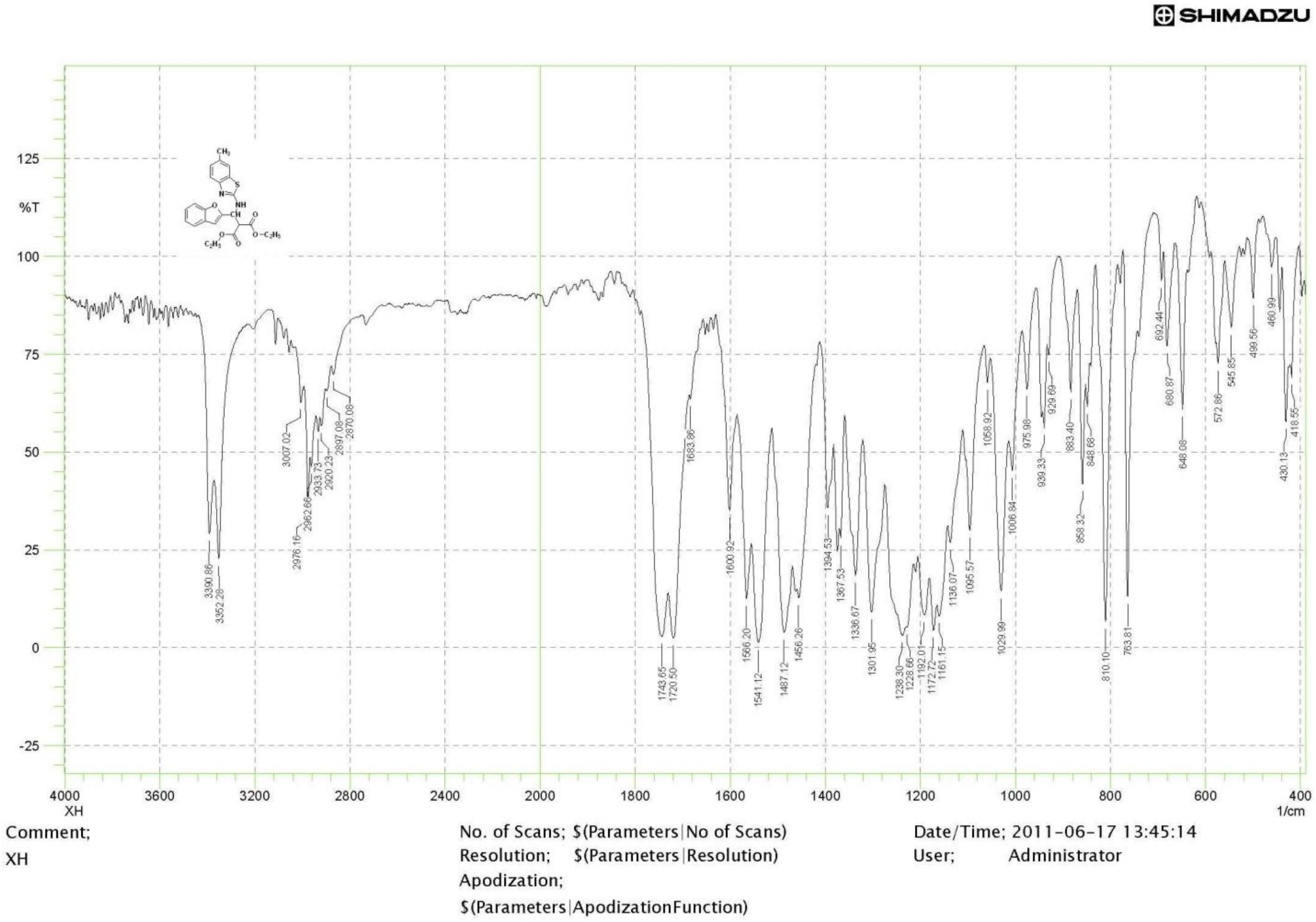

Dipropyl 2-(benzofuran-2-yl((6-methylbenzo[d]thiazol-2-yl)amino)methyl)malonate (**5o**)

Figure S57. The High Resolution Mass Spectra of **5o**.

Monoisotopic Mass, Odd and Even Electron Ions
19 formula(e) evaluated with 1 results within limits (up to 51 closest results for each mass)
Elements Used:
C: 0-200 H: 0-400 N: 2-2 O: 5-7 S: 1-1

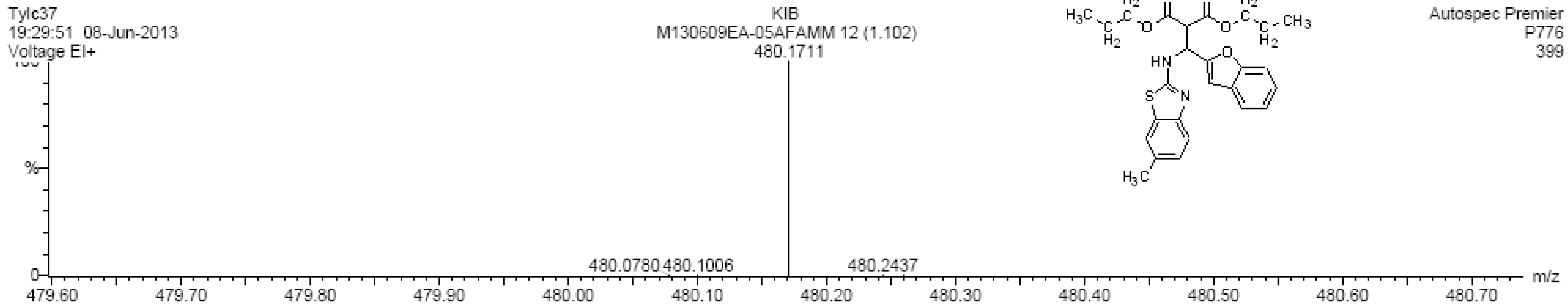

Minimum:				-10.0		
Maximum:		200.0	10.0	120.0		
Mass	Calc. Mass	mDa	PPM	DBE	i-FIT	Formula
480.1711	480.1719	-0.8	-1.7	14.0	5546216.5	C26 H28 N2 O5 S

Figure S58. The ^{1}H-NMR Spectra of **5o**.

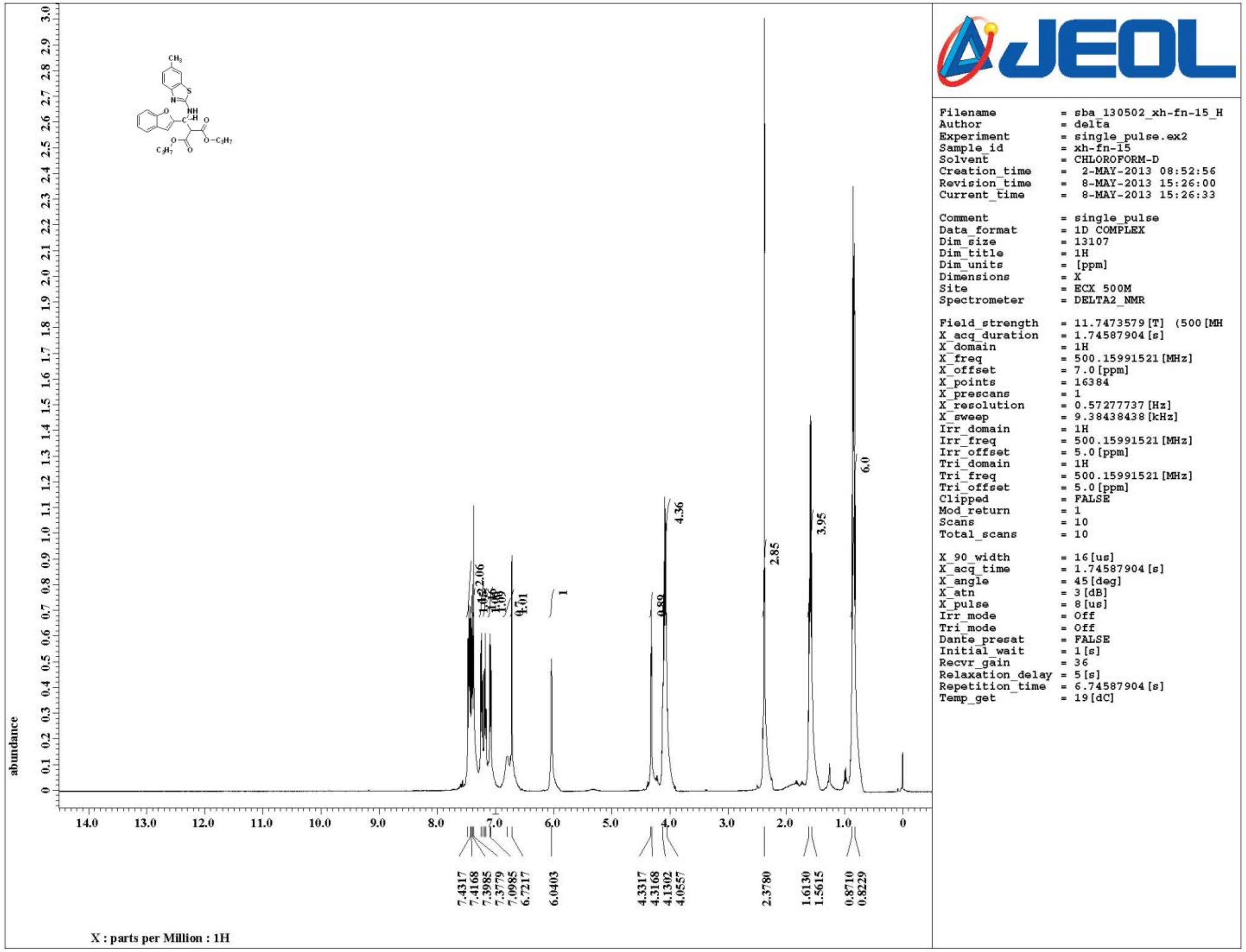

Figure S59. The ^{13}C-NMR Spectra of **5o**.

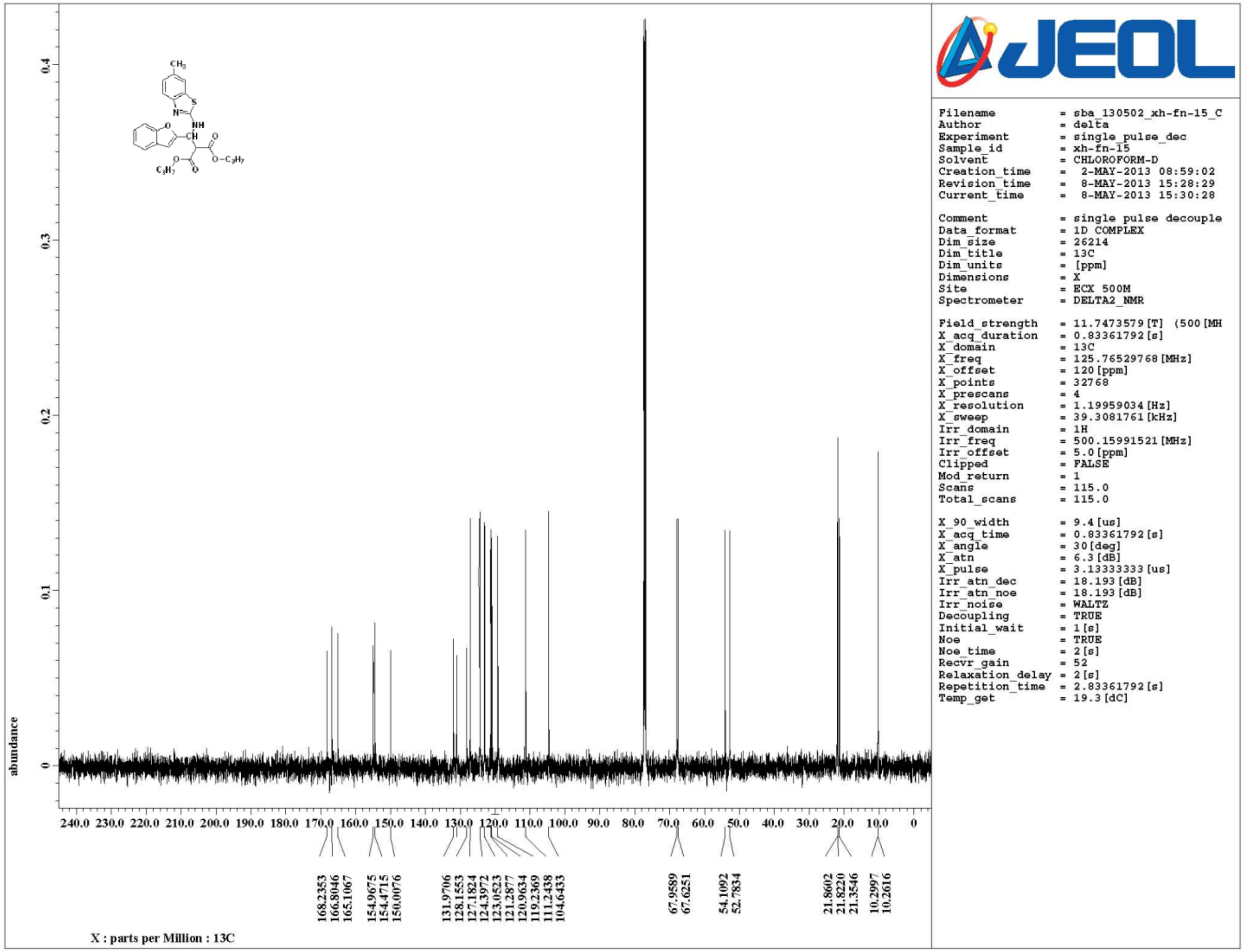

Figure S60. The IR spectra of **5o**.

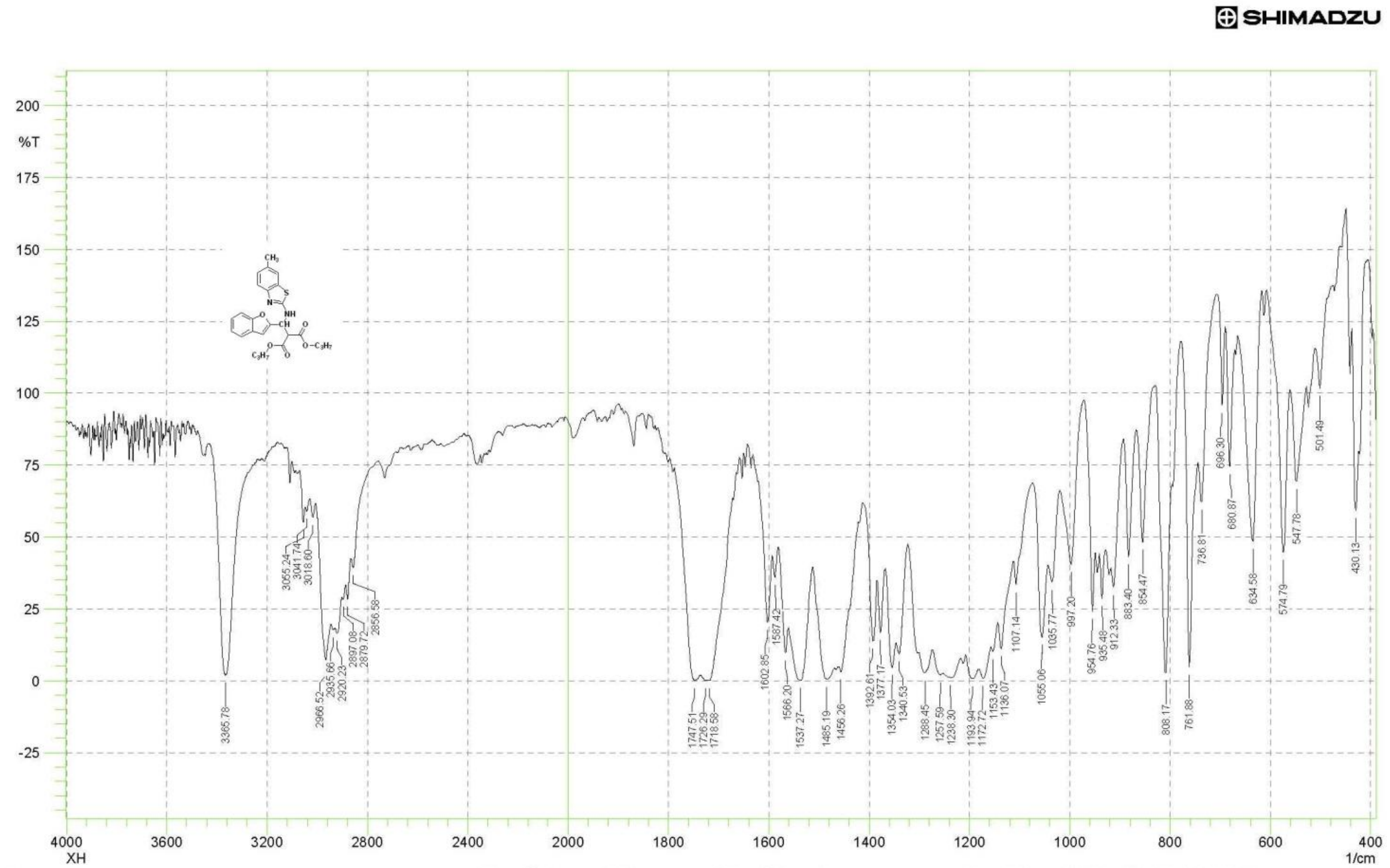

Comment;
XH

No. of Scans; $(Parameters|No of Scans)
Resolution; $(Parameters|Resolution)
Apodization;
$(Parameters|ApodizationFunction)

Date/Time; 2011-06-17 15:26:47
User; Administrator

Dibenzyl 2-(benzofuran-2-yl((6-methylbenzo[d]thiazol-2-yl)amino)methyl) malonate (**5p**)

Figure S61. The High Resolution Mass Spectra of **5p**.

Monoisotopic Mass, Odd and Even Electron Ions
23 formula(e) evaluated with 1 results within limits (up to 51 closest results for each mass)
Elements Used:
C: 0-200 H: 0-400 N: 2-2 O: 4-6 S: 1-1

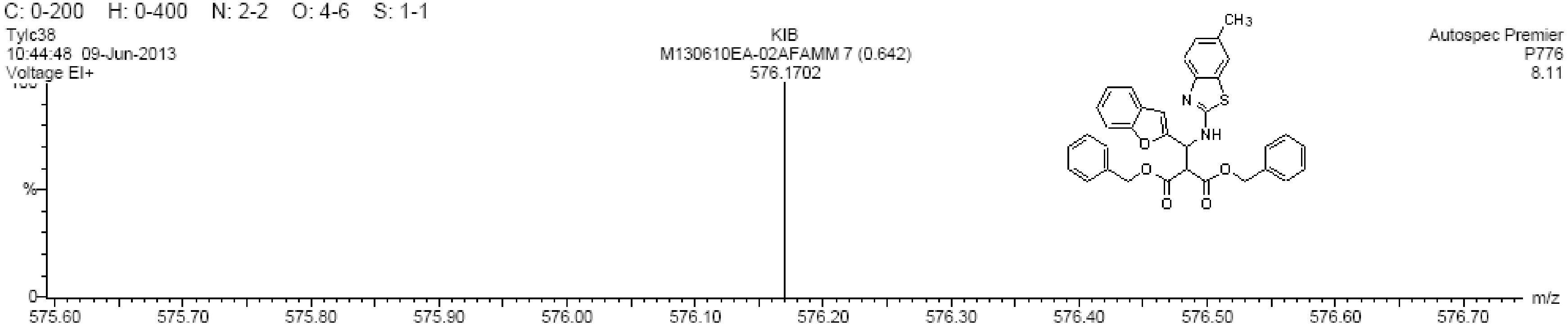

Minimum:				-10.0		
Maximum:		10.0	10.0	120.0		
Mass	Calc. Mass	mDa	PPM	DBE	i-FIT	Formula
576.1702	576.1719	-1.7	-3.0	22.0	5546026.0	C34 H28 N2 O5 S

Figure S62. The ^{1}H-NMR Spectra of **5p**.

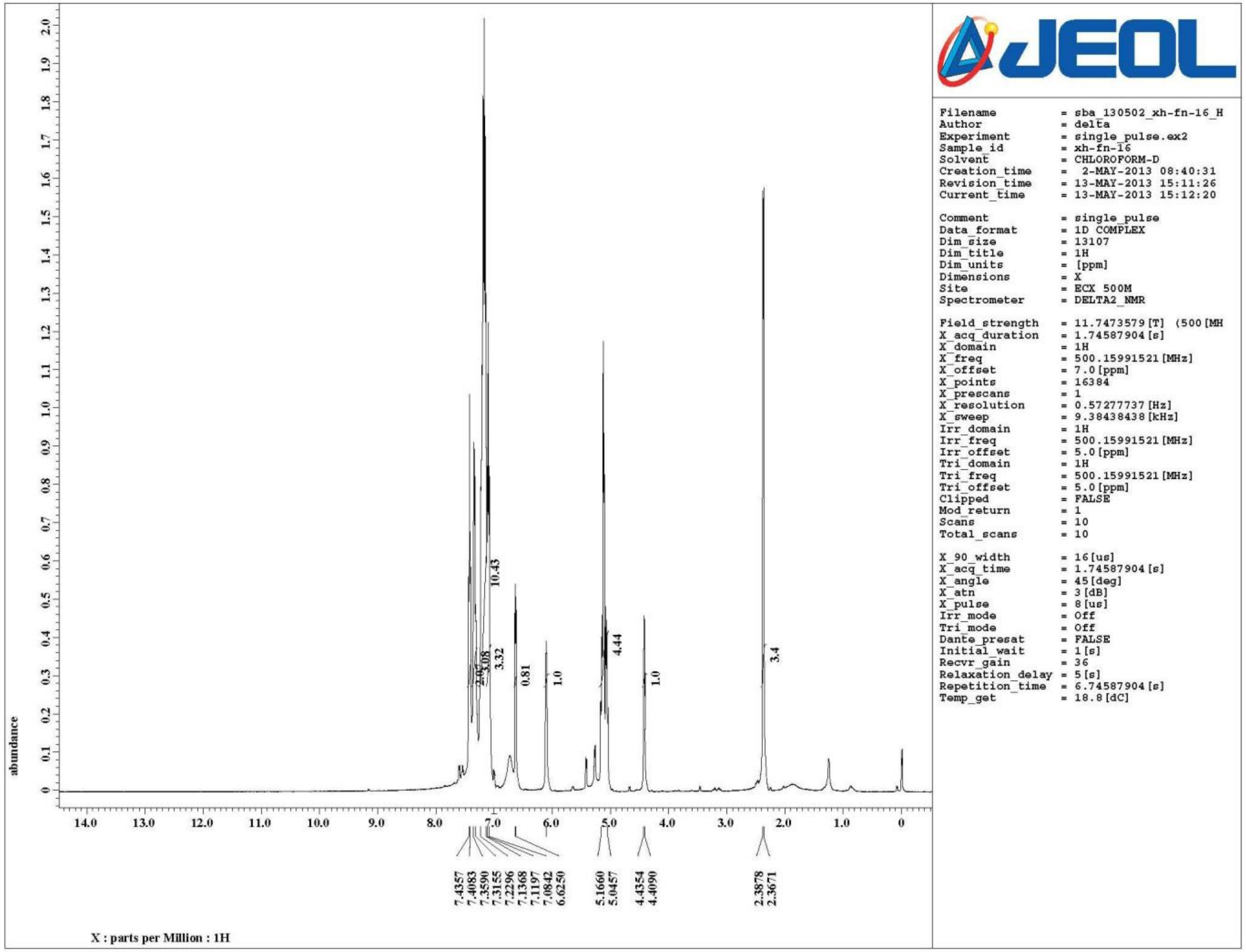

Figure S63. The ^{13}C-NMR Spectra of **5p**.

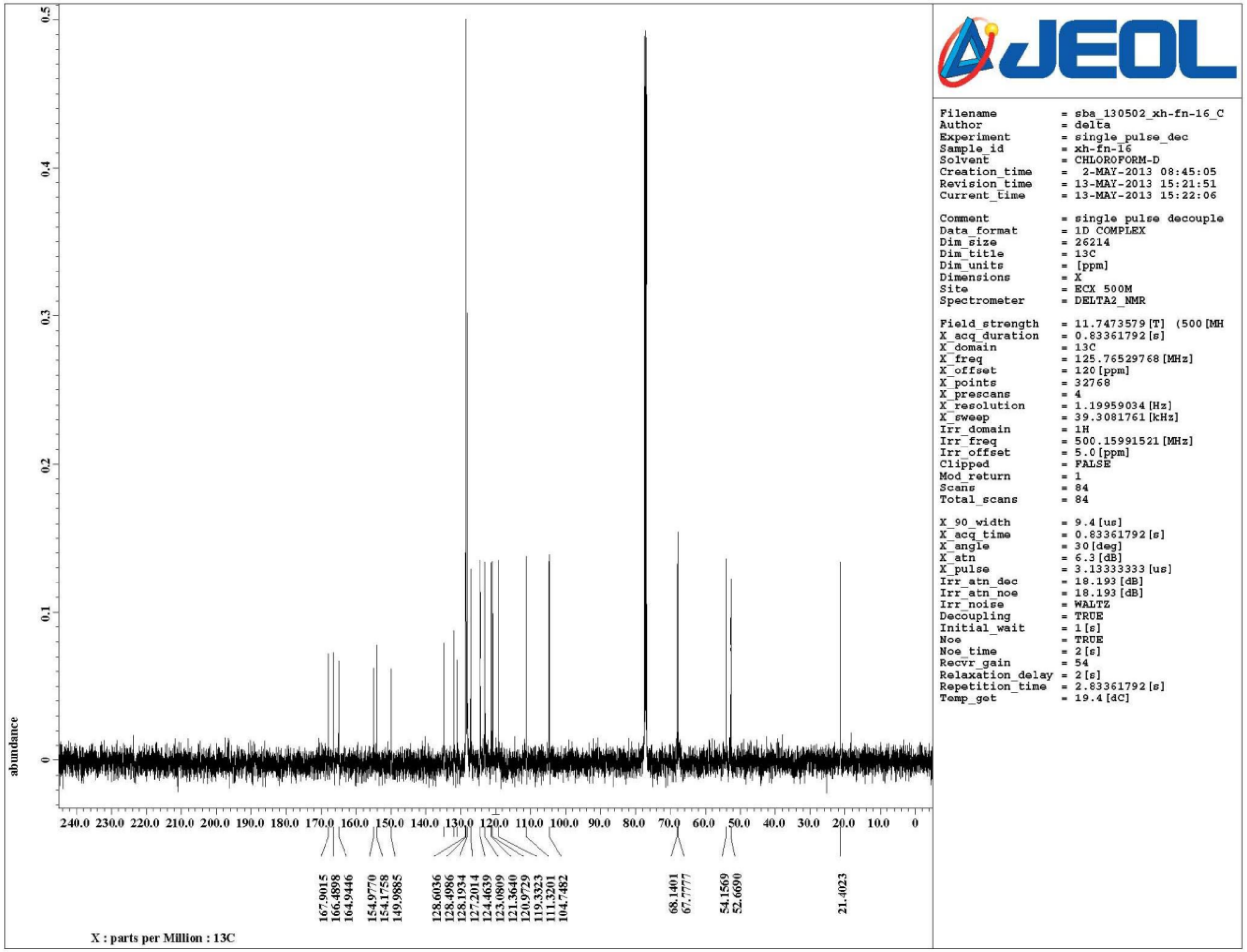

Figure S64. The IR spectra of **5p**.

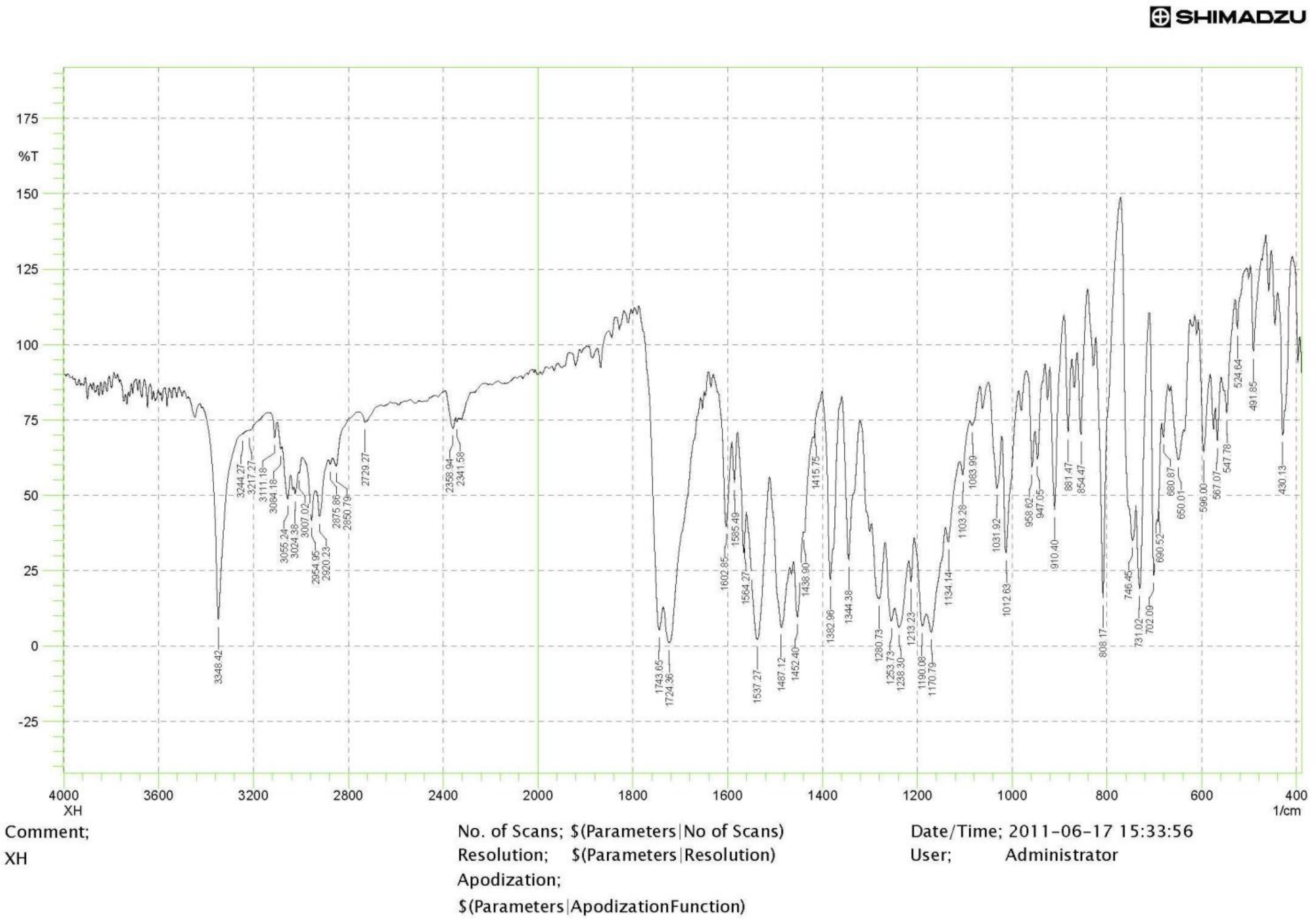

Supplement: Supplementary file 1 [file molecules-18-13623-s001.pdf]
